# Complete Genome Sequence of *Bradyrhizobium* sp. S23321: Insights into Symbiosis Evolution in Soil Oligotrophs

**DOI:** 10.1264/jsme2.ME11321

**Published:** 2012-03-28

**Authors:** Takashi Okubo, Takahiro Tsukui, Hiroko Maita, Shinobu Okamoto, Kenshiro Oshima, Takatomo Fujisawa, Akihiro Saito, Hiroyuki Futamata, Reiko Hattori, Yumi Shimomura, Shin Haruta, Sho Morimoto, Yong Wang, Yoriko Sakai, Masahira Hattori, Shin-ichi Aizawa, Kenji V. P. Nagashima, Sachiko Masuda, Tsutomu Hattori, Akifumi Yamashita, Zhihua Bao, Masahito Hayatsu, Hiromi Kajiya-Kanegae, Ikuo Yoshinaga, Kazunori Sakamoto, Koki Toyota, Mitsuteru Nakao, Mitsuyo Kohara, Mizue Anda, Rieko Niwa, Park Jung-Hwan, Reiko Sameshima-Saito, Shin-ichi Tokuda, Sumiko Yamamoto, Syuji Yamamoto, Tadashi Yokoyama, Tomoko Akutsu, Yasukazu Nakamura, Yuka Nakahira-Yanaka, Yuko Takada Hoshino, Hideki Hirakawa, Hisayuki Mitsui, Kimihiro Terasawa, Manabu Itakura, Shusei Sato, Wakako Ikeda-Ohtsubo, Natsuko Sakakura, Eli Kaminuma, Kiwamu Minamisawa

**Affiliations:** 1 Graduate School of Life Sciences, Tohoku University, 2–1–1 Katahira, Aoba-ku, Sendai, Miyagi 980–8577, Japan; 2 Laboratory for Plant Genome Informatics, Kazusa DNA Research Institute, 2–6–7 Kazusakamatari, Kisarazu, Chiba 292–0818, Japan; 3 Database Center for Life Science (DBCLS), Research Organization of Information and Systems (ROIS), 2–11–16 Yayoi, Bunkyo-ku, Tokyo 113–0032, Japan; 4 Graduate School of Frontier Sciences, University of Tokyo, 5–1–5, Kashiwa-no-ha, Kashiwa, Chiba 277–8561, Japan; 5 Center for Information Biology and DNA Data Bank of Japan, National Institute of Genetics, Research Organization for Information and Systems, Yata, Mishima, Shizuoka 411–85, Japan; 6 Department of Material and Life Science, Faculty of Science and Technology, Shizuoka Institute of Science and Technology 2200–2 Toyosawa, Fukuroi, Shizuoka 437–8555, Japan; 7 Department of Material Science and Chemical Engineering, Shizuoka University, 3–5–1 Jyohoku, Naka-ku, Hamamatsu, Shizuoka, 432–8561, Japan; 8 Attic Lab, 1–6–2–401 Komegafukuro, Aobaku, Sendai, Miyagi 980–0813, Japan; 9 National Institute for Agro-Environmental Sciences, 3–1–3 Kannondai, Tsukuba, Ibaraki 305–8604, Japan; 10 Graduate School of Science and Engineering, Tokyo Metropolitan University, 1–1 Minami-Osawa, Hachioji-shi, Tokyo 192–0397, Japan; 11 Department of Life Sciences, Prefectural University of Hiroshima, 562 Nanatsuka, Shobara, Hiroshima 727–0023, Japan; 12 Graduate School of Agriculture, Kyoto University, Oiwake-cho, Kitashirakawa, Sakyo-ku, Kyoto 606–8502, Japan; 13 Graduate School of Horticulture, Chiba University, 648 Matsudo, Matsudo, Chiba 271–8510, Japan; 14 Tokyo University of Agriculture and Technology, 2–24–16, Naka, Koganei, Tokyo 184–8588, Japan; 15 Faculty of Agriculture, Shizuoka University, 836 Ohya, Suruga-ku, Shizuoka 422–8529, Japan; 16 National Institute of Vegetable and Tea Sciences, National Agriculture and Food Research Organization, 3–1–1 Kannondai, Tsukuba, Ibaraki 305–8666, Japan; 17 Institute of Agriculture, Tokyo university of Agriculture and Technology, 3–5–8 Saiwaicho, Fuchu, Tokyo 183–8509, Japan; 18 Graduate School of Life and Environment Sciences, University of Tsukuba, 1–1–1 Ten-noudai, Tsukuba, Ibaraki 305–8572, Japan

**Keywords:** *Bradyrhizobium* sp. S23321, comparative genomics, symbiosis evolution, photosynthesis, oligotrophic soil bacterium

## Abstract

*Bradyrhizobium* sp. S23321 is an oligotrophic bacterium isolated from paddy field soil. Although S23321 is phylogenetically close to *Bradyrhizobium japonicum* USDA110, a legume symbiont, it is unable to induce root nodules in siratro, a legume often used for testing Nod factor-dependent nodulation. The genome of S23321 is a single circular chromosome, 7,231,841 bp in length, with an average GC content of 64.3%. The genome contains 6,898 potential protein-encoding genes, one set of rRNA genes, and 45 tRNA genes. Comparison of the genome structure between S23321 and USDA110 showed strong colinearity; however, the symbiosis islands present in USDA110 were absent in S23321, whose genome lacked a chaperonin gene cluster (*groELS3*) for symbiosis regulation found in USDA110. A comparison of sequences around the tRNA-Val gene strongly suggested that S23321 contains an ancestral-type genome that precedes the acquisition of a symbiosis island by horizontal gene transfer. Although S23321 contains a *nif* (nitrogen fixation) gene cluster, the organization, homology, and phylogeny of the genes in this cluster were more similar to those of photosynthetic bradyrhizobia ORS278 and BTAi1 than to those on the symbiosis island of USDA110. In addition, we found genes encoding a complete photosynthetic system, many ABC transporters for amino acids and oligopeptides, two types (polar and lateral) of flagella, multiple respiratory chains, and a system for lignin monomer catabolism in the S23321 genome. These features suggest that S23321 is able to adapt to a wide range of environments, probably including low-nutrient conditions, with multiple survival strategies in soil and rhizosphere.

Oligotrophic bacteria are believed to be an important component of bacterial function and diversity in environments with low levels of nutrients (35). A series of experiments supervised by Hattori and his collaborators (17, 48, 50, 51) established methods to isolate oligotrophs by kinetic analysis of bacterial colony formation on agar plates, and to construct eco-collections based on this analysis. Although DNB (a 100-fold dilution of conventional nutrient broth agar) allows the isolation of both copiotrophic and oligotrophic bacteria, colonies of different species appear at different times, which can be modeled by using colony-forming curves (CFCs) (21). The incidence of oligotrophic bacteria relative to copiotrophic bacteria increases within the late CFC curves, CFC-III and CFC-IV (17).

Among the eco-collections of soil bacteria described above, *Bradyrhizobium* sp. S23321 belongs to the CFC-III group; it is an oligotrophic bacterium that was isolated from paddy soil at the experimental farm of Tohoku University (Kashimadai, Miyagi, Japan) in 1992 (17, 48). Phylogenetically, S23321 is very close to *Bradyrhizobium japonicum* USDA110, a symbiotic nitrogen-fixing soil bacterium that has the ability to form root nodules on soybean plants (7, 11, 31, 62). It is likely that *B. japonicum* USDA110 is also an oligotrophic bacterium because cell densities as high as 10^6^ CFU could be obtained in pure water (6).

Because soil bacteria that are phylogenetically close to *B. japonicum* often show non-symbiotic phenotypes for nodulation and nitrogen fixation even on compatible legume hosts (61), we predicted that S23321 was a non-nodulating bacterium that would not induce nitrogen-fixing nodules on legume roots. If so, genomic comparisons between S23321 and USDA110 would provide insights into the evolution of symbiotic associations with legumes. The genome sequence of USDA110 has already been published (31), facilitating a genome-wide comparison between these two species.

The genus *Bradyrhizobium* is part of the family *Bradyrhizobiaceae*, which belongs to the order *Rhizobiales* in the *Alphaproteobacteria*(20). In addition to having variations in symbiosis (24, 39, 60), various members of the *Bradyrhizobiaceae*, including *Bradyrhizobium*, harbor diverse biochemical functions such as photosynthesis, nitrification, sulfur oxidation, aromatic degradation, and oligotrophy (22, 25, 26, 33, 34, 45, 56, 68, 70). Thus, a long-standing question is how the members of the *Bradyrhizobiaceae* have acquired these diverse biochemical features.

To address some of the above questions, we determined the complete sequence of the *Bradyrhizobium* sp. S23321 genome and compared it with that of *B. japonicum* USDA110 and other sequenced members of the *Bradyrhizobiaceae* to identify genomic features relevant to symbiosis and versatile metabolic capabilities.

## Materials and Methods

### Bacterial strains and DNA preparation

*Bradyrhizobium* sp. S23321 (48) and *B. japonicum* USDA110 (31) were cultured for 7 d at 30°C in HM broth medium (5) containing 0.1% arabinose and 0.025% yeast extract. Cells of S23321 were harvested by centrifugation, and total DNA of S23321 was prepared by using a Blood Genomic DNA Extraction Maxiprep System (Viogene, Sunnyvale, CA, USA).

### Plant inoculation

Seeds of siratro (*Macroptilium atropurpureum* Urb. ‘Siratoro’; Snowbrand Seed, Sapporo, Japan) were surface-sterilized with 70% ethanol for 5 min and then with 3% hydrogen peroxide for 1 min. The seeds were then washed 10 times with sterile distilled water. The seeds were germinated in sterile vermiculite for 2 d at 25°C, and then transplanted into a Leonard jar (41, 72) containing sterile vermiculite and nitrogen-free nutrient solution (47). The seedlings were inoculated at 1×10^7^ cells per seed with either S23321 or USDA110. Plants were grown in a growth chamber (Biotron LH-300; Nippon Medical & Chemical Instrument, Tokyo, Japan) for 7 weeks at 25°C under a 16-h light/8-h dark cycle.

### Genome sequencing, assembly, and gap closing

The genome sequence of *Bradyrhizobium* sp. S23321 was determined by a whole-genome shotgun strategy using Sanger sequencing and 454 pyrosequencing. For Sanger sequencing using a 3730*xl* DNA Analyzer (Applied Biosystems, Foster City, CA, USA), about 20 μg DNA was sheared using a HYDROSHEAR (Gene Machine, San Carlos, CA, USA) for a short-insert genomic library, and another 80 μg was sheared for construction of a long-insert library. DNA fragments of 3 kb (for the short-insert library) and 10 kb (for the long-insert library) were subcloned into the plasmid vector pTS1 (Nippon Gene, Tokyo, Japan) to construct shotgun libraries. Template DNA was prepared by amplifying insert DNA of each clone using PCR of an aliquot of the bacterial culture. We generated 53,760 reads by sequencing both ends of the clones, giving 4.5-fold genome coverage. For pyrosequencing using a GS FLX Titanium system (Roche Applied Science, Mannheim, Germany), 5 μg of the genomic DNA was sheared using nebulization to obtain fragments ranging from 300 to 800 bp. Template DNA was prepared according to the supplier’s protocol. The pyro-sequencing data were assembled using Newbler assembly software, generating 200 contigs. The GS FLX contig sequence data were then imported as “pseudoreads” of the Sanger data into the Phred/Phrap/Consed system (8, 9, 16). The hybrid assembly of the Sanger and 454 pyrosequencing data eventually generated 16 contigs. Gap closing and resequencing of low-quality regions in the assembled data were performed by PCR, primer walking, and direct sequencing of appropriate plasmid clones. The finished sequence was estimated to have an error rate of less than one per 10,000 bases (Phrap score of ≥40) by Phrap software.

### Gene assignments and annotations

Genes for structural RNAs were identified by similarity searches against an in-house structural RNA database that had been constructed from data available in GenBank. rRNAs were predicted on the basis of similarity searches against those of *B. japonicum* USDA110 using the BLASTN program. tRNAs, tmRNAs, and non-coding RNAs were predicted using the non-coding RNA sequence database fRNAdb version 3.4 (http://www.ncrna.org/frnadb/) and Rfam version 10.0 (http://rfam.sanger.ac.uk/) (13). Protein-encoding regions were predicted using the MetaGeneAnnotator with default parameters (53). A circular genome map showing the GC skew and the GC content was created using the GCview Server with the default parameters (19). Putative orthologous genes were identified by using bi-directional BLASTN comparisons with an E-value cutoff of 10^−20^ among three *Bradyrhizobium* strains: S23321, USDA110, and ORS278. Orthologous relationships were depicted in a Venn diagram. Community annotation was adopted in the present study as described in the Supplementary materials.

### Phylogenetic analysis

Phylogenetic analysis was performed by comparing the 16S rRNA gene sequence (genome coordinates 6,627,526–6,626,243 bp), the internal transcribed spacer (ITS) sequence between the 16S and 23S rRNA genes (coordinates 6,626,088–6,625,301 bp), and the *nifH* genes of S23321 to those of other *Bradyrhizobiaceae* (Table S1). The sequences were aligned using the CLUSTAL W program (71). Neighbor-joining trees were constructed using MEGA version 5.02 (69), and 1,000 bootstrap replicates were used to generate a consensus tree.

### Similarity search between S23321 and other members of the *Bradyrhizobiaceae*

The genome sequences of S23321 and USDA110 were compared by using the programs MUMmer (38) and GenomeMatcher (57) at the nucleotide level. The GC content was calculated by using IMC (*i**n silico*Molecular Cloning; in silico biology; http://www.insilicobiology.co.jp/indexEN.html). The annotated genome sequence of USDA110 was obtained from RhizoBase (http://genome.kazusa.or.jp/rhizobase). Homology searches for open reading frames (ORFs) in the genome of S23321 were performed against the gene database of the USDA110, other members of the *Bradyrhizobiaceae*, and the NCBI nr (non-redundant protein sequences) database (59) using the BLASTP program with an E-value cutoff of ≥10^−10^ unless otherwise indicated.

### Electron microscopy

Cells of S23321 were grown at 30°C in arabinose–gluconate medium for 2 d (28). The cells were harvested by centrifugation and then suspended in 10 mM phosphate buffer. An aliquot of the suspension was directly applied to an EM grid and negatively stained with 2% phosphotungstic acid (pH 7). Samples were observed with a JEM-1200EXII electron microscope (JEOL, Tokyo, Japan). Micrographs were taken at an accelerating voltage of 80 kV.

### Nucleotide sequence accession number and culture deposition

The complete nucleotide sequence of *Bradyrhizobium* sp. S23321 was submitted to DDBJ under accession number AP012279. *Bradyrhizobium* sp. strain S23321 was deposited in the Japan Collection of Microorganisms (JCM) as JCM 18004.

## Results

### Nodulation

When surface-sterilized seeds of siratro were inoculated with S23321, no root nodules formed (Fig. S1). On the other hand, USDA110 induced root nodules (44 nodules plant^−1^ on average) under the same condition (Fig. S1). Siratro is known to host a broad range of rhizobia, and it is usually nodulated when inoculated with *Bradyrhizobium* sp. isolated from various legumes (27, 61). Generally, rhizobial *nodABC* genes and the lipochito-oligosaccharidic Nod factor are required for molecular recognition between rhizobia and legumes; although nodulation of some legumes such as *Aeschynomene indica* occurs in the absence of *nodABC* genes and Nod factor, it is considered an unusual phenomenon (15). It was thus concluded that S23321 is not able to form nodules, at least not in a Nod factor-dependent manner.

### Phylogeny

**Fig. 1:**
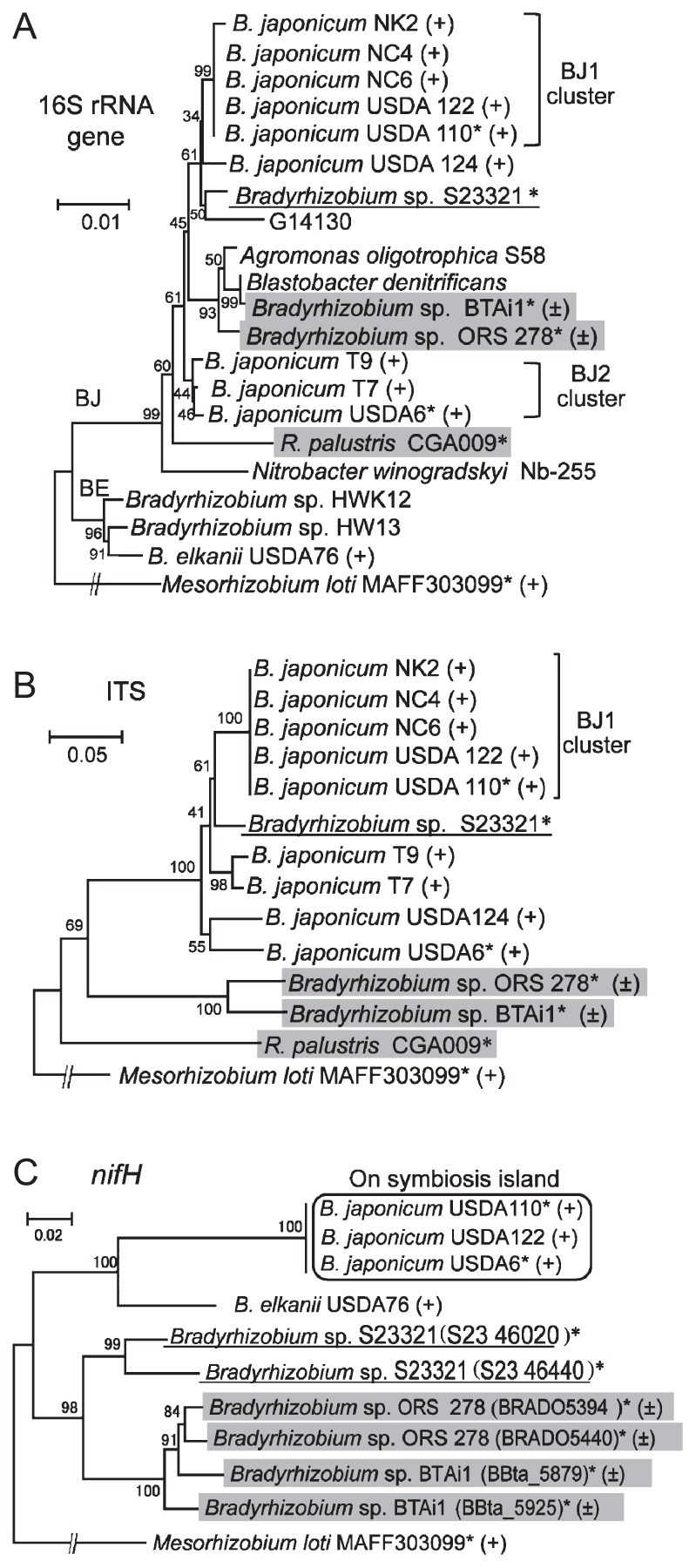
Phylogenetic relationships of *Bradyrhizobium* sp. S23321 and other members of the *Bradyrhizobiaceae* (Table S1) based on 16S rRNA gene sequences (A), internal transcribed spacer (ITS) sequences (B), and *nifH* gene sequences (C). For all trees, *Mesorhizobium loti* MAFF303099 was used as an outgroup. Numbers at the nodes are the percentage of 1,000 bootstrap replications supporting that partition. Branches corresponding to partitions reproduced in less than 50% of the bootstrap replicates are collapsed. BJ and BE are major clusters including *B. japonicum* and *B. elkanii*, respectively. The BJ1 and BJ2 clusters of *B. japonicum* were defined based on phylogenetic trees of 16S rRNA genes and ITS sequences as described previously (26). Strains capable of Nod factor-dependent and -independent nodulation (15) are marked with (+) and (±), respectively. Photosynthetic strains are shaded in gray. Asterisks show the strains of bradyrhizobia for which the complete genome sequence is available. S23321 is underlined. In the phylogenic trees based on 16S rRNA gene sequences (A) and ITS sequences (B), S23321 was clustered with *B. japonicum*. In the *nifH* tree (C), S23321 was closer to *Bradyrhizobium* sp. BTAi1 and ORS278 than to the *nif* genes on the symbiosis islands of *B. japonicum* (rectangle in panel C).

To examine the phylogenetic relationships between *Bradyrhizobium* sp. S23321 and other members of the *Bradyrhizobiaceae*, a phylogenetic tree was constructed based on 16S rRNA sequences (Fig. 1A). The *Bradyrhizobiaceae* strains were divided into two large groups (BJ and BE in Fig. 1A). The BJ group comprised 17 strains including S23321, *B. japonicum* (BJ1 and BJ2 clusters) (26), and photosynthetic bradyrhizobia (ORS278 and BTAi1); the BE group comprised *B. elkanii* USDA76 and *Bradyrhizobium* sp. HWK12 and HW13. In the BJ group, S23321 was clustered with BJ1 strains including USDA110. This result shows that S23321 is phylogenetically very close to *B. japonicum* USDA110 based on the sequences of the 16S rRNA genes (Fig. 1A). To increase the phylogenetic resolution, the ITS region was also analyzed (Fig 1B). S23321 was again clustered within a group of Nod factor–dependent nodulating strains (denoted by “+” in Fig 1B) of *B. japonicum*, and fell outside of the photosynthetic clade (Fig. 1B) (15).

### General genome description

**Fig. 2:**
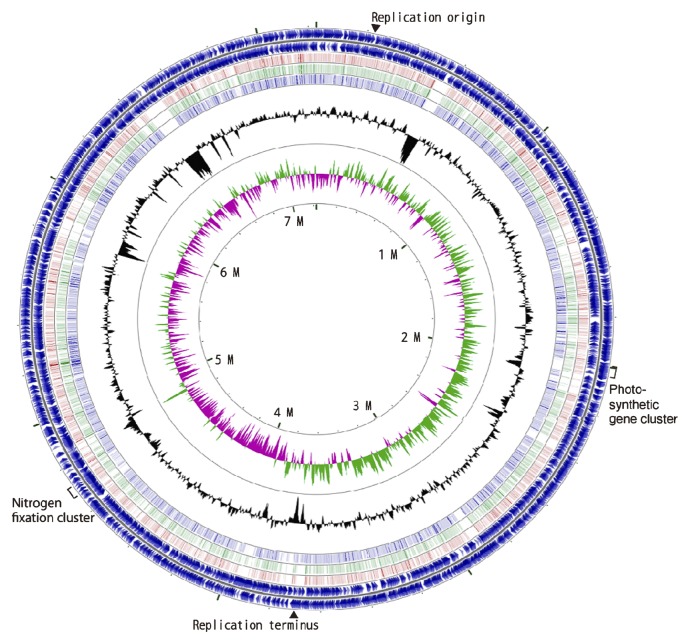
Circular representation of the chromosome of *Bradyrhizobium* sp. S23321. The outermost circle and the second circle show the positions of the putative protein-encoding genes in clockwise and counterclockwise directions, respectively. The third, fourth, and fifth circles from the outside represent BLASTN comparisons with *Bradyrhizobium* sp. BTAi1, *Bradyrhizobium* sp. ORS278, and *B. japonicum* USDA110, respectively (E-value <10^−10^). The innermost and second-innermost circles show the GC skew and the GC content, respectively. The GC content circle shows the deviation from the average GC content of the entire sequence (higher than average GC content in green, lower than average in purple). The markings inside the innermost circle represent genome positions in Mb. The positions of the putative replication origin, putative replication terminus, nitrogen fixation genes, and photosynthetic genes are shown outside of the outermost circle.

The genome of *Bradyrhizobium* sp. S23321 is a circular chromosome of 7,231,841 bp with an average GC content of 64.3%. Several low-GC regions were also found in the genome, suggesting the presence of horizontally acquired regions (Fig. 2). Based on BLASTN comparison with the genomes of BTAi1, ORS278, and USDA110 (the third, fourth, and fifth circles from the outside in Fig. 2), at least two large low-GC regions 526–582 kb (56.4% GC) and 6,450–6,535 kb (56.7% GC) were unique to S23321 (Fig. 2).

The putative replication origin and terminus were predicted by analyses of GC skew and signature sequences (Fig. 2 and Supplementary materials). The S23321 genome contained one copy of the rRNA gene cluster, in the order 16S-*trnI*-*trnA*-23S-5S, at coordinates 6,622,191–6,627,579 bp. Forty-five tRNA genes, which corresponded to all 20 of the standard amino acids, were scattered throughout the S23321 genome. The S23321 genome also contained a two-piece tmRNA gene similar to that in the USDA110 genome (31, 32).

In total, 6,898 ORFs were predicted using MetaGeneAnnotator (53). The functions of predicted protein-coding genes were manually annotated through comparisons with the NCBI nr and Swiss-Prot databases by a jamboree consortium as described in the Supplementary materials.

### Structural features of the S23321 genome

**Fig. 3:**
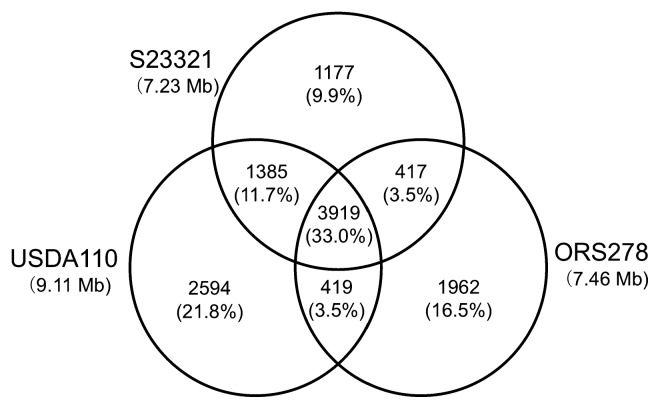
Comparative genomics analysis among *Bradyrhizobium* strains S23321, USDA110, and ORS278. Each genome is represented by a circle, and the numbers of shared and unique genes are shown by overlapping and non-overlapping parts of the circles. The proportion of total genes represented by each area of the diagram is shown in parentheses.

In order to compare the gene contents among related strains, a BiBlast comparison was conducted among bradyrhizobial strains S23321, USDA110, and ORS278 (Fig. 3). The comparison revealed 3,919 genes that were conserved among all three strains; this is 33.0% of the combined non-redundant set of genes (11,873) (Fig. 3). The number of genes unique to S23321 (1,177 [9.9%]) was lower than that for USDA110 (2,594 [21.8%]) and ORS278 (1,962 [16.5%]). S23321 and USDA110 shared 1,385 genes (11.7%) not found in ORS278. This number is markedly larger than the 417 (3.5%) shared only between S23321 and ORS278, and the 419 (3.5%) shared only between USDA110 and ORS278. These results indicate that, among the three strains, S23321 and USDA110 are the most closely related in terms of gene content. This conclusion is consistent with the results of the phylogenetic analysis based on the 16S rRNA gene (Fig. 1A).

**Fig. 4:**
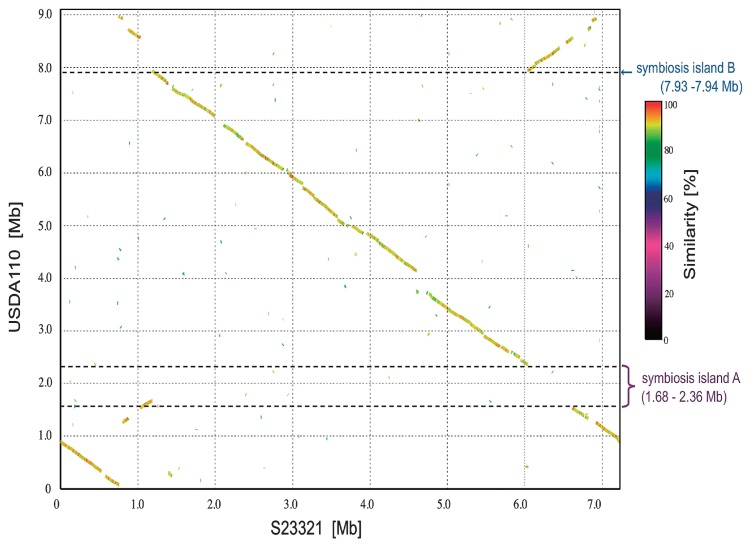
Comparison of the genome sequences of S23321 and USDA110. The nucleotide sequence of *Bradyrhizobium* sp. S23321 was compared with that of *B. japonicum* USDA110 by MUMmer and is represented by a syntenic dot plot of S23321 (*x*-axis) versus USDA110 (*y*-axis). Dot color indicates % similarity, as indicated by the key to the right of the graph.

**Fig. 5:**
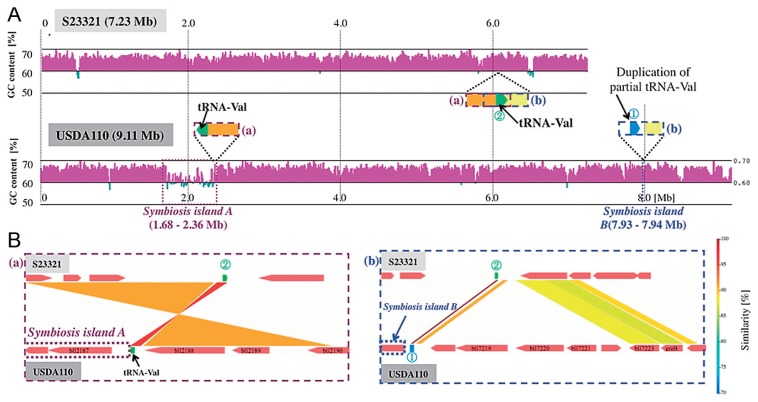
Sequence analysis of the borders of the symbiosis islands and tRNA-Val genes in the genomes of *Bradyrhizobium* sp. S23321 and *B. japonicum* USDA110. (A) GC contents of the genome sequences of S23321 and USDA110 along with the positions of symbiosis islands and tRNA-Val gene sequences. GC contents were calculated by using a sliding-window size of 5 kb with a step size of 100 bp. The GC contents of the windows are shown with magenta (≥60% GC) and green (<60% GC) bars. In the map of USDA110, symbiosis island A is delineated by dotted purple lines, and symbiosis island B by dotted blue lines. (B) Comparison of sequence synteny around the boundaries of symbiosis islands A and B using GenomeMatcher.

One of the differences between the biological features of S23321 and USDA110 is that USDA110 is able to form root nodules whereas S23321 is not. The comparison of the genome sequences between S23321 and USDA110 by using MUMmer (38) indicated no distinct symbiosis island (a large cluster of symbiosis genes, characterized by low GC content) in the genome of S23321 (Fig. 4). In USDA110, two partial symbiosis islands were previously identified within the chromosome (31). The GC content of symbiosis island A, which includes most of the symbiosis genes in USDA110, was lower than that of other regions of the genome (Fig. 5A). Kaneko *et al.*(31) found that the smaller symbiosis island fragment (B) in USDA110 was adjacent to a partial duplication of the tRNA-Val gene (Fig. 5A), suggesting a target region that had been duplicated upon insertion. A BLASTN search for tRNA-Val(CAC) sequences in the S23321 genome revealed a single copy of tRNA-Val (75 bp) that was identical in sequence to that in USDA110, but the partial tRNA-Val gene (45 bp) was not found in S23321 (Fig. 5A). Thus, no symbiosis island is present in the S23321 genome.

When we performed detailed comparisons of the border regions adjacent to the tRNA-Val gene sequences in the genomes of S23321 and USDA110 (Fig. S2), homologous nucleotide sequences (Fig. S2A and B) were found adjacent to tRNA-Val (75 bp) and to the partial duplication of tRNA-Val (45 bp) in the genomes of S23321 and USDA110 (Fig. 5A and B). In addition, the orientations of the two nucleotide sequences (yellow and orange regions in Fig. 5A and B) were reversed in the S23321 genome relative to the USDA110 genome. The genomic positions of the two sequences corresponded to the borders of symbiosis islands A and B (2.36 and 7.93 Mb) in the USDA110 genome (Fig. 5A and B), and strong colinearity was observed between the sequences (2.36–7.93 Mb in the USDA110 genome; 1.3–6.1 Mb in the S23321 genome) (Fig. 4), suggesting a large inversion around tRNA-Val in the USDA110 genome relative to S23321. Genome rearrangement, including symbiosis island insertion, translocation, and inversion, probably occurred on the USDA110 genome.

Kaneko *et al.*(29) recently reported the genome structure of *B. japonicum* USDA6 and found a novel symbiosis island (Locus C) that is highly conserved between the genomes of *B. japonicum* USDA110 (coordinates 8,974,971–0–70,365 bp) and USDA6 (coordinates 9,113,996–0–70,356 bp) (29); symbiosis islands A and B are also conserved between the two *B. japonicum* species. Genomic comparison of S23321 with USDA110 and USDA6 showed that S23321 completely lacks Locus C (Fig. S3). This result supports the idea that S23321 contains an ancestral-type genome that precedes the insertion of a symbiosis island (Fig. 4 and 5). In addition, it appears that complicated genome rearrangements may have occurred in the genomes of *B. japonicum* USDA110 and USDA6 following the insertion of a symbiosis island.

### Nitrogen fixation

**Fig. 6:**
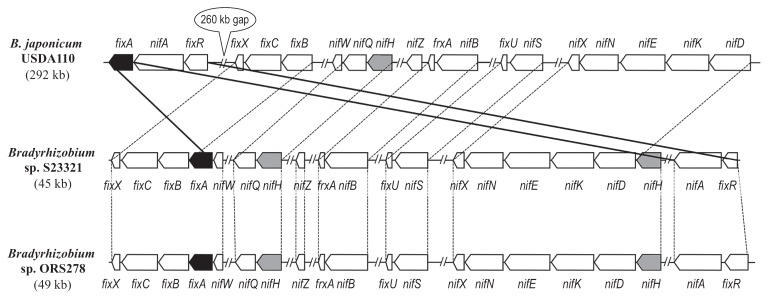
Conserved clusters of genes related to nitrogen fixation in *Bradyrhizobium japonicum* USDA110, *Bradyrhizobium* sp. S23321, and *Bradyrhizobium* sp. ORS278. Values in parentheses indicate the length of the *nif* gene region within each genome. Double slash marks represent chromosome regions that are not shown. Nitrogen fixation-related genes in clusters that are conserved among the three strains are shown with white arrows. *fixA*, which differs in location among the strains, is shown with black arrows. *nifH*, which varies in copy number, is shown with shaded arrows.

In S23321, 20 ORFs related to nitrogen fixation are located in a 45-kb region (coordinates 4,682,626–4,727,512 bp) (Fig. 6). *fixA* (S23_45960) is located immediately upstream of the *fixBCX* cluster (S23_45950–45930), and the other genes for nitrogen fixation (*nifDKENX*, *nifH*, and *nifA*) are located upstream of the *fixABCX* gene cluster (S23_45960–45930) (Fig. 6). The order of the *fixABCX* gene cluster in S23321 is the same as those in the genomes of several other rhizobia (*e.g.*, *Mesorhizobium loti* MAFF303099, *Sinorhizobium meliloti* 1021, *Rhizobium etli* CFN42, and *Rhizobium* sp. NGR234); however, the arrangement of the *fixABCX* gene cluster of S23321 is different from that in USDA110. In USDA110, *fixA* (blr2038) is located downstream of *fixR* (blr2036) and *nifA* (blr2037), and the remaining *fixBCX* gene cluster (blr1773–1775) is located 260 kb upstream of *fixR-nifA-fixA* (Fig. 6). Furthermore, two *nifH* genes (S23_46020, S23_46440) were identified in S23321 upstream of *nifQ* (S23_46010) and *nifD* (S23_46430), which is similar to the arrangement in ORS278. In contrast, USDA110 only has a single *nifH* gene upstream of *nifQ* (Fig. 6).

When *nifH* gene sequences were compared among bradyrhizobial strains (Fig. 1C), the two copies of the *nifH* gene found in S23321 resembled the situation in *Bradyrhizobium* sp. ORS278 and BTAi1, rather than the single *nifH* gene on the symbiosis island of *B. japonicum* strains including USDA110. These results strongly suggested that the evolutionary history of nitrogen fixation genes is different between those in the S23321 genome and those on the symbiosis islands of *B. japonicum*.

### Photosynthesis gene cluster

**Fig. 7:**
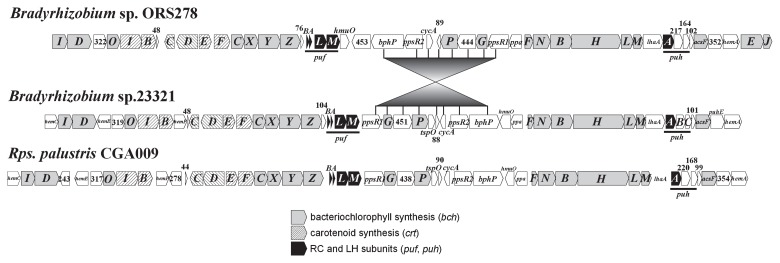
Comparison of photosynthetic gene clusters among *Bradyrhizobium* sp. S23321, *Bradyrhizobium* sp. ORS278, and *Rhodopseudomonas* palustris CGH0009.

S23321 has a photosynthesis gene cluster (PGC), although no PGC was found in the genome of USDA110 (Fig. 7). The cluster in S23321 contains two well-conserved superoperonal gene arrangements (subclusters), *crtEF-bchCXYZ-pufBALM* and *bchFNBHLM-lhaA-puhA*, which contain genes encoding the reaction center (*puf*) and the core light-harvesting subunits (*puh*), respectively (23). Both subclusters in S23321 are located on the same DNA strand, as are those in the closely related species *Rhodopseudomonas palustris* CGA009 (49) and in *Bradyrhizobium* sp. BTAi1 and ORS278 (15). Notably, the gene arrangement in the PGC of S23321 is nearly identical to that in *R. palustris*, in which the genes coding photosynthesis repressor proteins (*ppsR1* and *ppsR2*) and bacteriophytochrome (*bphP*) are flanked by the two conserved subclusters. In addition, genes *hemA*, *hemC*, *hemE*, and *hemF*, which are required for the synthesis of protoporphyrin IX, the consensus precursor for hemes and bacteriochlorophyll biosyntheses, are found in the PGCs of S23321 and *R. palustris* CGH009 in identical locations. The gene arrangement in the PGC of ORS278 was nearly identical to that of BTAi1 (data not shown), but different from those of S23321 and *R. palustris* as follows: (i) inversion of a cluster containing *ppsR1*, *bphP*, and the genes in between, (ii) the lack of *hem* genes (except for *hemA*), and (iii) the presence of two bacteriochlorophyll synthesis genes, *bchE* and *bchJ*. The closer relationship between the PGC regions in S23321 and the phototrophic species *R. palustris* than to those in other *Bradyrhizobium* species is not consistent with the phylogenetic relationship based on 16S rRNA gene sequences. All of the *bch* genes of S23321 except for *bchG* and *bchX* showed the highest sequence identities to their orthologues in *R. palustris*. A phylogenetic tree based on BchH sequences (Fig. S4) clearly showed that S23321 was clustered with *R. palustris* rather than with *Bradyrhizobium* spp. BTAi1 and ORS278. Such inconsistencies might be explained by the transfer of the PGC (23) from a phototrophic species, *e.g.*, from an ancestor or relative of *R. palustris*, to S23321, although more sequence data and careful analyses will be required to trace the evolution of this region.

Based on the gene arrangement analysis, S23321 would be expected to synthesize the simplest type of photosynthetic apparatus, one in which the reaction center (RC) complex consists of membrane-bound L and M subunits and a periplasmic H subunit but does not contain a cytoplasmic tetraheme cytochrome subunit. The light-harvesting system would also be simple, including only a core antenna complex surrounding the RC; this arrangement is called the light-harvesting 1 (LH1) complex. Similar photosynthetic apparatuses have also been reported in *Bradyrhizobium* sp. BTAi1 and ORS278 (14). The conserved photosynthetic mechanism of S23321 would also be advantageous in nutrient-poor habitats.

### Flagella

**Fig. 8:**
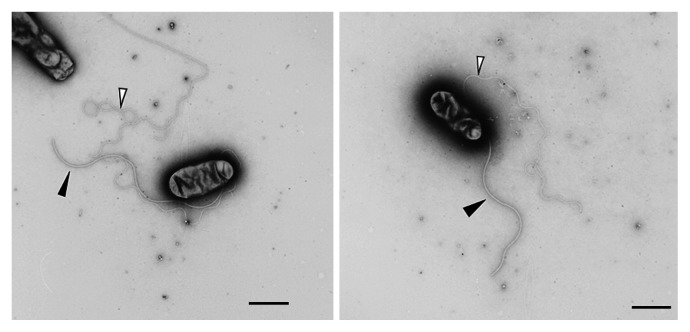
Electron micrographs of negatively stained cells of *Bradyrhizobium* sp. S23321. Black and white arrowheads indicate polar and lateral flagella, respectively. Bars represent 1 μm.

The flagellar genes of S23321 are similar to those of *B. japonicum* in terms of gene sequences and gene order, except that the directions of transcription are different. Most of the flagellar genes are compactly clustered in two regions: the first located at 1,378 kb and the second at 2,536 kb (Fig. S5). From the genetic and biochemical characteristics of the two types of flagella in *B. japonicum*, we assume that the genes in the first region might be used for the biosynthesis and function of the lateral flagella and those in the second for the polar flagellum (1). There is another cluster of eight genes at 1,592 kb that might be used for the polar flagellum, judging from the fact that these genes are missing from the second cluster. As expected from the genome analysis, cells of this strain have both polar and lateral flagella (Fig. 8). The photosynthetic bradyrhizobia ORS278 and BTAi1 contain the genes for polar flagella, but appear to lack most genes for lateral flagella (Table S2).

There are six genes in S23321 annotated for flagellin, tentatively named *fliC1* to *fliC6*. The N-terminal amino acid sequences of FliC5 and FliC6 are identical to those of the *Lactobacillus crispatus* S-layer proteins SlpA and SlpB (64). The polar flagellum is wrapped with a membranous sheath, which might consist of S-layer proteins. There are three clusters of chemotaxis genes in the S23321 genome, suggesting motility toward many attractants (Fig. S5 and Supplementary materials).

### Lack of genes for symbiosis in S23321

To survey the nodulation-related genes in the S23321 genome, all of the putative gene products of S23321 were compared with the 655 gene products located on the symbiosis islands in USDA110 by using BLASTP analysis. Although 61 of the 6,898 genes in S23321 were conserved in the symbiosis islands of USDA110, none of them were nodulation genes (*nodDYABCSUIJ*), which are found in USDA110 and most other rhizobia (Table S3) (12, 30, 63).

Stress responses are highly interconnected with symbiotic associations (49). S23321 is similar to BTAi1 and ORS278 in the number of stress-response genes (Table S4). Lund (42) reported that more than 80% of the *Alphaproteobacteria* species have one or two copies of chaperonin homologues, and suggested that most root-nodulating and nitrogen-fixing bacteria have more than three copies of *groEL* homologues. *B. japonicum* USDA110 has seven copies of *groEL*, which to our knowledge is the highest number in the domain *Bacteria*. S23321 possesses three sets of *groEL*–*groES* genes, but lacks *groEL3–groES3*, which is transcribed from a σ^54^-dependent promoter under the control of NifA, a transcriptional regulator of symbiotic nitrogen fixation in *B. japonicum* USDA110 (10). This suggests that the symbiotic bacterium USDA110 acquired the *groEL3–groES3* genes during its evolution.

### Terminal oxidase

There were a number of differences between S23321 and USDA110 in the genes for terminal oxidase. Six terminal oxidase complexes were found in the S23321 genome: three of these six were cytochrome *c* oxidases, whereas the other three were quinol oxidases (Table S5). The members of these six oxidase complexes shared a high degree of amino acid sequence identity with *B. japonicum* USDA110. On the other hand, no operon closely related to the *cydAB*-like genes of USDA110 (blr3728–3729) (18) was found in S23321 (4). USDA110 harbors eight terminal oxidase complexes (18), giving it one of the most highly branched respiratory chains of all aerobic prokaryotes. Of the six terminal oxidase complexes in S23321, the S23_52330-52360 operon most likely corresponds to the *fixNOQP* gene cluster in USDA110, which encodes an oxidase with an extremely high affinity for O_2_ that is expressed microaerobically, *i.e.*, under extremely low oxygen levels (43, 54, 55, 58). The S23_52770–52800 operon is most closely related to the *coxWXYZ* gene cluster, which is also microaerobically expressed and encodes a *bb*_3_-type ubiquinol oxidase (67). The S23_06500–06530 and S23_07900–07930 operons are closely related to *cyoABCD* and *coxABCD*, respectively, which are expressed under aerobic conditions.

Low oxygen concentrations induce *fixNOQP* expression in USDA110 (18). It has been reported that the *fixNOQP* operon and denitrification genes are regulated by the FixLJ two-component regulatory system and FixK_2_(2, 46, 52). S23_52400, 52410, and 52430 in S23321 are closely related to *fixL*, *fixJ*, and *fixK*_2_ in USDA110, respectively (amino acid sequence identity of 85.0%, 85.9%, and 89.7%, respectively). The presence of these terminal oxidase genes in S23321 suggests that S23321 is capable of functioning throughout a wide range of oxygen stress.

### Transporters

The S23321 genome contains 492 ORFs for ABC (ATP-binding cassette) proteins and their interacting partners. *B. japonicum* USDA110 possesses 382 orthologues of these 492 ORFs (Table S6). The ABC systems of S23321 and USDA110 are rich in genes encoding HAA (hydrophobic amino acids and amides) and OPN (oligopeptides and nickel) family transporters as compared with those of *Pseudomonas putida* KT24440 and *Streptomyces coelicolor* A3 (2, 3) (Table S7 and Supplementary materials).

### Other biochemical features

Soil bacteria usually compete against each other for nutrients, including aromatic compounds, to help them survive (40, 65, 67). S23321 has genes for the degradation of toluene and 4-chlorocatechol as well as for catabolism of vanillate (lignin monomer) and formaldehyde (C1 compound), which are also found in *B. japonicum* USDA110 (66) (Figs. S6, S7, and S8).

*Bradyrhizobium* cells often show chemoautotrophic growth (45). The genome survey suggested that S23321 should be able to grow chemoautotrophically using CO and CO_2_ as an electron donor and a carbon source, respectively, because of the presence of *cox* and *cbb* genes (Fig. S9 and Supplementary materials). Although the S23321 genome possesses many genes for carbon metabolic pathways such as glycolysis, gluconeogenesis, the citrate cycle, the pentose phosphate pathway, and the glyoxylate cycle (Table S8), it apparently lacks several genes for nitrogen metabolism such as those for N_2_O reductase and glutamate dehydrogenase (Table S9).

## Discussion

Symbiotic nitrogen fixation is a vital component of the global nitrogen cycle and of agricultural practices worldwide (37, 44). The establishment of rhizobia–legume symbiosis is assumed to have required the evolution of novel developmental and functional programs (36). Analysis of the genome sequence of S23321 provides an insight into important biological and evolutionary aspects of symbiotic nitrogen fixation.

*B. japonicum* USDA110 is able to form root nodules on legumes and establish symbiotic nitrogen fixation (31). Most of the genes for symbiotic nitrogen fixation in USDA110 are located on symbiosis islands (31). S23321 is the closest known strain to *B. japonicum* that does not have a symbiosis island. (Fig. 1A and B). Comparative analysis strongly suggested that the S23321 genome is an ancestral type that precedes symbiosis island acquisition, based on the signature sequences adjacent to the tRNA-Val gene (Fig. 4) and the lack of nodulation genes. In addition, the lack of chaperonin cluster *groELS3*, which is involved in symbiosis, supports this idea. On the other hand, the presence of *fixNOPQ* and six different respiratory chains in the S23321 genome suggested that the terminal oxidase (a cytochrome *bc*_1_ complex that functions under low oxygen stress) is not part of symbiotic nitrogen fixation, but instead provides broad adaptation to a wide range of oxygen stress in the environment.

Although the S23321 genome contains several *nif* (nitrogen fixation) gene clusters, their organization, homology, and phylogeny are more similar to those of the photosynthetic bradyrhizobia species ORS278 and BTAi1 than to those on the symbiosis island of USDA110 (Fig. 1C and Fig. 6). Interestingly, the S23321 genome contains a full set of photosynthetic genes that are not found in USDA110 at all, but which are highly similar to those of *R. palustris* in terms of gene organization and homology (Fig. 7). On the other hand, in phylogenetic analyses based on 16S rRNA and ITS sequences (Fig. 1A and B), S23321 fell into the *B. japonicum* cluster, far from the photosynthetic bradyrhizobia ORS278 and BTAi1 and from *R. palustris*. Regarding this apparent discrepancy, there are at least two possible explanations. One is that non-nodulating bradyrhizobia such as S23321 might have originally carried prototype photosynthetic and *nif* gene clusters that were similar to those of *Bradyrhizobium* spp. ORS278 and BTAi1 and *R. palustris*. After symbiosis island insertion, these gene clusters were deleted from the genome. Another possibility is that the S23321 genome might have acquired photosynthetic or *nif* gene clusters via horizontal gene transfer from a different lineage of bacteria.

Finally, we want to emphasize that *Bradyrhizobium* sp. S23321 could be a model microorganism for non-nodulating soil bradyrhizobia, providing a valuable tool for experiments on the genetics, physiology, and ecology of such species. For example, because S23321 lacks a symbiosis island, it may be used as a recipient in symbiosis island transfer to study the symbiotic evolution of bradyrhizobia.

## Supplementary materials
