# Supplementary material for: Complete Genome Sequence of Bradyrhizobium sp. S23321: Insights into Symbiosis Evolution in Soil Oligotrophs
Source: Microbes Environ. 2012 Mar 28;27(3):306–15. doi: 10.1264/jsme2.ME11321 (PMC4036050; doi:10.1264/jsme2.ME11321)
Supplement: Supplementary file 1 [file 27_306_s1.pdf]

Supplementary materials

Complete Genome Sequence of *Bradyrhizobium* sp. S23321: Insights into Symbiosis Evolution in Soil Oligotrophs

Takashi Okubo[1*], Takahiro Tsukui[1*], Hiroko Maita[1,2*], Shinobu Okamoto[3*], Kenshiro Oshima[4], Takatomo Fujisawa[5], Akihiro Saito[6], Hiroyuki Futamata[7], Reiko Hattori[8], Yumi Shimomura[9], Shin Haruta[10], Sho Morimoto[9], Yong Wang[9], Yoriko Sakai[9], Masahira Hattori[4], Shin-Ichi Aizawa[11], Kenji V. P. Nagashima[10], Sachiko Masuda[1], Tsutomu Hattori[8], Akifumi Yamashita[1], Zhihua Bao[1], Masahito Hayatsu[9], Hiromi Kajiya-Kanegae[3], Ikuo Yoshinaga[12], Kazunori Sakamoto[13], Koki Toyota[14], Mitsuteru Nakao[3], Mitsuyo Kohara[2], Mizue Anda[1], Rieko Niwa [9], Park, Jung-Hwan[12], Reiko Sameshima-Saito[15], Shin-ichi Tokuda[16], Sumiko Yamamoto[5], Syuji Yamamoto[7], Tadashi Yokoyama[17], Tomoko Akutsu[2], Yasukazu Nakamura[5], Yuka Nakahira-Yanaka[18], Yuko Takada Hoshino[9], Hideki Hirakawa[2], Hisayuki Mitsui[1], Kimihiro Terasawa[1], Manabu Itakura[1], Shusei Sato[1,2], Wakako Ikeda-Ohtsubo[1], Natsuko Sakakura[5], Eli Kaminuma[5], and Kiwamu Minamisawa[1**]

[1]*Graduate School of Life Sciences, Tohoku University, 2-1-1 Katahira, Aoba-ku, Sendai, Miyagi 980-8577, Japan*; [2]*Laboratory for Plant Genome Informatics, Kazusa DNA Research Institute, 2-6-7 Kazusakamatari, Kisarazu, Chiba 292-0818, Japan*; [3]*Database Center for Life Science (DBCLS), Research Organization of Information and Systems (ROIS), 2-11-16 Yayoi, Bunkyo-ku, Tokyo 113-0032, Japan*; [4]*Graduate School of Frontier Sciences, University of Tokyo, 5-1-5, Kashiwa-no-ha, Kashiwa, Chiba 277-8561, Japan*; [5]*Center for Information Biology and DNA Data Bank of Japan, National Institute of Genetics, Research Organization for Information and Systems, Yata, Mishima, Shizuoka 411-85, Japan*; [6]*Department of Material and Life Science, Faculty of Science and Technology, Shizuoka Institute of Science and Technology 2200-2 Toyosawa, Fukuroi, Shizuoka 437-8555, Japan*; [7]*Department of Material Science and Chemical Engineering, Shizuoka University, 3-5-1 Jyohoku, Naka-ku, Hamamatsu, Shizuoka, 432-8561, Japan*; [8]*Attic Lab, 1-6-2-401 Komegafukuro, Aobaku, Sendai, Miyagi 980-0813, Japan*; [9] *National Institute for Agro-Environmental Sciences, 3-1-3 Kannondai, Tsukuba, Ibaraki 305-8604, Japan*; [10]*Graduate School of Science and Engineering, Tokyo Metropolitan University, 1-1 Minami-Osawa, Hachioji-shi, Tokyo 192-0397, Japan*; [11]*Department of Life Sciences, Prefectural University of Hiroshima, 562 Nanatsuka, Shobara, Hiroshima 727-0023, Japan*; [12]*Graduate School of Agriculture, Kyoto University, Oiwake-cho, Kitashirakawa, Sakyo-ku, Kyoto 606-8502, Japan*; [13]*Graduate School of Horticulture, Chiba University, 648 Matsudo, Matsudo, Chiba 271-8510, Japan*; [14]*Tokyo University of Agriculture and Technology, 2-24-16, Naka, Koganei, Tokyo 184-8588, Japan*; [15]*Faculty of Agriculture, Shizuoka University, 836 Ohya, Suruga-ku, Shizuoka 422-8529, Japan*; [16]*National Institute of Vegetable and Tea Sciences, National Agriculture and Food Research Organization, 3-1-1 Kannondai, Tsukuba, Ibaraki 305-8666, Japan*; [17]*Institute of Agriculture, Tokyo university of Agriculture and Technology, 3-5-8 Saiwaicho, Fuchu, Tokyo 183-8509, Japan*; and [18]*Graduate School of Life and Environment Sciences, University of Tsukuba, 1-1-1 Ten-noudai, Tsukuba, Ibaraki 305-8572, Japan*

Materials and Methods

Community annotation

The accuracy of genome annotation was increased by effectively combining automated annotation and community-based manual annotation. To avoid the problems of information duplication and inconsistency that can occur in community annotation work, we used a communication system that made use of a Wiki (user-editable website) and mailing-list system, created standard operating procedures (SOPs), and maintained central control of annotation data. KazusaAnnotation (http://genome.kazusa.or.jp/rhizobase/), an annotation support tool, was used to avoid duplication of annotation work. The Kazusa DNA Research Institute supplied an annotation database (cgaRhizoBase) so that a list of all submissions by participating users could be viewed. SOPs were developed to maintain consistency in the annotation work. All cooperative annotation work complied with these procedures. To maintain consistency in the style of annotation data, all submissions of annotation information were made through the KazusaAnnotation entry support tool.

Standard operating procedures (SOPs)

SOP documents were developed detailing the work procedures. These were shared with the community using the Wiki system. The SOPs included methods for using these tools and information, methods for proper use and attribution when making use of annotations from various public databases, a basic list of terminology for genome annotation, *etc*. The objective of the SOPs was to bring together background knowledge for the participants. Initially, a gene set created by automated annotation was provided as a starting point for the manual annotation. The manual annotation work was performed in two stages: primary annotation, which was a manual checking of gene descriptions provided by automated annotation, and secondary annotation, which was functional annotation by specialists in each field.

Annotation tools

Cooperative annotation work was carried out using a combination of cgaRhizoBase, a database that collects and stores genome information such as the

orientation of genes and gene descriptions, and KazusaAnnotation, which stores annotations and annotator information and provides data entry support. cgaRhizoBase has an access control function that is based on the RhizoBase / CyanoBase (http://genome.kazusa.or.jp/rhizobase/) (31) interface. This database could be accessed only by the participants in this genome annotation project, but information could be shared among the participants. Information such as gene ID, gene symbol, function, coding sequence length, protein length, gene family, family member designation, and external links is stored in the database. In addition, it is possible to find reference information on protein orthologues and homologues and to carry out sequence homology searches. All of the annotation information submitted by the participants was stored in the database through the web interface for KazusaAnnotation and integrated into the cgaRhizoBase gene information page.

Results

Annotation jamboree

An annotation jamboree was held at the Agriculture, Forestry and Fisheries Research Information Technology Conference Hall on July 20–22, 2010. Thirty-one people, including five students, from six universities and 11 research institutes participated. The results were an improvement in 2068 gene names among 6948 genes identified during the automatic annotation.

Secondary annotation in each of the specialty fields was carried out using a similar system; during this stage, approximately 600 genes were re-annotated. As part of the secondary annotation, ATP-binding cassette (ABC) transporter genes and their related proteins were classified by performing a BLASTN analysis against the ABCISSE database (http://www.pasteur.fr/recherche/unites/pmtg/abc/index.html), in which ABC proteins are phylogenetically and functionally classified based on amino acid sequences (6). Pathway analysis was also conducted through BLASTP comparisons against the KEGG PATHWAY database using the KAAS system (30).

Putative replication origin and terminus

A GC skew analysis was performed to locate the probable origin and terminus of DNA replication (15). A shift of GC skew was observed in two regions of the genome, at coordinates 200 kb and 3850 kb, as shown in the innermost circle of Fig 2.

The conserved sequence pattern required to convert a dimer chromosome to monomers after aberrant DNA duplication led to the designation of *dif*, a 28-mer sequence (4). The genome sequence of S23321 revealed that *dif* is located at coordinates 3,722,896–3,722,869 bp, near one side of the GC-skew shift. It is likely that DNA replication terminates in this region. Another shift in GC skew was detected on the opposite side of the circular chromosome from *dif*, which seems to indicate the presence of a putative origin of replication coinciding with other typical origins of replication from the *Bradyrhizobiaceae* (22). In the case of USDA110, the putative origin is positioned between genes bll0636 and blr0637. In S23321, the orthologues of these two genes were S23_1990 and S23_02010, respectively. This intergenic region showed nucleotide sequence alignment with 81% identity over 346 bp (S23321 coordinates 207,956–208,301 bp) with the corresponding region of the USDA110 sequence. This suggests that the sequence corresponding to the origin of replication is conserved in this region.

Photosynthetic genes bchE *and* bchJ

A BLAST search analysis of the genome sequence of S23321 identified no candidates for *bchE* and *bchJ*. This was unexpected because all of the genomic DNAs reported so far for photosynthetic proteobacteria contain these genes. *R. palustris* has *bchE* and *bchJ* in a tandem arrangement, although its location is far from the PGC. The gene product of *bchE* is a Mg-protoporphyrin IX monomethyl ester cyclase that functions under anaerobic conditions (52). However, this function can be compensated for by the presence of *acsF* under semi-aerobic conditions (36), and the S23321 PGC does contain *acsF*. The gene product of *bchJ* is a reductase that converts the 8-vinyl group of chlorophyll to an ethyl group (52). However, it has been reported that bacteriochlorophyll biosynthesis can be completed even when *bchJ* is knocked out, although the yield is reduced (46). These previous reports suggest that conservation of *bchE* and *bchJ* is not essential for bacteriochlorophyll biosynthesis, and that lack of

bchE and *bchJ* in S23321 does not eliminate the possibility of photosynthetic ability in this bacterium.

Respiratory chain

The genome analyses demonstrated that S23321 harbors complexes I, II, III, IV, and V of the respiratory chain (Table S5). All these genes showed the highest similarity (78–96% amino acid identity) to those of USDA110 in BLASTP analysis against the NCBI nr database. Bacterial complex I usually contains either a *nuo* or *nqr* operon containing 14 genes (53). In S23321, the genes encoding complex I (NADH dehydrogenase) consist of a *nuo* operon (S23_32620–32770) containing 14 genes. The operons of S23321 encoding complex II (succinate dehydrogenase), complex III (cytochrome bc_1 complex), and complex V (F_0F_1 ATPase), were similar to those in the USDA110 genome.

However, there are a number of differences between S23321 and USDA110 in the structure of complex IV (terminal oxidase). Six terminal oxidase complexes were found in the S23321 genome: three of these six were cytochrome *c* oxidases, whereas the other three were quinol oxidases (Table S5). The members of these six oxidase complexes shared a high degree of amino acid sequence identity with *B. japonicum* USDA110. On the other hand, no operons closely related to the *cydAB*-like genes of USDA110 (blr3728–3729) (14) were found in S23321 (5). USDA110 harbors eight terminal oxidase complexes (14), giving it one of the most highly branched respiratory chains of all aerobic prokaryotes and making it capable of functioning throughout a wide range of oxygen tensions. Of the six terminal oxidase complexes in S23321, the S23_52330–52360 operon most likely corresponds to the *fixNOQP* gene cluster in USDA110, which encodes an oxidase with an extremely high affinity for O_2 that is expressed microaerobically, *i.e.*, under extremely low oxygen levels (24, 33, 34, 37). The S23_52770–52800 operon is most closely related to the *coxWXYZ* gene cluster, which encodes a microaerobically expressed bb_3-type ubiquinol oxidase (45). The S23_06500–06530 and S23_07900–07930 operons are closely related to *cyoABCD* and *coxABCD*, respectively, which are expressed under aerobic conditions.

Low oxygen concentrations induce *fixNOQP* expression in USDA110 (14). It

has been reported that the *fixNOQP* operon and denitrification genes are regulated by the FixLJ two-component regulatory system and $FixK_2$ (2, 28, 32). S23_52400, 52410, and 52430 in S23321 are closely related to *fixL*, *fixJ*, and *fixK*$_2$ in USDA110, respectively (identity of 85.0%, 85.9%, and 89.7%, respectively). These facts suggest that S23321 is capable of functioning throughout a wide range of oxygen tensions. A detailed analysis of metabolic processes would be needed to explain why S23321 is a slow-growing bacterium in the DNB test system, since it appears from the genome analysis that S23321 has multiple energy-producing mechanisms.

It is also interesting that S23321 harbors a *fixNOQP* operon, which plays a role in symbiotic nitrogen fixation (25), because this strain is not capable of inducing nodules on siratro roots roots. Genes orthologous to *fixNOQP* of USDA110 have been identified in other Gram-negative bacteria; these have been designated *ccoNOQP* genes. CcoNOQP has been extensively studied in *Rhodobacter sphaeroides*, where it functions not only as a terminal oxidase (12), but also as a redox sensor in a signal transduction pathway controlling the expression of genes involved in photosynthesis (19, 35).

Chemotaxis

There are three clusters of chemotaxis genes in S23321: the first located at 450 kb, the second at 5876 kb, and the third at 6030 kb. The second cluster comprises a complete set of general chemotaxis genes: *cheB* (methylase), *cheR* (methyltransferase), two copies of *mcp* (methyl-accepting chemotaxis protein, or sensory receptor), two copies of *cheW* (a connecter between Mcp and CheA), *cheA* (histidine kinase), and *cheY* (sensory response regulator). However, *cheZ* (phosphatase) is missing in this organism (43), and the first and third *che* clusters of S23321 are incomplete.

In addition to the two *mcp* genes in the second cluster, there are at least 31 other *mcp* genes scattered throughout the genome. There are two *cheA* homologues between positions 4000 and 5000 kb and one *aer* (receptor for aerotaxis) gene at 5085 kb. It is not clear at the moment whether these genes are expressed.

Protein secretion systems

Among the types of protein secretion systems known in other species, genes corresponding to a type II secretion system (T2SS) and a type III secretion system

(T3SS) were found in S23321, but no genes were found for a type I secretion system (T1SS) or a type IV secretion system (T4SS).

There are two types of secretion systems that use the signal peptidase: the general secretion pathway (GSP) and the type IV pilus assembly system. Within the GSP, the *secA, secB, secD, secE, secF, secG*, and *secY* genes were found scattered throughout the genome (Fig. S5). On the other hand, the genes required for type IV pilus assembly, *ctpA* to *ctpI*, are clustered at 1,062 kb. Several *pilA* genes, which encode the major component of the pilus, are found at several sites in the genome (Fig. S5). The three *pilA* genes are likely intact, because of the presence of typical SD (Shine-Dalgano) sequences (Fig. S10). However, biochemical tests are necessary to determine whether these genes are used for construction of the pilus. It should be noted that two other *Bradyrhizobium* strains, ORS278 and BTAi1, possess only one *pilA* gene.

There is only one type of T3SS in S23321, the flagellar assembly system. Other T3SS, *e.g.*, for pathogenicity or nodule formation, are missing from this species, though the complete set of T3SS genes is found in *B. japonicum*.

Genes for the twin-arginine translocation (TAT) system are clustered within the *secDFG* gene cluster at 3,507 kb: these genes include *tatA*, *tatB*, and *tatC* (Fig. S5).

ABC transporters and macromolecule biodegradation

ABC systems constitute one of the most abundant superfamilies of proteins. They are involved not only in the import and export of a wide variety of substances but also in many other cellular processes. The S23321 genome contains 492 ORFs for ABC proteins and their interacting partners. Of these 492 ORFs, 455 and 27 ORFs are predicted to be involved in uptake and efflux, respectively. As in other prokaryotes, most of the putative importer genes form clusters, each of which encodes the components for a particular import system (Table S6).

The composition of the ABC systems in S23321 is similar to that in *B. japonicum* USDA110, which possesses orthologues of 382 of the 492 ORFs for ABC systems found in the S23321 genome (Table S6). The ABC systems of these two *Bradyrhizobium* species, in contrast to those of the typical saprophytic soil bacterium *Streptomyces coelicolor* A3(1) (3), have few uptake systems for oligosaccharides and polyols (Table S7), which would be produced via degradation of polysaccharides by

secreted glycosyl hydrolases. As in other members of the *Rhizobiaceae*, S23321 seems to have relatively few genes for glycosyl hydrolases (GH) compared with *S. coelicolor* A3(1). S23321 has only one putative alpha-amylase gene (S23_15430), which is surrounded by genes for a putative glycosyl hydrolase (S23_15420) belonging to GH family 13, and for putative glycogen-debranching and -branching enzymes (S23_15440 and 15450, respectively).

The ABC system of S23321, like that of USDA110, is relatively rich in genes encoding HAA (hydrophobic amino acids and amides) and OPN (oligopeptides and nickel) family transporters (which might be involved in uptake of amino acids and oligopeptides, respectively), when compared to those of *Pseudomonas putida* KT24440 and *S. coelicolor* A3(1) (Table S7). S23321 contains putative genes for 35 proteases, 15 of which are predicted to be secreted. S23321 might thus prefer proteins, oligopeptides, and amino acids to saccharides as a source of energy. Alternatively, S23321 might preferentially inhabit the rhizoplane (root surface) and/or rhizosphere, where amino acids, monosaccharides, and other low-molecular-weight substances would be supplied from plant roots, and may directly utilize those compounds by uptake via ABC transporters.

Carbon and nitrogen metabolism

S23321 possesses all of the genes required for glycolysis and gluconeogenesis, the citrate cycle (TCA cycle, Krebs cycle), the pentose phosphate pathway, the glyoxylate cycle, fatty acid biosynthesis, and fatty acid metabolism (Table S8), suggesting that this bacterium is able to use many carbon substrates through multiple catabolic pathways to adapt to a free-living life in the soil environment.

For monosaccharide transport, there is one predicted ABC transporter in the S23321 genome: a ribose transporter encoded by S23_46840 to S23_46870. However, since a multiple-sugar ABC transporter encoded by the *chvE* operon in *Brucella suis* had the ability to transport many types of monosaccharides (1), it is possible that S23321 also takes in monosaccharides through the same strategy.

Compared with *B. japonicum* USDA110, S23321 lacks genes for several enzymes involved in nitrogen metabolism, such as L-asparaginase, asparagine synthase, glutamate dehydrogenase, and nitrilase (Table S9). Thus, S23321 has fewer genes

involved in nitrogen metabolism than its close relatives USDA110, BTAi1, and ORS278. The absence of these genes in S23321 includes the lack of a *nosZ* gene for the final step of denitrification, and it is thus unable to consume nitrile or synthesize L-asparagine. It is likely that L-Asn-tRNA^{Asn} is produced from L-Asp-tRNA^{Asn} by aspartyl-tRNA amidotransferase encoded by *gatA*, *gatB*, and *gatC*, but not directly synthesized from L-asparagine (Table S10), as has been shown for *Deinococcus radiodurans* (29). It is likely that *B. japonicum* USDA110 and *Bradyrhizobium* sp. BTAi1 and ORS278 have also evolved the same mechanism to produce L-Asn-tRNA^{Asn} (Table S10). S23321 probably senses the level of environmental nitrogen via two NtrC-family two-component systems (S23_36930–36940 and S23_36910–36920), and takes in ammonia through two transporters encoded by S23_02240 and S23_02290. In addition, three genes were predicted to encode PII proteins (S23_02250, S23_02300 and S23_32340), which regulate nitrogen assimilation (8, 11).

Degradation of aromatics

In soil, microorganisms usually compete for nutrients, including aromatic compounds, to help them survive. There are many reports of utilization of aromatics by *Bradyrhizobium* spp. for growth (21, 42, 49). *B. japonicum* USDA110 is able to degrade and utilize 4-hydroxybenzoate and vanillate, both of which are intermediates in the degradation of humic substances and lignin (16, 44). In the S23321 genome, there are several sets of gene homologues for degrading 4-hydroxybenzoate and vanillate (Table S11). Thus, S23321 is likely able to degrade vanillate and 4-hydroxybenzoate (Fig. S6).

When vanillate is oxidized to protocatechuate, formaldehyde is produced as a byproduct (Fig. S6). USDA110 metabolizes formaldehyde via a glutathione-dependent pathway (16). The S23321 genome contains genes whose predicted amino acid sequences are at least 84–96% identical to those of the genes for metabolizing formaldehyde in USDA110 (Table S11). This result indicates that S23321 has the same metabolic pathway for formaldehyde as USDA110.

The chromosomal organization of genes for degrading vanillate, 4-hydroxybenzoate, and formaldehyde is well conserved between S23321 and USDA110 (Fig. S7), supporting the hypothesis that S23321 has the same metabolic pathways for these substrates as USDA110.

From genome data analysis performed with the KEGG PATHWAY database (30), gene homologues for the degradation of toluene and 4-chlorocatechol were found in S23321 (Fig. S8). In fact, some toluene-degrading *Bradyrhizobium* spp. have been isolated from groundwater polluted with toluene (7), and utilization of 4-chlorocatechol by *B. japonicum* has been reported (42).

Chemoautotrophy

Genes for chemoautotrophy were surveyed in the S23321 genome because *B. japonicum* USDA110 is able to grow chemoautotrophically. Thiosulfate-oxidizing *sox* genes were found at four loci (I, II, III, and IV) in the USDA110 genome. The cells of USDA110 were able to grow chemoautotrophically using thiosulfate as an electron donor. Thiosulfate oxidation in USDA110 likely depends on *sox* locus I. When we searched the genome of S23321, we found homologues to *sox* loci II and III, but not to locus I or IV (Fig. S9A). The gene organization at *sox* loci II and III in S23321 was very similar to that of photosynthetic stem-nodulating *Bradyrhizobium* sp. BTAi1 and ORS278 and to *B. japonicum* USDA110 (Fig. S9A).

In USDA110, a $soxY_2$ deletion mutation gene at *sox* locus II, which is homologous to the Sox system in green sulfur bacteria, showed phenotypes similar to wild-type USDA110 (26). In the amino acid sequences of SoxY in USDA110, the GGCGG motif, essential for the binding of thiosulfate to SoxY, was not conserved in the C terminus of the predicted protein product of $soxY_3$ (part of locus III). Although the product of $soxY_3$ of S23321 is predicted to contain the GGCGG motif at the C terminus (Fig. S9A), the complete *soxWXYZABCD* gene cluster, essential for thiosulfate oxidation in the Sox system, was not found at *sox* locus III. In addition, no genes for uptake hydrogenase genes were found in the S23321 genome. These results suggest that S23321 is unable to grow chemoautotrophically using either thiosulfate or H_2 as an electron donor.

Carbon monoxide dehydrogenase (*cox*) gene homologues were found clustered in the S23321 genome (Fig. S9C). USDA110, which also contains *cox* genes, has carbon monoxide dehydrogenase activity (23). Although it has not yet been determined whether CO oxidation in USDA110 depends on *cox* genes, we predict that S23321 could grow chemoautotrophically using CO as an electron donor. In USDA110, a

mutant of *cbbL*, which encodes the large subunit of ribulose 1,5-bisphosphate carboxylase/oxygenase (RuBisCO), was unable to grow chemoautotrophically (27). A *cbb* cluster was found in the S23321 genome, with gene organization identical to that of USDA110 (Fig. S9B). Therefore, it is likely that S23321 has the capability to fix CO_2 via RuBisCO using CO as an electron donor.

Non-coding RNAs

Non-coding RNAs that have homology with those of *B. japonicum* USDA110 were found in the S23321 genome (Table S12). These include predicted genes for a ribozyme, a class A RNase P, and putative RNA sensors such as glycine riboswitches (three copies), cobalamin riboswitches (four copies), *S*-adenosylmethionine (SAM) riboswitches (two copies), a thiamine pyrophosphate (TPP) riboswitch (one copy), a flavin mononucleotide (FMN) riboswitch (FMN element) (one copy), and a repression of heat shock gene expression (ROSE) element (one copy). The S23321 genome also contains a gene for 6S RNA, which binds to bacterial RNA polymerase holoenzyme and modulates promoter use (51), and one for the RNA component of a bacterial signal recognition particle (SRP), which plays an important role in membrane targeting. The S23321 SRP RNA is 97 bp in length and has the same sequence as that of USDA110, which has a predicted secondary structure similar to that of *E. coli* (38). Several predicted regulatory regions such as *suhB* RNA elements (three copies), a *speF* element (one copy), and a *serC* element (one copy), which have been identified in the intergenic regions of many *Alphaproteobacteria*, were also found in the S23321 genome (9). These results indicate that S23321 may use the same functions of non-coding RNAs as *B. japonicum* to modulate gene expression and tRNA processing.

References

1. Alvarez-Martinez, M. T., J. Machold, C. Weise, H. Schmidt-Eisenlohr, C. Baron, and B. Rouot. 2001. The *Brucella suis* homologue of the *Agrobacterium tumefaciens* chromosomal virulence operon *chvE* is essential for sugar utilization but not for survival in macrophages. J. Bacteriol. 183:5343-5351.
2. Bedmar, E. J., E. F. Robles, and M. J. Delgade. 2005. The complete

denitrification pathway of the symbiotic, nitrogen-fixing bacterium *Bradyrhizobium japonicum*. Biochem. Soc. Trans. 33:141-144.

3. Bentley, S. D., K. F. Chater, A. M. Cerdeno-Tarraga, et al. 2002. Complete genome sequence of the model actinomycete *Streptomyces coelicolor* A3(2). Nature 417:141-7.
4. Blakely, G. W., Davidson, A. O., and Sherratt, D. J. 2000. Sequential strand exchange by XerC and XerD during site-specific recombination at *dif,* J. Biol.Chem. 275:9930-9936
5. Bott, M., M. Bolliger, and H. Hennecke. 1990. Genetic analysis of the cytochrome *c-aa*$_3$ branch of the *Bradyrhizobium japonicum* respiratory chain. Mol. Microbiol. 4:2147-2157.
6. Bouige, P., D. Laurent, L. Piloyan, and E. Dassa. 2002. Phylogenetic and functional classification of ATP-binding cassette (ABC) systems. Curr. Protein. Pept. Sci. 3:541-59.
7. Cavalca, L., E. Dell'Amico, and V. Andreoni. 2004. Intrinsic bioremediability of an aromatic hydrocarbon-polluted groundwater: diversity of bacterial population and toluene monooxygenase genes. Appl. Microbiol. Biotechnol. 64:576-87.
8. Commichau, F. M., K. Forchhammer, and J. Stulke. 2006. Regulatory links between carbon and nitrogen metabolism. Curr. Opin. Microbiol. 9:167-72.
9. Corbino, K. A., J. E. Barrick, J. Lim, R. Welz, B. J. Tucker, I. Puskarz, M. Mandal, N. D. Rudnick, and R. R. Breaker. 2005. Evidence for a second class of S-adenosylmethionine riboswitches and other regulatory RNA motifs in alpha-proteobacteria. Genome Biol. 6(8):R70.
10. Elsik, C. G., K. C. Worley, L. Zhang, N. V. Milshina, H. Jiang, J. T. Reese, K. L. Childs, A. Venkatraman, C. M. Dickens, G. M. Weinstock, and R. A. Gibbs. 2006. Community annotation: procedures, protocols, and supporting tools. Genome Res. 16:1329-33.
11. Forchhammer, K. 2004. Global carbon/nitrogen control by PII signal transduction in cyanobacteria: from signals to targets. FEMS Microbiol. Rev. 28:319-33.
12. Garcia-Horsman, J. A., E. Berry, J. P. Shapleigh, J. O. Alben, and R. B. Gennis. 1994. A novel cytochrome c oxidase from *Rhodobacter sphaeroides* that lacks CuA. Biochemistry 33:3113-9.
13. Giraud, E., L. Moulin, D. Vallenet et al. 2007. Legumes symbioses: absence of Nod genes in photosynthetic bradyrhizobia. Science 316:1307-12.
14. Gottfert, M., H. Hennecke, and S. Tabata. 2005. Facets of the *Bradyrhizobium japonicum* 110 genome. (ed.) R. Palacios, and W.E. Newton. In Genomes and Genomics of Nitrogen-fixing Organisms. 99-111.
15. Grant, J. R., and P. Stothard. 2008. The CGView Server: a comparative genomics tool for circular genomes. Nucleic Acids Res. 36:W181-4.
16. Ito, N., M. Itakura, S. Eda et al. 2006. Global gene expression in *Bradyrhizobium japonicum* cultured with vanillin, vanillate, 4-hydroxybenzoate and protocatechuate. Microbes Environ. 21:240-250.

17. Kamagata Y, Fulthorpe RR, Tamura K, Takami H, Forney LJ, TieJe JM. (1997). Pristine environments harbor a new group of oligotrophic 2,4-dichlorophenoxyacetic acid-degrading bacteria. Appl Environ Microbiol 63: 2266–2272.
18. Kaneko, T., Y. Nakamura, S. Sato et al. 2000. Complete genome structure of the nitrogen-fixing symbiotic bacterium *Mesorhizobium loti*. DNA Res. 7:331-338.
19. Kim, Y. J., I. J. Ko, J. M. Lee, H. Y. Kang, Y. M. Kim, S. Kaplan, and J. I. Oh. 2007. Dominant role of the *cbb3* oxidase in regulation of photosynthesis gene expression through the PrrBA system in *Rhodobacter sphaeroides* 2.4.1. J. Bacteriol. 189:5617-25.
20. Larimer, F. W., P. Chain, L. Hauser et al. 2004. Complete genome sequence of the metabolically versatile photosynthetic bacterium *Rhodopseudomonas palustris*. Nat. Biotechnol 22:55-61.
21. Latha, S., and A. Mahadevan. 1997. Role of rhizobia in the degradation of aromatic substances. World J. Microbiol. Biotechnol. 13:601-607.
22. Lee, K.B. de Backer, P. Aono, T. Liu, C.T. Suzuki, S. Suzuki, T. Kaneko, T. Yamada, M. Tabata, S. Kupfer, D.M. *et al*. 2008. The genome of the versatile nitrogen fixer *Azorhizobium caulinodans* ORS571. BMC Genomics 9:271.
23. Lorite, M. J., J. Tachil, J. Sanjuan, O. Meyer, and E. J. Bedmar. 2000. Carbon monoxide dehydrogenase activity in *Bradyrhizobium japonicum.* Appl. Environ. Microbiol. 66:1871-6.
24. Maier, R. J., and D. M. Merberg. 1982. *Rhizobium japonicum* mutants that are hypersensitive to repression of H_2 uptake by oxygen. J. Bacteriol. 150:161-7.
25. Mandon, K., P. A. Kaminski, and C. Elmerich. 1994. Functional analysis of the *fixNOQP* region of *Azorhizobium caulinodans*. J. Bacteriol. 176:2560-2568.
26. Masuda, S., S. Eda, S. Ikeda, H. Mitsui, and K. Minamisawa. 2010. Thiosulfate-dependent chemolithoautotrophic growth of *Bradyrhizobium japonicum.* Appl. Environ. Microbiol. 76:2402-9.
27. Masuda, S., S. Eda, C. Sugawara, H. Mitsui, and K. Minamisawa. 2010. The *cbbL* gene is required for thiosulfate-dependent autotrophic growth of *Bradyrhizobium japonicum*. Microbes. Environ. 25:220-3.
28. Mesa, S., F. Hauser, M. Friberg, E. Malaguti, H. M. Fischer, and H. Hennecke. 2008. Comprehensive assessment of the regulons controlled by the FixLJ-FixK2-FixK1 cascade in *Bradyrhizobium japonicum.* J. Bacteriol. 190:6568-79.
29. Min, B., J. T. Pelaschier, D. E. Graham, D. Tumbula-Hansen, and D. Soll. 2002. Transfer RNA-dependent amino acid biosynthesis: an essential route to asparagine formation. Proc. Natl. Acad. Sci. U S A 99:2678-83.
30. Moriya, Y., M. Itoh, S. Okuda, A. C. Yoshizawa, and M. Kanehisa. 2007. KAAS: an automatic genome annotation and pathway reconstruction server. Nucleic Acids Res. 35:W182-5.
31. Nakao, M., S. Okamoto, M. Kohara, T. Fujishiro, T. Fujisawa, S. Sato, S. Tabata, T. Kaneko, and Y. Nakamura. CyanoBase: the cyanobacteria genome database

update 2010. Nucleic Acids Res. 38:D379-81.
32. Nellen-Anthamatten, D., P. Rossi, O. Preisig, I. Kullik, M. Babst, H. M. Fischer, and H. Hennecke. 1998. *Bradyrhizobium japonicum* FixK2, a crucial distributor in the FixLJ-dependent regulatory cascade for control of genes inducible by low oxygen levels. J. Bacteriol. 180:5251-5.
33. O'Brian, M. R., and R. J. Maier. 1982. Electron transport components involved in hydrogen oxidation in free-living *Rhizobium japonicum*. J. Bacteriol. 152:422-30.
34. O'Brian, M. R., and R. J. Maier. 1983. Involvement of cytochromes and a flavoprotein in hydrogen oxidation in *Rhizobium japonicum* bacteroids. J. Bacteriol. 155:481-7.
35. Oh, J. I., and S. Kaplan. 2002. Oxygen adaptation. The role of the CcoQ subunit of the cbb_3 cytochrome *c* oxidase of *Rhodobacter sphaeroides* 2.4.1. J. Biol. Chem. 277:16220-8.
36. Pinta, V., M. Picaud, F. Reiss-Husson, and C. Astier. 2002. R*ubrivivax gelatinosus acsF* (previously orf358) codes for a conserved, putative binuclear-iron-cluster-containing protein involved in aerobic oxidative cyclization of Mg-protoporphyrin IX monomethylester. J. Bacteriol. 184:746-53.
37. Preisig, O., D. Anthamatten, and H. Hennecke. 1993. Genes for a microaerobically induced oxidase complex in *Bradyrhizobium japonicum* are essential for a nitrogen-fixing endosymbiosis. Proc. Natl. Acad. Sci. U S A 90:3309-13.
38. Regalia, M., M. A. Rosenblad, and T. Samuelsson. 2002. Prediction of signal recognition particle RNA genes. Nucleic Acids Res. 30:3368-77.
39. Saito, A., H. Mitsui, R. Hattori, K. Minamisawa, and T. Hattori. 1998. Slow-growing and oligotrophic soil bacteria phylogenetically close to *Bradyrhizobium japonicum*. FEMS Microbiol. Ecol. 25:277-286.
40. Sameshima R, Isawa T, Sadowsky MJ, Hamada T, Kasai H, Shutsrirung A et al. 2003. Phylogeny and distribution of extra-slow-growing *Bradyrhizobium japonicum* harboring high copy numbers of RSa, RSb and IS1631. FEMS Microbiol Ecol 44: 191–202.
41. Sameshima-Saito R, Chiba K, Minamisawa K. 2006. Correlation of denitrifying capability with the existence of *nap*, *nir*, *nor* and *nos* genes in diverse strains of soybean bradyrhizobia. Microbes Environ 21:174–184.
42. Sonia, V., M. Häggblom, and L. Young. 2002. Biodegradation of aromatic and aliphatic compounds by rhizobial species. Soil Science 167:802-810.
43. Sourjik, V., and J. P. Armitage. Spatial organization in bacterial chemotaxis. EMBO J .29:2724-33.
44. Sudtachat, N., N. Ito, M. Itakura, S. Masuda, S. Eda, H. Mitsui, Y. Kawaharada, and K. Minamisawa. 2009. Aerobic vanillate degradation and C1 compound metabolism in *Bradyrhizobium japonicum*. Appl. Environ. Microbiol. 75:5012-7.
45. Surpin, M. A., M. Lubben, and R. J. Maier. 1996. The *Bradyrhizobium japonicum coxWXYZ* gene cluster encodes a bb_3-type ubiquinol oxidase. Gene

183:201-6.
46. Suzuki, J. Y., and C. E. Bauer. 1995. Altered monovinyl and divinyl protochlorophyllide pools in *bchJ* mutants of *Rhodobacter capsulatus*. Possible monovinyl substrate discrimination of light-independent protochlorophyllide reductase. J. Biol. Chem. 270:3732-40.
47. Tamura, K., D. Peterson, N. Peterson, G. Stecher, M. Nei, and S. Kumar. MEGA5: Molecular evolutionary genetics analysis using maximum likelihood, evolutionary distance, and maximum parsimony methods. Mol. Biol. Evol.
48. Thompson, J. D., D. G. Higgins, and T. J. Gibson. 1994. CLUSTAL W: improving the sensitivity of progressive multiple sequence alignment through sequence weighting, position-specific gap penalties and weight matrix choice. Nucleic Acids Res. 22:4673-80.
49. Van Rossum, D., F. P. Schuurmans, M. Gillis, A. Muyotcha, H. W. Van Verseveld, A. H. Stouthamer, and F. C. Boogerd. 1995. Genetic and phenetic analyses of *Bradyrhizobium* strains nodulating peanut (*Arachis hypogaea* L.) roots. Appl. Environ. Microbiol. 61:1599-609.
50. Van Berkum P, Fuhrmann JJ. 2000. Evolutionary relationships among the soybean bradyrhizobia reconstructed from 16S rRNA gene and internally transcribed spacer region sequence divergence. Int J Syst Evol Microbiol 50: 2165–2172.
51. Wassarman, K. M., and G. Storz. 2000. 6S RNA regulates *E. coli* RNA polymerase activity. Cell 101:613-23.
52. Willows, R. D., and A. M. Kriegel. 2009. Biosynthesis of bacteriochlorophylls in purple bacteria, p. 57-79. In C. N. Hunter, F. Daldal, M. C. Thurnauer and J. T. Beatty (ed.), The purple phototrophic bacteria. Springer, Dordrecht, The Netherlands.
53. Yagi, T., T. Yano, S. Di Bernardo, and A. Matsuno-Yagi. 1998. Procaryotic complex I (NDH-1), an overview. Biochim. Biophys. Acta. 1364:125-33.

Legends of supplementary figures and tables:

Fig. S1. Inoculation of *Bradyrhizobium* sp. S23321 and *B. japonicum* USDA110 onto *Macroptilium atropurpureum* 'Siratro'. Plants inoculated with *Bradyrhizobium* sp. S23321 or *B. japonicum* USDA110 were grown at 25 ^{o}C using nitrogen-free nutrient-solution (see text). Plant phenotypes were photographed. Nodule numbers and nodule weight were determined in triplicate (values are means ± standard deviation (SD)). *Bradyrhizobium* sp. S23321 formed no nodules on a host legume (*M. atropurpureum* 'Siratro') for a broad range of symbiotic bradyrhizobia.

Fig. S2. Comparison of the genome sequences of strains S23321 and USDA110. The genome of *Bradyrhizobium* sp. S23321 was compared with that of *B. japonicum* USDA110 by the MUMmer (a) and GenomeMatcher programs (b–d). (a) Syntenic dot plot of *Bradyrhizobium* sp. strain S23321 (*x*-axis) and strain USDA110 (*y*-axis). The symbiosis islands in USDA110 are shown. Regions marked (b)–(d) are shown in detail below. (b) Alignment of the genome sequences of strains S23321 (6.47–6.64 Mb) and USDA110 (1.52–1.69 Mb). (c) Alignment of the genome sequences of strains S23321 (6.00– 6.08 Mb) and USDA110 (2.32–2.40 Mb). (d) Alignment of the genome sequences of strains S23321 (4.70–6.50 Mb) and USDA110 (2.25–8.50 Mb).

Fig. S3. Comparison of the S23321, USDA110, and USDA6 genome regions corresponding to the symbiosis island "Locus C" in the *B. japonicum* genome. The Locus C region of *B. japonicum* was highly conserved between USDA110 (8,974,971–0–70,365 bp) and USDA6 (9,113,996–0–70,356 bp). However, *Bradyrhizobium* sp. S23321 lacks the Locus C region even though the boundary regions of Locus C are conserved at coordinate 775 kb. These results suggest that the ancestor of USDA110 and USDA6 acquired the Locus C region during its evolution.

Fig. S4. Phylogenetic tree based on amino acid sequences of the bacteriochlorophyll biosynthesis enzyme BchH. The programs ClustalX (48) and MEGA (47) were used for the analysis. The tree was constructed by the neighbor-joining method, applying the p-distance parameter as a distance estimator. Gaps were omitted from the calculations. Bootstrap values are shown in the figure.

Fig. S5. Physical maps of genes for flagella (A), chemotaxis (B), and secretion (C) in the S23321 genome. The *pilA* gene (C) encodes a protein for type IV pilus. Asterisks denote ORFs encoding proteins with unknown function.

Fig. S6. Pathways and gene homologues from S23322 involved in the degradation of vanillate and 4-hydroxybenzoate.

Fig. S7. Organization of genes involved in degrading vanillate, 4-hydroxybenzoate, and formaldehyde in the genomes of *Bradyrhizobium* sp. S23321 and *B. japonicum* USDA110. Gray and black arrows show degradation genes and regulatory genes, respectively.

Fig. S8. Pathways and gene homologues from S23321 involved in the degradation of toluene and 4-chlorocatechol.

Fig. S9. Physical maps of the genes related to chemoautotrophic growth in the genome of *Bradyrhizobium* sp. S23321 and other members of the *Bradyrhizobiaceae* (*B. japonicum* USDA110, *Bradyrhizobium* sp. BTAi1, *Bradyrhizobium* sp. ORS278, and *Rhodopseudomonas palustris* CGA009). (A) *sox* genes, (B) *cbb* genes, (C) *cox* genes.

Fig. S10. Putative SD (Shine-Dalgarno) sequences (underlined) of the three *pilA* genes in the S23321genome. Start codons of the *pilA* genes are shown in red.

Table S1. Bacterial strains used for phylogenetic analysis.

Table S2. Comparison of flagella genes in several bradyrhizobial genomes

Table S3. List of genes in S23321 found on the symbiosis islands of the USDA110 genome

Table S4. Comparison of genes related to stress response in S23321 and its relatives.

Table S5. Genes for respiratory chains in the S23321 genome.

Table S6. Genes for ABC (ATP-binding cassette) proteins and their interacting partners in the *Bradyrhizobium* sp. S23321 genome.

Table S7. Compositions of ABC systems in the genomes of *Bradyrhizobium* sp. S23321 and related species.

Table S8. Genes for major carbon metabolism pathways in the genome of *Bradyrhizobium* sp. S23321.

Table S9. Comparison of the genes for nitrogen metabolism between *Bradyrhizobium* sp. S23321 and its close relatives.

Table S10. Comparison of the genes for L-Asn-tRNA biosynthesis between S23321 and its close relatives.

Table S11. Comparison of genes for degradation of vanillin and related compounds between *Bradyrhizobium* sp. S23321 and *B. japonicum* USDA110.

Table S12. Non-coding RNA in *Bradyrhizobium* sp. S23321

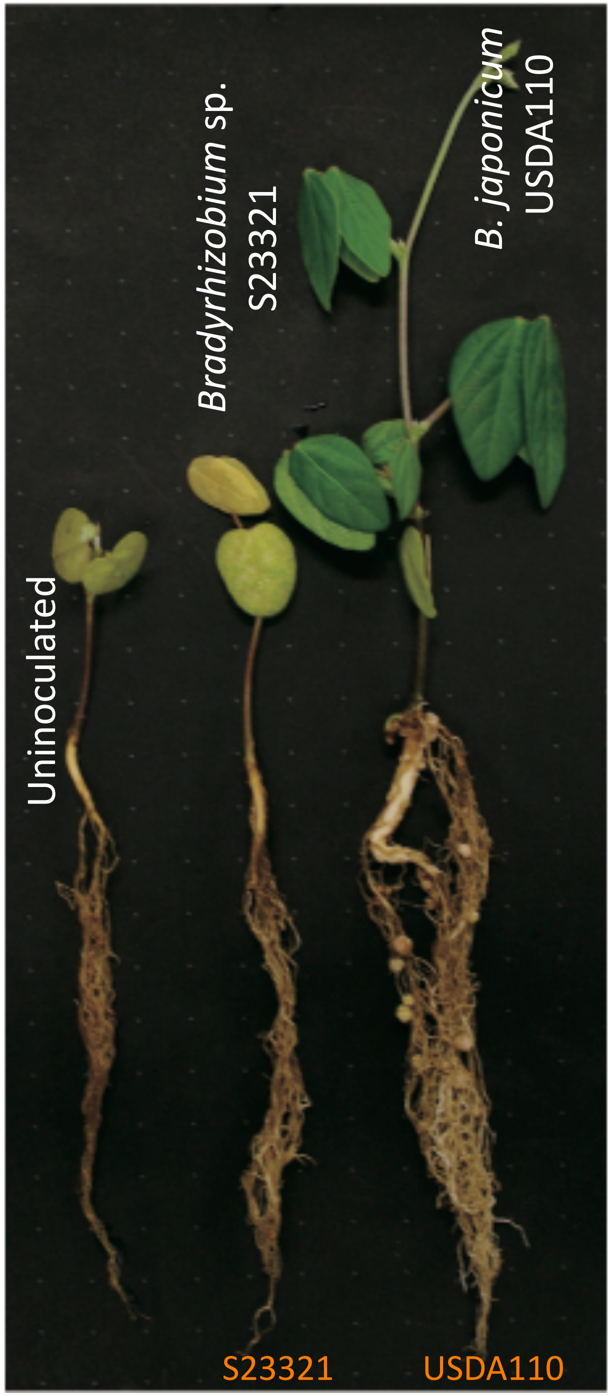

Nodulated root system

Uninoculated	*Bradyrhizobium* sp. S23321	*B. japonicum* USDA110

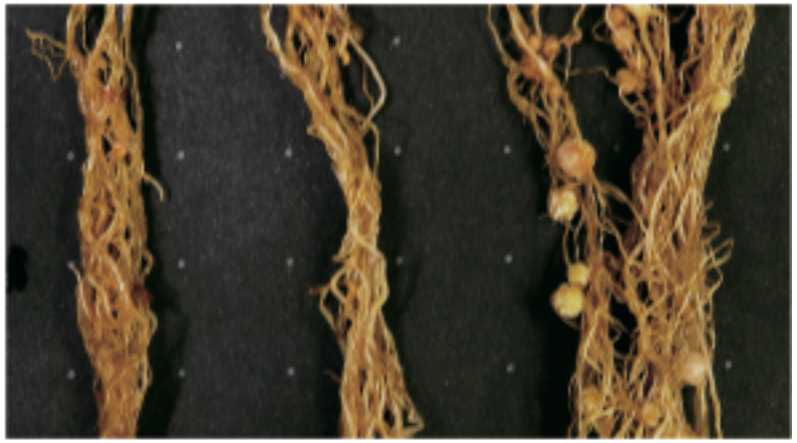

	Bradyrhizobium sp. S23321	*B. japonicum* USDA110
Number of nodules (plant $^{-1}$)	0	44 ± 10
Nodule fresh weight (plant $^{-1}$)	0	219 ± 151

Fig. S1. Inoculation of *Bradyrhizobium* sp. S23321 and *B. japinicum* USDA110 to *Macroptililium atropurpureum* cv. Siratro. Plants inoculated with *Bradyrhizobium* sp. S23321 or *B. japinicum* USDA110 were grown at 25°C using nitrogen-free nutrient-solution (see text). Plant phenotypes were photographed. Nodule numbers and nodule weight were determined with triplicates (means ± SD). *Bradyrhizobium* sp. S23321 formed no nodule to a broad-range legume (*Macroptililium atropurpureum* cv. Siratro) for symbiotic bradyrhizobia.

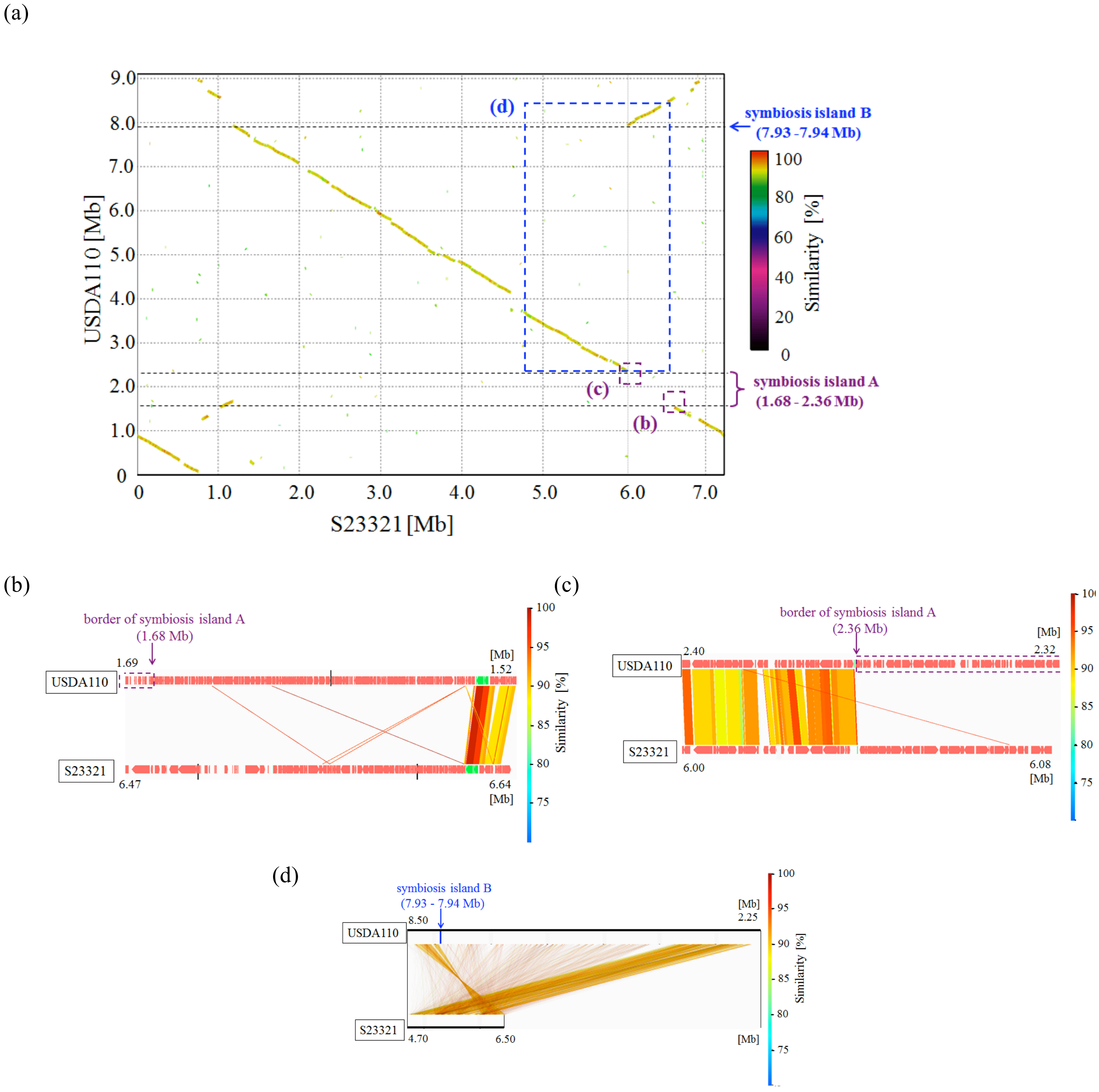

Fig. S2. Comparison of the genome sequences of strains S23321 and USDA110. The genome of *Bradyrhizobium* sp. S23321 was compared with that of *B. japonicum* USDA110 by the MUMmer (a) and GenomeMatcher programs (b–d). (a) Syntenic dot plot of *Bradyrhizobium* sp. strain S23321 (*x*-axis) and strain USDA110 (*y*-axis). The symbiosis islands in USDA110 are shown. Regions marked (b)–(d) are shown in detail below. (b) Alignment of the genome sequences of strains S23321 (6.47–6.64 Mb) and USDA110 (1.52–1.69 Mb). (c) Alignment of the genome sequences of strains S23321 (6.00– 6.08 Mb) and USDA110 (2.32–2.40 Mb). (d) Alignment of the genome sequences of strains S23321 (4.70–6.50 Mb) and USDA110 (2.25–8.50 Mb).

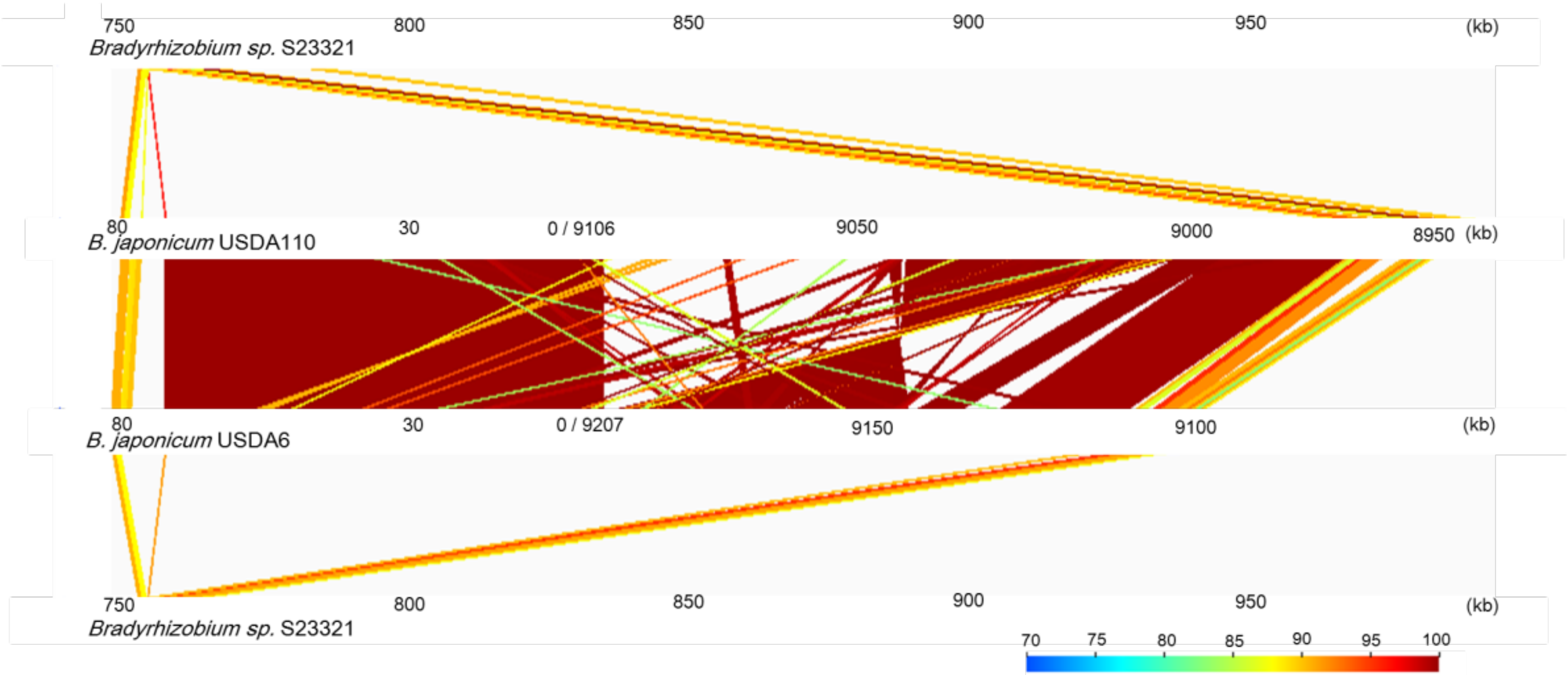

Fig. S3. Comparison of the S23321, USDA110, and USDA6 genome regions corresponding to the symbiosis island “Locus C” in the *B. japonicum* genome. The Locus C region of *B. japonicum* was highly conserved between USDA110 (8,974,971–0–70,365 bp) and USDA6 (9,113,996–0–70,356 bp). However, *Bradyrhizobium* sp. S23321 lacks the Locus C region even though the boundary regions of Locus C are conserved at coordinate 775 kb. These results suggest that the ancestor of USDA110 and USDA6 acquired the Locus C region during its evolution.

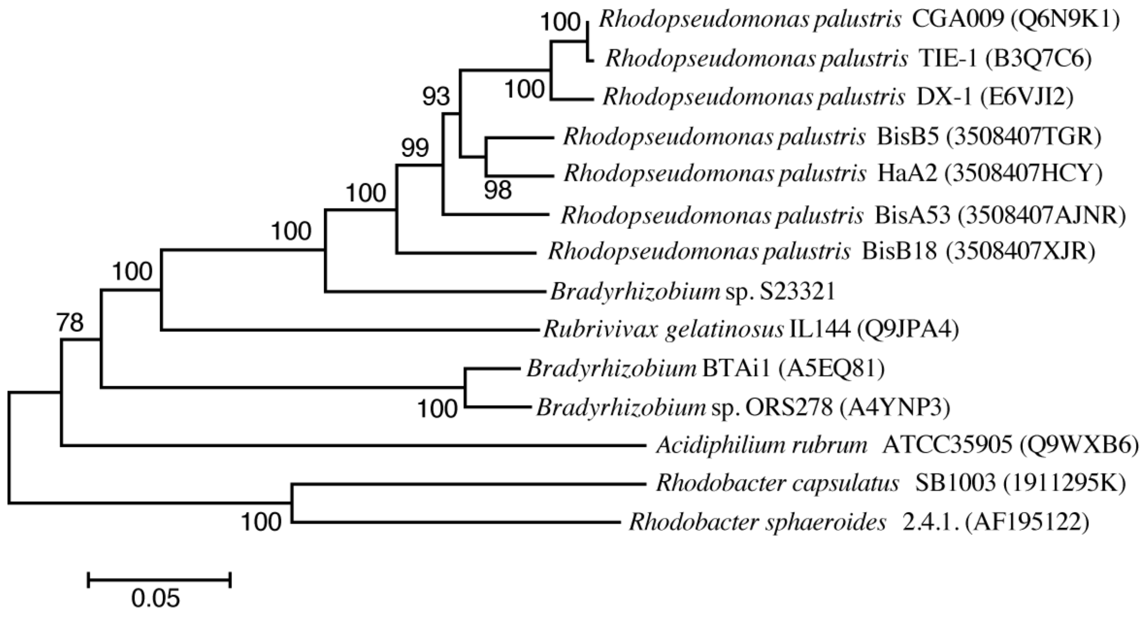

Fig. S4. Phylogenetic tree based on amino acid sequences of the bacteriochlorophyll biosynthesis enzyme BchH. The programs ClustalX (48) and MEGA (47) were used for the analysis. The tree was constructed by the neighbor-joining method, applying the p-distance parameter as a distance estimator. Gaps were omitted from the calculations. Bootstrap values are shown in the figure.

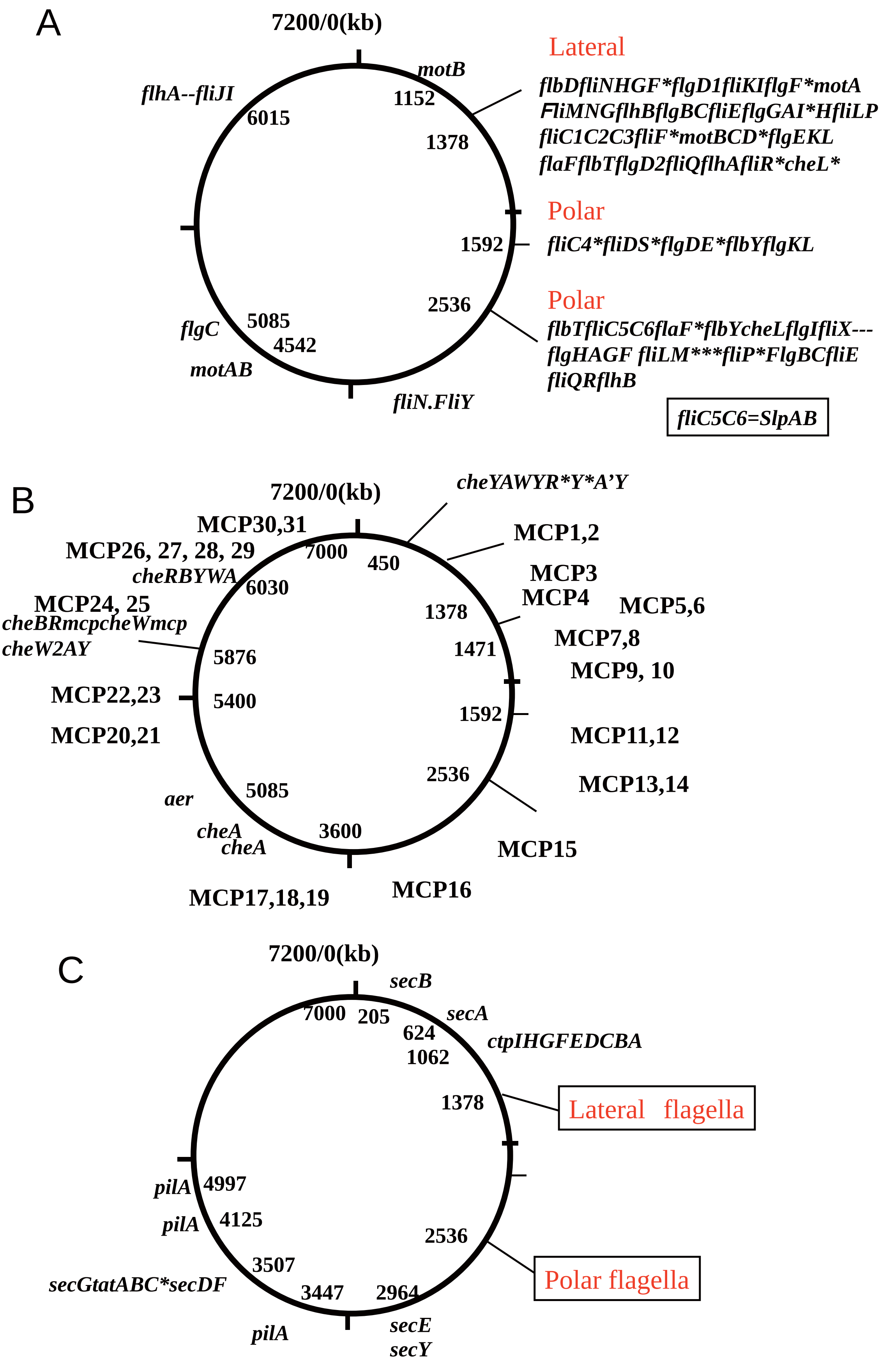

Fig. S5. Physical maps of genes for flagella (A), chemotaxis (B), and secretion (C) in the S23321 genome. The ***pilA*** gene (C) encodes a protein for type IV pilus. Asterisks denote ORFs encoding proteins with unknown function.

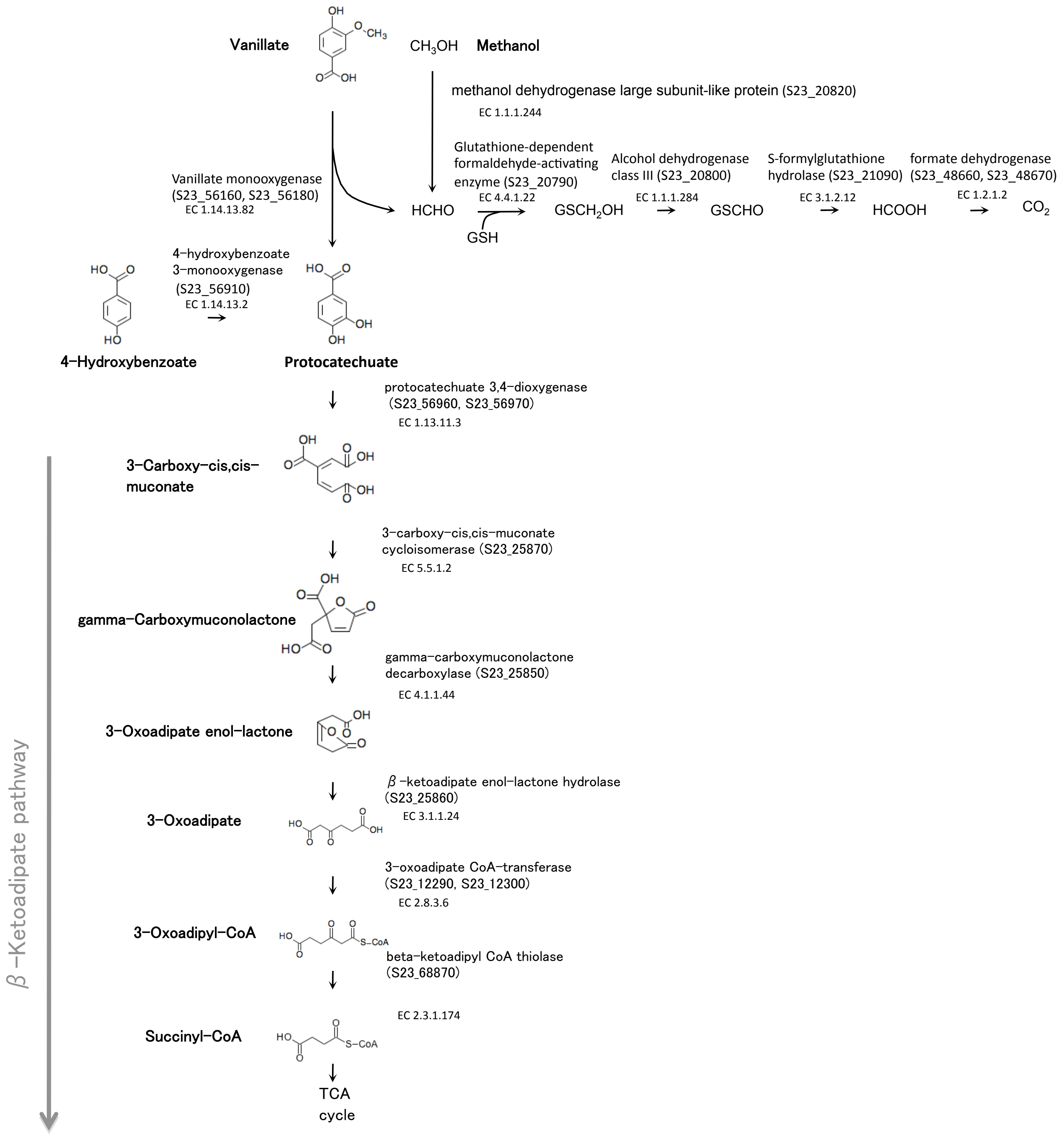

Fig. S6. Pathways and gene homologues from S23322 involved in the degradation of vanillate and 4-hydroxybenzoate.

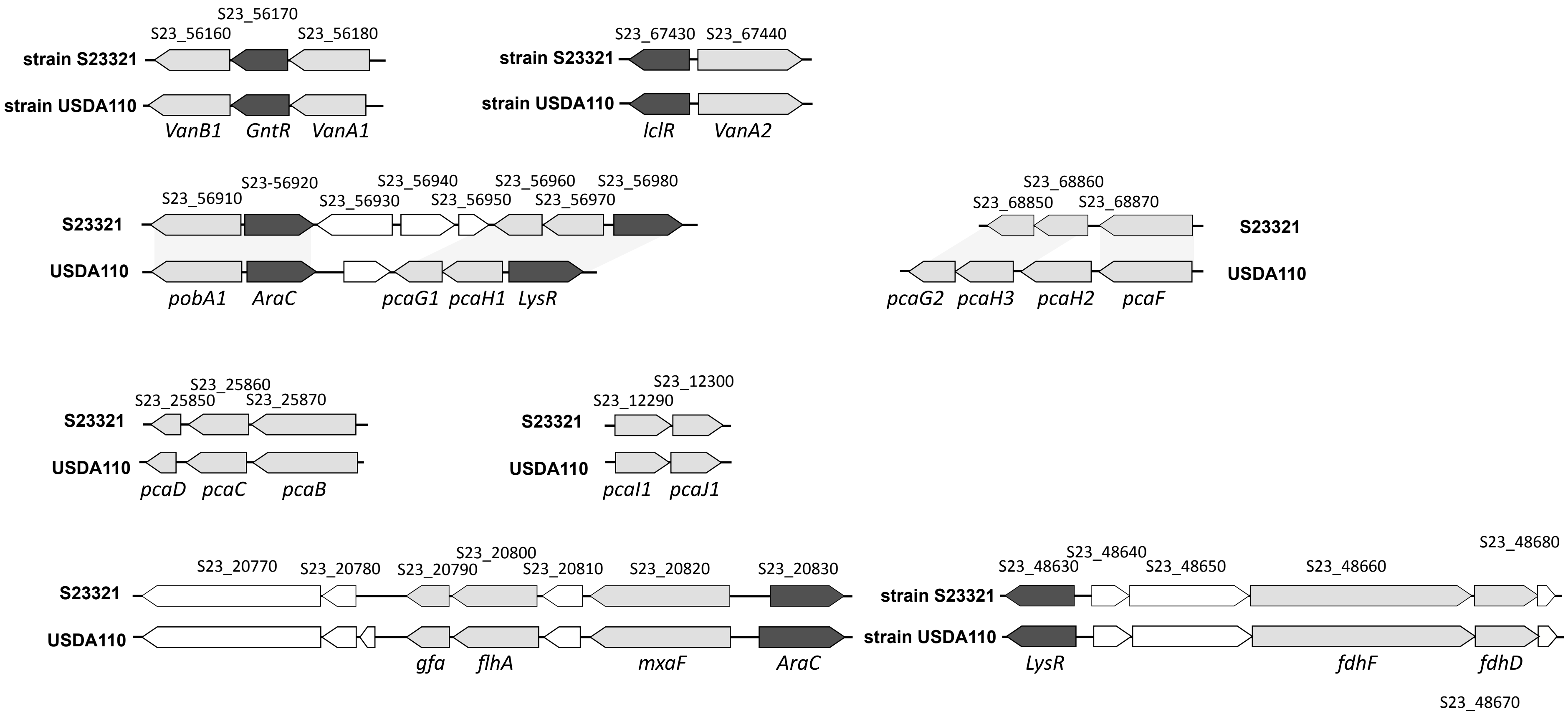

Fig. S7. Organization of genes involved in degrading vanillate, 4-hydroxybenzoate, and formaldehyde in the genomes of *Bradyrhizobium* sp. S23321 and *B. japonicum* USDA110. Gray and black arrows show degradation genes and regulatory genes, respectively.

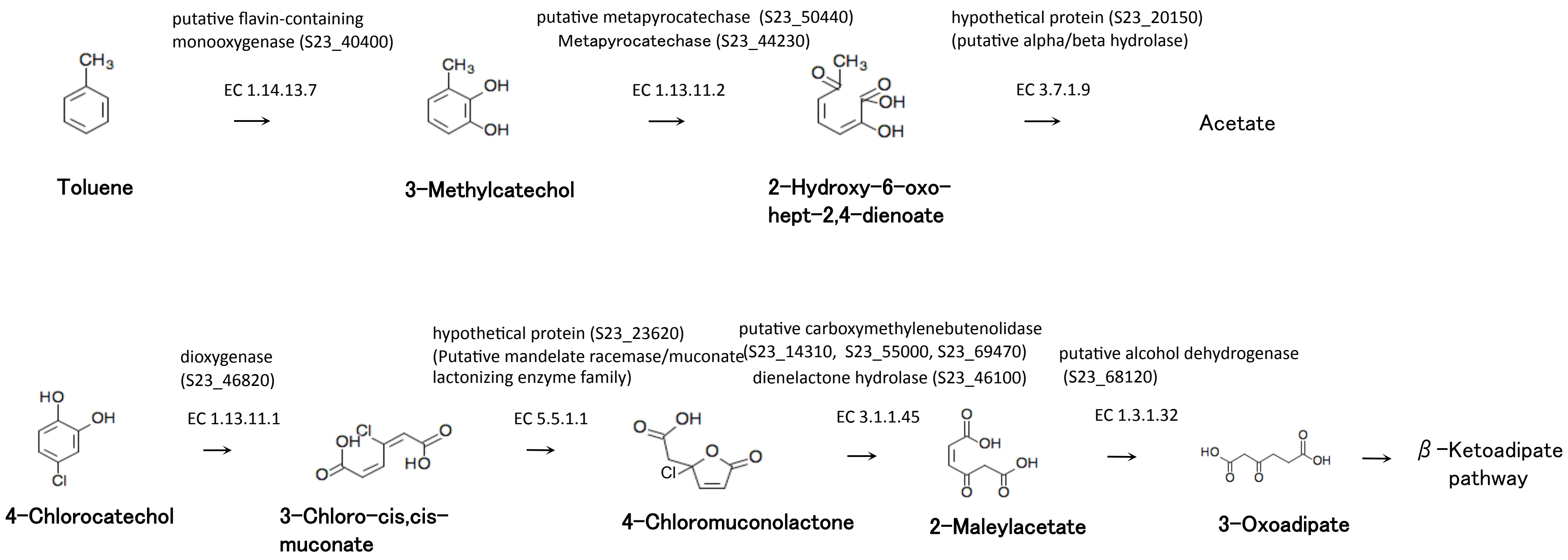

Fig. S8. Pathways and gene homologues from S23321 involved in the degradation of toluene and 4-chlorocatechol.

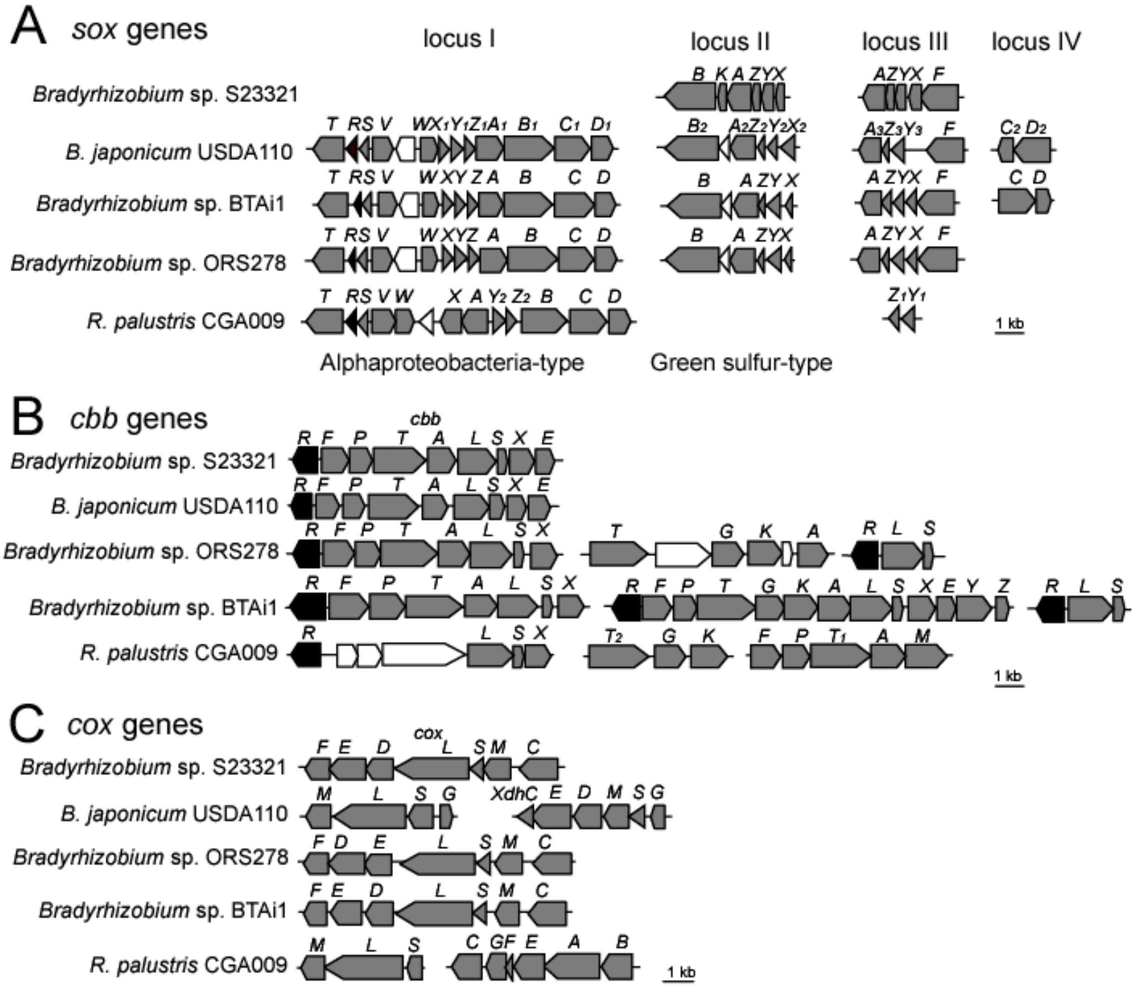

Fig. S9. Physical maps of the genes related to chemoautotrophic growth in the genome of *Bradyrhizobium* sp. S23321 and other members of the *Bradyrhizobiaceae* (*B. japonicum* USDA110, *Bradyrhizobium* sp. BTAi1, *Bradyrhizobium* sp. ORS278, and *Rhodopseudomonas palustris* CGA009). (A) *sox* genes, (B) *cbb* genes, (C) *cox* genes.

Bradyrhizobium sp. S23321 from=3447153, to=3447367 S23_33590

GGCACGCGATTTGCGACCATGCCTGCCAACGACACAGTGAGGATACGATC**ATG**AGAAACATTCTGAAGAA
GTTCATTGCCGACGAATCCGGGGCGACCGCGATCGAATACGGCCTGATCGCCGCCGGCATCGCGCTGGCG
ATCATCACCGTCATCAACGGCATGGGCACCCGGCTCAGCACGAAATTCGGGTCGATCAGCACGTCGCTGAA
GTAG

Bradyrhizobium sp. S23321 from=4124816, to=4124596 S23_40290

CATTTCTCGTGCGCCGGCAACGTCTGGTAGAGTTGGGAAGGACACGGACT**GTG**AAATTGAAATGCCTGTT
GGCCGAGTTTGCCGCCGATGAATCCGGCGCCACCGCGATCGAATACGGCCTGATCGCAGCGGCGATCGCG
CTTGCGATCATCGAAGTGATCTATGCGCTCGGCACCAATCTCGTCGCCAAGCTGCAAGCGCTGGCGACGGC
GCTGAAATAG

Bradyrhizobium sp. S23321 from=4995253, to=4995467 S23_48860

TCCGGAAACGATGCGGCCATGGATTTGGGCCGGATGAACAGGAGTTGGCA**ATG**CGTTTGATTAGGTCTTT
CCTTGCCGATGAGGCTGGCGCCACCGCCATCGAGTACGGCCTGATCGCCGCCGGCATTGCGCTTGCCATCG
TGACCGTCGTGAACAACACGGGCAGCGCGCTCCTCAACAACAAATTCAACTCGATCAGTACGTCGCTGAAG
TAG

Fig. S10. Putative SD (Shine-Dalgarno) sequences (underlined) of the three *pilA* genes in the S23321genome. Start codons of the *pilA* genes are shown in red.

Table S1. Bacterial strains used for phylogenetic analysis.

Strain	Accession no. of DNA sequence			Host plant or source	Relevant characteristics	Reference
	16S rRNA gene	ITS	*nifH*			
Bradyrhizobium japonicum						
USDA110	AP005940	AB100749	BA000040	*Glycine max*	Soybean bradyrhizobia, *hup*$^{+}$, *nos*$^{+}$	18
NC6	AB231918	AB278126	-	*Glycine max*	Soybean bradyrhizobia, *hup*$^{+}$, *nos*$^{+}$	41
USDA122	AF208503	AB100751	AB685466	*Glycine max*	Soybean bradyrhizobia, *hup*$^{+}$, *nos*$^{+}$	50
NK2	AB070567	AB278127	–	*Glycine max*	Soybean bradyrhizobia, *hup*$^{+}$, *nos*$^{+}$	40
NC4	AB231917	AB278125	–	*Glycine max*	Soybean bradyrhizobia, *hup*$^{+}$, *nos*$^{+}$	41
USDA124	AF208505	AB100753	–	*Glycine max*	Soybean bradyrhizobia, *hup*$^{-}$, *nos*$^{-}$	50
USDA6^{T}	U69638	AB100741	AP012206	*Glycine max*	Soybean bradyrhizobia, *hup*$^{-}$, *nos*$^{-}$	50
T7	AB231929	AB278128	–	*Glycine max*	Soybean bradyrhizobia, *hup*$^{-}$, *nos*$^{-}$	41
T9	AB231930	AB278129	–	*Glycine max*	Soybean bradyrhizobia, *hup*$^{-}$, *nos*$^{-}$	41
Agromonas oligotrophica						
S58	D78366	–	–	Rice rhizosphere	Slow-growing oligotrophic bacteria	39
G14130	AB003457	–	–	Pasture soil	Slow-growing oligotrophic bacteria	39
Bradyrhizobium sp.						
BTAi1	D86354	–	CP000494	*Aeschynomene sensitiva*	Photosynthetic stem-nodulating bacteria	13
ORS278	AJ133779	–	CU234118	*Aeschynomene indica*	Photosynthetic stem-nodulating bacteria	13
HW13	D89027	–	–	Hawaiian soil	2,4-D–degrading bacteria[1)]	17
HWK12	D89028	–	–	Hawaiian soil	2,4-D–degrading bacteria[1)]	17
Rhodopseudomonas palustris						
CGA009	BX572607	–	–		Photosynthetic bacteria	20
Bradyrhizobium elkanii						
USDA76^{T}	U35000	–	AB094963	*Glycine max*	Soybean bradyrhizobia	50
Nitrobacter winogradskyi						
Nb-255	CP000115	–	–			
Blastobacter denitrificans						
	AF338176	–	–			

[1)] 2,4-D: 2,4-dichloropheenoxyacetic acid

Table S2. Comparison of flagella genes in several bradyrhizobial genomes

	gene	S23321	*Bradyrhizobium japonicum* USDA110	*Bradyrhizobium* sp. BTAi1	*Bradyrhizobium* sp. ORS278
motor protein	MotB2	S23_10870	bll1510	BBta_6939	BRADO1110
**Lateral flagella proteins**					
two-component response regulator	FlbD	S23_13020	blr7003	BBta_1872	BRADO5903
motor switch protein, C-ring component	FliN	S23_13030	blr7002	BBta_1873	BRADO5902
assembly protein	FliH	S23_13040	blr7001	BBta_1874	BRADO5901
motor switch protein, C-ring component	FliG	S23_13050	blr7000	BBta_1875	BRADO5900
MS-ring protein	FliF	S23_13060	blr6999	BBta_1876	BRADO5899
hook cap protein	FlgD1	S23_13080	blr6997	BBta_1878	BRADO5897
Hook-length control protein	FliK	S23_13090	blr6996	BBta_1880	BRADO5895
Flagellar Protein Export ATPase	FliI	S23_13870	blr6885	-	-
basal body rod protein	FlgF	S23_13880	blr6884	-	-
motor protein	MotA	S23_13900	bll6882	-	-
motor switch protein, C-ring component	FliM	S23_13910	bll6881	-	-
motor switch protein, C-ring component	FliN	S23_13930	bll6879	-	-
motor switch protein, C-ring component	FliG	S23_13940	bll6878	-	-
Export apparatus protein	FlhB	S23_13950	bll6877	-	-
basal body rod protein	FlgB	S23_13960	bll6876	-	-
basal body rod protein	FlgC	S23_13970	bll6875	-	-
basal body rod protein	FliE	S23_13980	bll6874	-	-
basal body rod protein	FlgG	S23_13990	bll6873	-	-
P-ring biosynthesis protein	FlgA	S23_14000	bll6872	-	-
basal body P-ring protein	FlgI	S23_14010	bll6871	-	-
basal body L-ring protein	FlgH	S23_14030	bll6869	-	-
Unknown function	FliL	S23_14040	bll6868	-	-
Export apparatus protein	FliP	S23_14050	bll6867	-	-
Flagellin	FliC1	S23_14060	bll6866	-	-
Flagellin	FliC2	S23_14070	-	-	-
Flagellin	FliC3	S23_14080	bll6865	-	-
MS-ring protein	FliF	S23_14090	bll6864	-	-
motor protein	MotB	S23_14110	bll6862	-	-
motor protein	MotC	S23_14120	bll6861	-	-
motor protein	MotD	S23_14130	bll6860	-	-
hook protein	FlgE	S23_14150	bll6858	-	-
hook-associated protein, HAP1	FlgK	S23_14160	bll6857	-	-
hook-associated protein, HAP3	FlgL	S23_14170	bll6856	-	-
Flagellar assembly protein	FlaF	S23_14180	bll6855	-	-
flagellar biosynthesis repressor	FlbT	S23_14190	bll6854	-	-
Hook cap protein	FlgD2	S23_14200	bll6853	-	-
Export apparatus protein	FliQ	S23_14210	bsl6852	-	-
Export apparatus protein	FlhA	S23_14220	bll6851	-	-
Export apparatus protein	FliR	S23_14230	bll6850	-	-
Unknown function	CheL	S23_14250	bll6849	-	-
flagellin	FliC4	S23_15090	blr3695	BBta_3159	BRADO4890
Flagella cap protein, HAP2	FliD	S23_15110	blr3696	BBta_3160	BRADO4889
Flagellin chaperon	FliS	S23_15120	blr3697	BBta_3161	BRADO4888
Hook cap protein	FlgD	S23_15150	blr3699	BBta_3164	BRADO4885
hook protein FlgE	FlgE	S23_15160	blr3700	BBta_3165	BRADO4884
Unknown function	FlbY	S23_15180	blr3702	BBta_3167	BRADO4882
hook-associated protein, HAP1	FlgK	S23_15190	blr3703	BBta_3168	BRADO4881

hook-associated protein, HAP3	FlgL	S23_15200	blr3704	BBta_3169	BRADO4880
**Polar flagellar proteins**					
biosynthesis repressor	FlbT	S23_24010	blr5847	BBta_5529	BRADO5057
Flagellin, S-layer protein	FliC5	S23_24020	blr5846	BBta_5528	BRADO5056
Flagellin, S-layer protein	FliC6	S23_24030	blr5845	BBta_5527	BRADO5055
biosynthesis regulatory protein	FlaF	S23_24040	bll5842	BBta_5526	BRADO5054
Unknown function	flbY	S23_24070	blr5840	BBta_5522	BRADO5052
Unknown function	cheL	S23_24080	blr5839	BBta_5521	BRADO5051
basal body P-ring protein	FlgI	S23_24090	blr5838	BBta_5520	BRADO5050
assembly regulator FliX	FliX	S23_24100	bll5837	BBta_5519	BRADO5049
basal body L-ring protein	FlgH	S23_24170	blr5830	BBta_5512	BRADO5040
basal body P-ring biosynthesis protein	FlgA	S23_24180	blr5829	BBta_5511	BRADO5039
basal body rod protein	FlgG	S23_24190	blr5828	BBta_5510	BRADO5038
basal body rod protein	FlgF	S23_24200	blr5827	BBta_5509	BRADO5037
basal body-associated protein	FliL	S23_24210	bll5826	BBta_5508	BRADO5036
motor switch protein, C-ring component	FliM	S23_24220	bll5825	BBta_5507	BRADO5035
biosynthesis protein	FliP	S23_24270	blr5816	BBta_5499	BRADO5031
Flagellar protein, unknown function	?	S23_24280	blr5815	BBta_5498	BRADO5030
basal body rod protein	FlgB	S23_24290	bll5814	BBta_5497	BRADO5029
basal body rod protein	FlgC	S23_24300	bll5813	BBta_5496	BRADO5028
basal body rod protein	FliE	S23_24310	bll5812	BBta_5495	BRADO5027
Export apparatus protein	FliQ	S23_24320	bsl5811	BBta_5494	BRADO5026
Export apparatus protein	FliR	S23_24330	bll5810	BBta_5493	BRADO5025
Export apparatus protein	FlhB	S23_24340	bll5809	BBta_5492	BRADO5024
motor switch protein, C-ring component	FliN/FliY	S23_30290	bsl5256	BBta_4891	-
Motor protein	MotB	S23_44480	blr3801	BBta_3511	BRADO4686
Motor protein	MotA	S23_44490	blr3800	BBta_3510	BRADO4687
basal-body rod protein	FlgC	S23_48230	blr3174	-	-
Export apparatus protein	FlhA	S23_58150	bll2207	BBta_6527	BRADO1503
Flagellar assembly protein	FliJ	S23_58210	blr2202	BBta_6531	BRADO1499
Flagellar Protein Export ATPase	FliI	S23_58220	blr2201	BBta_6532	BRADO1498

-, no related gene

Table S3. List of genes in S23321 found on the symbiosis islands of the USDA110 genome

S23321 ID	start	stop	direction	amino acid length	product	USDA110 ID	start	stop	direction	amino acid length	product	gene name
S23_05150	528701	529270	-	189	hypothetical protein	bll1918	2074835	2075341	-	168	unknown protein	
S23_11190	1187785	1188234	+	149	4-hydroxybenzoyl-CoA thioesterase	bll7211	7933983	7934315	-	110	hypothetical protein	
S23_14720	1548869	1549696	-	275	binding-protein-dependent transport systems inner membrane component	blr1891	2044742	2045602	+	286	ABC transporter permease protein	
S23_19180	2014402	2015397	+	331	chlorophyllide reductase iron protein subunit X	blr1769	1928567	1929451	+	294	dinitrogenase reductase protein	
S23_19410	2040555	2041484	+	309	protochlorophyllide reductase iron-sulfur ATP-binding protein	blr1769	1928567	1929451	+	294	dinitrogenase reductase protein	
S23_20050	2105070	2105729	+	219	putative polysaccharide deacetylase	blr2026	2184759	2185418	+	219	nodulation chito-oligosaccharide deacetylase	
S23_24510	2585368	2586978	-	536	histidine kinase	blr2178	2353736	2355418	+	560	two-component hybrid sensor and regulator	
S23_26740	2804045	2805742	+	565	hypothetical protein	blr1611	1767237	1768103	+	288	unknown protein	
S23_36010	3697746	3698051	+	101	putative transposase	bll1823	1974713	1975336	-	207	putative transposase	
S23_39360	4018369	4018653	+	94	hypothetical protein	bll1559	1700812	1701195	-	127	unknown protein	
S23_40490	4138880	4140991	+	703	putative sensor histidine kinase with two PAS and a response regulator receiver domains	bll2176	2350024	2351928	-	634	two-component hybrid sensor and regulator	
S23_43830	4474580	4475320	-	246	hypothetical protein	bll2012	2173171	2174097	-	308	hypothetical protein	
S23_45840*	4675510	4676325	+	271	probable phosphotransferase	blr1894	2047300	2048127	+	275	probable phosphotransferase	
S23_45910*	4681468	4682022	+	184	thioredoxin peroxidase	bll1777	1935851	1936405	-	184	alkyl hydroperoxide reductase	
S23_45920*	4682031	4682576	+	181	alkylhydroperoxidase	bll1776	1935296	1935844	-	182	alkyl hydroperoxide reductase	
S23_45930*	4682626	4682937	-	103	putative ferredoxin protein	bsr1775	1934788	1935084	+	98	probable ferredoxin	fixX
S23_45940*	4682961	4684268	-	435	FixC protein	blr1774	1933442	1934749	+	435	flavoprotein	fixC
S23_45950*	4684282	4685391	-	369	protein FixB	blr1773	1932321	1933430	+	369	electron transfer flavoprotein alpha chain	fixB
S23_45960*	4685411	4686253	-	280	electron transfer flavoprotein beta-subunit FixA	blr2038	2198527	2199393	+	288	electron transfer flavoprotein beta chain	fixA
S23_45970*	4686509	4686838	-	109	nitrogenase-stabilizing/protective protein NifW	blr1771	1931029	1931370	+	113	nitrogenase stabilizing/protective protein	nifW
S23_46010*	4689477	4690268	-	263	putative nitrogen fixation protein NifQ	blr1770	1929556	1930374	+	272	molybdenum processing protein	nifQ
S23_46020*	4690275	4691165	-	296	nitrogenase iron protein	blr1769	1928567	1929451	+	294	dinitrogenase reductase protein	nifH
S23_46040*	4692005	4692430	+	141	putative arsenate reductase	bll1767	1927574	1927999	-	141	hypothetical protein	
S23_46050*	4692427	4692783	-	118	2Fe-2S ferredoxin	blr1765	1925868	1926215	+	115	ferredoxin	
S23_46130*	4696274	4696723	-	149	hypothetical protein	blr1753	1917810	1918193	+	127	hypothetical protein	
S23_46200*	4699427	4699744	-	105	nitrogen fixation protein NifZ	blr1761	1923595	1924158	+	187	iron-sulfur cofactor synthesis protein	nifZ
S23_46230*	4701057	4701365	-	102	putative iron-sulfur cluster assembly protein	blr1755	1919065	1919385	+	106	R. etli iscN homolog	
S23_46240*	4701379	4701603	-	74	ferredoxin-like protein	bsr1760	1923369	1923593	+	74	ferredoxin-like protein	frxA
S23_46250*	4701618	4703174	-	518	nitrogenase FeMo cofactor biosynthesis protein NifB	blr1759	1921859	1923358	+	499	FeMo cofactor biosynthesis protein	nifB
S23_46270*	4704344	4704568	-	74	FixU nitrogen fixation protein	bsr1757	1920887	1921111	+	74	nitrogen fixation protein	fixU
S23_46280*	4704565	4705758	-	397	cysteine desulfurase	blr1756	1919709	1920890	+	393	nitrogenase metalloclusters biosynthesis protein	nifS
S23_46300*	4706119	4706439	-	106	iron-binding protein IscA	blr1755	1919065	1919385	+	106	R. etli iscN homolog	
S23_46310*	4706788	4707378	+	196	hypothetical protein	bll1754	1918215	1918934	-	239	hypothetical protein	
S23_46320*	4707390	4707794	-	134	hypothetical protein	blr1753	1917810	1918193	+	127	hypothetical protein	
S23_46360*	4709223	4709504	-	93	ferredoxin-3	bsr1750	1915192	1915485	+	97	ferredoxin	
S23_46370*	4709517	4709717	-	66	hypothetical protein	bsr1749	1915020	1915181	+	53	hypothetical protein	
S23_46380*	4709726	4710196	-	156	hypothetical protein	blr1748	1914503	1914967	+	154	hypothetical protein	
S23_46390*	4710208	4710603	-	131	nitrogenase molybdenum-iron protein NifX	blr1747	1914101	1914496	+	131	iron-molibdenum cofactor processing protein	nifX
S23_46400*	4710624	4712006	-	460	nitrogenase reductase-associated ferredoxin NifN	blr1746	1912695	1914104	+	469	nitrogenase molybdenum-cofactor synthesis protein	nifN
S23_46410*	4712015	4713685	-	556	nitrogenase MoFe cofactor biosynthesis protein NifE	blr1745	1911042	1912685	+	547	nitrogenase molybdenum-cofactor synthesis protein	nifE
S23_46420*	4713805	4715364	-	519	nitrogenase molybdenum-iron protein beta chain	blr1744	1909393	1910949	+	518	nitrogenase molybdenum-iron protein beta chain	nifK
S23_46430*	4715443	4716945	-	500	nitrogenase molybdenum-iron protein alpha chain	blr1743	1907825	1909327	+	500	nitrogenase molybdenum-iron protein alpha chain	nifD
S23_46440*	4717022	4717912	-	296	nitrogenase iron protein	blr1769	1928567	1929451	+	294	dinitrogenase reductase protein	
S23_46530*	4724654	4724848	-	64	ferredoxin	bsr1739	1904611	1904805	+	64	ferredoxin	
S23_46540*	4724996	4726732	-	578	NifA subfamily transcriptional regulatory protein	blr2037	2196192	2198009	+	605	nif-specific regulatory protein	nifA
S23_46550*	4726979	4727512	-	177	FixR protein	blr2036	2195172	2196008	+	278	oxidoreductase	fixR
S23_49180	5026962	5027420	+	152	hypothetical protein	blr2041	2200533	2201756	+	407	unknown protein	
S23_55210	5692088	5692654	+	188	hypothetical protein	blr2006	2166817	2167335	+	172	unknown protein	
S23_55250	5695481	5695786	+	101	putative transposase	bll1823	1974713	1975336	-	207	putative transposase	
S23_55430	5714015	5714758	+	247	3-oxoacyl-(acyl-carrier-protein) reductase	blr2167	2341344	2342093	+	249	oxidoreductase	
S23_56360	5812978	5818554	-	1858	amino acid adenylation domain protein	blr2108	2276848	2286780	+	3310	probable peptide synthetase	
S23_58440	6042128	6043072	+	314	hypothetical protein	bll1944	2106063	2108540	-	825	hypothetical protein	
S23_62670	6486937	6490842	-	1301	hypothetical protein	blr2108	2276848	2286780	+	3310	probable peptide synthetase	
S23_62680	6490862	6498976	-	2704	amino acid adenylation domain-containing protein	blr2108	2276848	2286780	+	3310	probable peptide synthetase	
S23_62690	6498973	6500913	-	646	amino acid adenylation domain-containing protein	blr2108	2276848	2286780	+	3310	probable peptide synthetase	
S23_62880	6521652	6528113	+	2153	amino acid adenylation domain protein	blr2108	2276848	2286780	+	3310	probable peptide synthetase	
S23_64700	6714414	6716468	-	684	putative two-component sensor histidine kinase transcriptional regulatory protein	blr2178	2353736	2355418	+	560	two-component hybrid sensor and regulator	
S23_64710	6716465	6717964	-	499	hypothetical protein	blr2177	2352229	2353749	+	506	hypothetical protein	
S23_65150	6763253	6765313	+	686	PAS sensor protein	blr2178	2353736	2355418	+	560	two-component hybrid sensor and regulator	
S23_65660	6808688	6810937	-	749	putative outer membrane protein	bll1944	2106063	2108540	-	825	hypothetical protein	
S23_66040	6850354	6850734	-	126	putative transposase	blr2053	2214422	2214727	+	101	putative transposase	

Table S4. Comparison of genes related to stress response in S23321 and its relatives

	S23321	*Bradyrhizobium japonicum* USDA110	*Bradyrhizobium* sp. BTAi1	*Bradyrhizobium* sp. ORS278	*Rhodopseudomonas palustris* CGA009
**Oxidative stress**					
catalase-peroxidase (*katG*)	S23_00520	blr0778	BBta_6389	BRADO1666	RPA0429
Fe/Mn containing superoxide dismutase (*sodB*)	S23_68490	bll7774	BBta_1335	BRADO6273	RPA1693
Fe/Mn containing superoxide dismutase (*chrC*)	-	bll7559	-	-	-
Cu-Zn containing superoxide dismutase (*sodC*)	-	-	-	-	RPA0225*
organic hydroperoxide resistance protein	S23_01000	bll0735	BBta_0109	BRADO0102	RPA0829
thioredoxin peroxidase	S23_29710	blr5308	BBta_4916	BRADO3235	RPA2443
thioredoxin reductase	S23_59830	blr7381	BBta_6803	BRADO1321	RPA4075
thioredoxin reductase	S23_65440	blr1248	BBta_0894	BRADO6640	RPA4536
peroxiredoxin	S23_64840	bll1317	BBta_0951	BRADO6585	RPA4268
glutathione peroxidase	S23_15810	bll6732	BBta_6295	BRADO5788	RPA3627*
glutathione reductase	S23_44920	blr3757	BBta_3485	BRADO4712	RPA1983*
catalase	S23_39650	-	-	-	RPA3310
**Heat shock / chaperone**					
60kDa chaperonin (*groEL1*)	S23_49080	blr5227	BBta_3034	BRADO2697	-
10kDa chaperonin (*groES1*)	S23_49090	blr5226	BBta_3033	BRADO2696	-

60kDa chaperonin (*groEL2*)	-	blr6979	-	-	-
10kDa chaperonin (*groES2*)	-	blr6978	-	-	-
60kDa chaperonin (*groEL3*)	-	bll2059	-	-	-
10kDa chaperonin (*groES3*)	-	bll2060	-	-	-
60kDa chaperonin (*groEL4*)	S23_26250	blr5626	BBta_5285	BRADO2892	RPA2164
10kDa chaperonin (*groES4*)	S23_26260	blr5625	BBta_5284	BRADO2893	RPA2165
60kDa chaperonin (*groEL5*)	S23_61650	blr7533	BBta_1671	BRADO6115	RPA1140
10kDa chaperonin (*groES5*)	S23_61640	bsr7532	BBta_1672	BRADO6114	RPA1141
60kDa chaperonin (*groEL*)	-	blr4635	-	-	-
60kDa chaperonin (*groEL*)	-	blr3683	BBta_3636	BRADO4344	-
heat shock protein 90 (*htpG*)	S23_63530	bll7789	BBta_1326	BRADO6296	RPA4815
heat shock protein 70 (*dnaK*)	S23_01580	blr0678	BBta_0191	BRADO0164	RPA0333
Hsp70 family molecular chaperone	S23_48490	blr3152	-	-	-
heat shock protein 40 (*dnaJ*)	S23_01560	blr0680	BBta_0192	BRADO0163	RPA0334
heat shock protein 40 (*dnaJ*)	S23_53380	blr2626	BBta_2465	BRADO2148	RPA1198
heat shock protein 33 (*hslO*)	S23_67190	blr1100	BBta_0761	BRADO6776	PRA4771
HspC2 heat shock protein	S23_17570	blr7961	BBta_3527*	BRADO4666*	-
heat shock protein Hsp20	S23_49060	-	-	-	-
cytoplasmic chaperone (*torD*)	S23_13140	-	-	-	-
**Cold shock**					
cold shock protein (*cspA*)	S23_10090	bsl1445	BBta_7022	BRADO1028	RPA9073
cold shock protein (*cspA*)	S23_42620	bsl3986	-	-	RPA3672
cold shock protein (*cspA*)	S23_41350	bsl4127	BBta_3854	BRADO3346	RPA3053

cold shock protein (*cspA*)	S23_48460	bsr3154	BBra_7552	BRADO0631	RPA3399
cold shock protein (*cspA*)	S23_58830	bsr7259	BBta_6679	BRADO1429	-
cold shock protein (*cspA*)	S23_37850	blr4410	BBta_4038	BRADO3673	RPA2525
cold shock protein (*cspA*)	S23_35160	bsl4595	BBta_7022	BRADO1028	-
cold shock protein (*cspA*)	S23_60990	bsr7468	BBta_1730	BRADO6053	RPA1173
cold shock dead-box protein A (*csdA*)	S23_26940	bll5562	BBta_4919*	BRADO3233*	RPA4001*
**Osmotic stress**					
betaine aldehyde dehydrogenase	S23_62250*	blr3776*	BBta_3841*	-	RPA0112*
choline dehydrogenase (*cdh*) / choline oxidase (*cox*)	-	-	BBta_6705	BRADO1398	-
glycine sarcosine methyltransferase (*gsm*) / sarcosine dimethylglycine methyltransferase (*sdm*)	-	-	-	-	-
**Desiccation**					
Trehalose-6-phosphate synthetase (*otsA*)	S23_05080	bll0322	BBta_0578	BRADO6952	RPA4660
Trehalose-6-phosphate phosphatase (*otsB*)	S23_05070	bll0323	BBta_0577	BRADO6953	RPA4661
Trehalose synthase (*treS*)	S23_15460	blr6767	BBta_6322*	BRADO5816*	RPA3643*
Maltooligosyltrehalose synthase (*treY*)	S23_15420	blr6771	BBta_6326*	BRADO5820*	RPA3647*

**pH-adaptation**					
pH-adaptive potassium efflux system component (*phaA*)	-	-	-	-	RPA2789
pH-adaptive potassium efflux system component (*phaC*)	-	-	-	-	RPA2790
pH-adaptive potassium efflux system component (*phaD*)	-	-	-	-	RPA2791
pH-adaptive potassium efflux system component (*phaE*)	-	-	-	-	RPA2792
pH-adaptive potassium efflux system component (*phaF*)	-	-	-	-	RPA2793
pH-adaptive potassium efflux system component (*phaG*)	-	-	-	-	RPA2794
**Organic solvents**					
organic solvent tolerance protein	S23_41570*	bll4105*	BBta_3829*	BRADO3325	RPA3063*
**Arsenic / cyanate toxicity**					
arsenate reductase (*arsC*)	S23_49150	bll3086*	BBta_5577*	BRADO5106*	RPA3555
arsenite efflux pump	S23_49160	bll3085	BBta_5576*	-	RPA3553
arsenate reductase (*arsC*)	S23_49170	bll3084	-	-	RPA3554
arsenate reductase (*arsC*)	-	-	BBta_4662	BRADO4444	RPA2257
arsenical pump membrane protein	-	-	-	-	RPA2258

arsenical pump membrane protein (*arsB*)	S23_30120	blr5272	Bbta_4911*	BRADO3240*	RPA3398
Cyanate hydratase (*cynS*)	S23_25190	bll5731	Bbta_5412	BRADO2774	RPA2115*
Cyanate transporter (*cynX*)	-	-	-	-	-
**Regulated degradative capacity**					
clpA	S23_30570	bll5153	BBta_4772	BRADO4545	RPA3147
clpB	S23_63890	blr1404	BBta_1195	BRADO6450	RPA4433
lon	S23_32420	bll4942	BBta_4570	BRADO4196	RPA2959
lon	S23_21210	blr6174	BBta_7794	-	-
**DNA repair**					
recA	S23_24880	bll5755	BBta_5453	BRADO4985	RPA3851
single-strand DNA binding protein RecBCD nuclease (*ssb*)	S23_34570	bll4698	BBta_4373	BRADO4002	RPA2814
recG	S23_35130	blr4603	BBta_4159	BRADO3769	RPA2662
recF	S23_00030	bll0827	BBta_0004	BRADO0003	RPA0003
recJ	S23_39020	blr4377	BBta_4000*	BRADO3577*	RPA2508
recO	S23_31530	bll5058	BBta_4680*	BRADO4460*	RPA2700
recQ	S23_05650	bll0242	BBta_6924	BRADO1125	RPA4826
ruvA	S23_11140	blr1536	BBta_6908	BRADO1145	RPA1100
ruvB	S23_11160	blr1537	BBta_6906	BRADO1147	RPA1102
uvrA	S23_34520	blr4702	BBta_4379	BRADO4010	RPA2816
uvrB	S23_60490	blr7431	BBta_1782	BRADO5995	RPA4343

uvrC	S23_61010	blr7470	BBta_1728	BRADO6055	RPA1171
uvrD	S23_28240	blr5493	BBta_7513*	BRADO0671*	RPA2802*
mutS	S23_68880	bll0924	BBta_7642	BRADO0537	RPA0512
mutL	S23_61250	blr7493	BBta_1703	BRADO6079	RPA4371
mutH	-	-	-	-	-
Dps family DNA-binding stress response protein	S23_59760	bll7374	BBta_6797*	BRADO1329*	RPA1274
**<u>Storage compounds</u>**					
poly-3-hydroxybutyrate synthase (*phbC*)	S23_39220	bll4360	BBta_3992	BRADO3568	RPA2501
poly-3-hydroxybutyrate synthase (*phbC*)	S23_22030	bll6073	BBta_5757*	BRADO5310*	RPA4243*
acetoacetyl CoA reductase (*phbB*)	S23_05770	bll0225	BBta_7613	BRADO0563	RPA0532
acetyl-CoA acetyltransferase	S23_05760	bll0226	BBta_7614	BRADO0562	RPA0531
PHB depolymerase	S23_69010*	blr0908*	BBta_7656*	BRADO0519*	RPA0575*
polyphosphate kinase	S23_41400	bll4122	BBta_3849*	BRADO3341*	RPA3048*
Ppx/GppA family phosphatase	S23_41410*	bll4121*	BBta_3848*	BRADO3340*	RPA3047*
glucose-1-phosphate adenylyltransferease	S23_18760	blr6458	BBta_0922	BRADO6613	RPA0381
glycogen synthase	S23_18750	blr6459	BBta_0923	BRADO6612	RPA0382*
glycogen synthase	S23_52210	bll2778	BBta_2800	BRADO2453	-
glycogen debranching enzyme	S23_15440	blr6769	BBta_6324	BRADO5818	RPA3645
glycogen debranching enzyme	S23_50600	-	BBta_5568	BRADO5096	RPA3311

<u>**Sigma factors**</u>					
sigma 70 (*rpoD*)	S23_59500	bll7349	BBta_6775	BRADO1354	RPA1288
sigma 54 (*rpoN*)	S23_01100	blr0723	BBta_0121	BRADO0113	RPA0050
sigma 54 (*rpoN*)	-	blr1883	-	-	-
sigma 32 (*rpoH*)	S23_59360	blr7337	BBta_6760	BRADO1371	RPA0367
sigma 24 (*rpoE*)	S23_63580	blr7797	BBta_7003	BRADO1047	RPA4225*
ECF sigma	S23_49620	blr3038	BBta_3007*	BRADO2666*	RPA1813*
ECF sigma	S23_15080*	blr3694*	BBta_3158*	BRADO4891*	RPA0639*
ECF sigma	S23_49550*	blr3042*	BBta_3011*	BRADO2670*	RPA1819*
ECF sigma	S23_67780	bll1028	BBta_0709	BRADO6834	RPA4792*
ECF sigma	S23_58200*	blr2203*	BBta_6530	BRADO1500	RPA1635*
ECF sigma	S23_18500	bll6484	-	-	-
ECF sigma	S23_53360	bll2628	BBta_2467	BRADO2150	-
ECF sigma	S23_49840	bll3014*	BBta_2977*	BRADO2637*	-
ECF sigma	-	blr4928	-	-	-

*, gene definition is “putative”.

-, no related gene

Table S5. Genes for respiratory chains in the S23321 genome.

Complex I	ID	Gene name	Product	Closest gene	AA Identity (%)
NADH dehydrogenase	S23_32620	*nuoA*	NADP ubiquinone oxidoreductase chain A	NADP ubiquinone oxidoreductase chain A (bll4919) of *B. japonicum* USDA110	92.6
	S23_32630	*nuoB*	NADP ubiquinone oxidoreductase chain B	NADP ubiquinone oxidoreductase chain B (bll4918) of *B. japonicum* USDA110	95.4
	S23_32640	*nuoC*	NADP ubiquinone oxidoreductase chain C	NADP ubiquinone oxidoreductase chain C (bll4917) of *B. japonicum* USDA110	88.7
	S23_32650	*nuoD*	NADP ubiquinone oxidoreductase chain D	NADP ubiquinone oxidoreductase chain D (bll4916) of *B. japonicum* USDA110	94.5
	S23_32670		NADH-ubiquinone oxidoreductase chain E	NADP ubiquinone oxidoreductase chain E (bll4914) of *B. japonicum* USDA110	95.6
	S23_32690	*nuoF*	NADP ubiquinone oxidoreductase chain F	NADP ubiquinone oxidoreductase chain F (bll4912) of *B. japonicum* USDA110	95.5
	S23_32700	*nuoG*	NADP ubiquinone oxidoreductase chain G	NADP ubiquinone oxidoreductase chain G (bll4911) of *B. japonicum* USDA110	92.6
	S23_32710	*nuoH*	NADP ubiquinone oxidoreductase chain H	NADP ubiquinone oxidoreductase chain H (bll4910) of *B. japonicum* USDA110	88.7
	S23_32720	*nuoI*	NADP ubiquinone oxidoreductase chain I	NADP ubiquinone oxidoreductase chain I (bll4909) of *B. japonicum* USDA110	96.3
	S23_32730	*nuoJ*	NADP ubiquinone oxidoreductase chain J	NADP ubiquinone oxidoreductase chain J (bll4908) of *B. japonicum* USDA110	86.3
	S23_32740	*nuoK*	NADP ubiquinone oxidoreductase chain K	NADP ubiquinone oxidoreductase chain K (bll4907) of *B. japonicum* USDA110	88.2
	S23_32750	*nuoL*	NADP ubiquinone oxidoreductase chain L	NADP ubiquinone oxidoreductase chain L (bll4906) of *B. japonicum* USDA110	86.7
	S23_32760	*nuoM*	NADP ubiquinone oxidoreductase chain M	NADP ubiquinone oxidoreductase chain M (bll4905) of *B. japonicum* USDA110	85.9
	S23_32770	*nuoN*	NADP ubiquinone oxidoreductase chain N	NADP ubiquinone oxidoreductase chain N (bll4904) of *B. japonicum* USDA110	87.7

Complex II	ID	Gene name	Product	Closest gene	AA Identity (%)
Succinate dehydrogenase	S23_03230	*sdhB*	succinate dehydrogenase iron-sulfur subunit	succinate dehydrogenase iron-sulfur protein subunit (blr0515) of *B. japonicum* USD	96.2
	S23_03240	*sdhA*	succinate dehydrogenase flaboprotein subunit	succinate dehydrogenase flaboprotein subunit (blr0514) of *B. japonicum* USDA110	94.6
	S23_03250	*sdhD*	succinate dehydrogenase hydrophobic membrane anchor protein su	succinate dehydrogenase hydrophobic membrane anchor protein subunit (blr0513)	85.8
	S23_03260	*sdhC*	succinate dehydrogenase cytochrome B-556 subunit	succinate dehydrogenase hydrophobic membrane anchor protein subunit (blr0512)	81.7

Complex III	ID	Gene name	Product	Closest gene	AA Identity (%)
cytochrome *bc*$_1$ complex	S23_55010	*fbcH*	cytochrome b/c1 precursor	cytochrome b/c1 precursor (blr2486) of *B. japonicum* USDA110	89.5
	S23_55020	*fbcF*	ubiquinol-cytochrome c reductase iron-sulfur subunit	rieske iron-sulfur protein (blr2485) of *B. japonicum* USDA110	96.4

Complex IV	ID	Gene name	Product	Closest gene	AA Identity (%)
heme-copper quinol oxidase	S23_06500	*cyoD*	cytochrome O ubiquinol oxidase subunit IV	*cyoD*-like (blr0152) of *B. japonicum* USDA110	88.1
	S23_06510	*cyoC*	cytochrome O ubiquinol oxidase subunit III	*cyoC*-like (blr0151) of *B. japonicum* USDA110	88.0
	S23_06520	*cyoB*	cytochrome O ubiquinol oxidase subunit I	*cyoB*-like (blr0150) of *B. japonicum* USDA110	92.3
	S23_06530	*cyoA*	cytochrome O ubiquinol oxidase subunit II	*cyoA*-like (blr0149) of *B. japonicum* USDA110	91.2
aa$_3$-type heme-copper cytochrome c oxidase (Bott et al. 1990)	S23_07910	*coxA*	cytochrome *C* oxidase subunit I	*coxA* (blr1171) of *B. japonicum* USDA110	92.1
	S23_07900	*coxB*	cytochrome *c* oxidase subunit II	*coxB* (blr1170) of *B. japonicum* USDA110	89.1
	S23_07950	*coxC*	cytochrome *C* oxidase subunit III	*coxC* (blr1175) of *B. japonicum* USDA110	93.9
	S23_07930	*coxF*	CoxF protein	*coxF* (bsr1173) of *B. japonicum* USDA110	89.7
bd-type quinol oxidase (Arslan 2001)	S23_13360		putative quinol oxidase subunit I transmembrane protein	putative quinol oxidase subunit I transmembrane protein (bll0283)	90.0
	S23_13370		putative quinol oxidase subunit II transmembrane protein	putative quinol oxidase subunit II transmembrane protein (bll0282)	77.7
heme-copper cytochrome oxidase (Gottfert et al. 2005)	S23_37010		putative cytochrome-*c* oxidase subunit II	hypothetical protein (bll4481) of *B. japonicum* USDA110	87.2
	S23_37020		putative cytochrome-*c* oxidase subunit I	probable cytochrome c oxidase polypeptide I (bll4480) of *B. Japonicum* USDA110	88.7
	S23_37030		cytochrome C4	probable cytochrome c4 (bll4479) of *B. japonicum* USDA110	87.8
	S23_36980		putative cytochrome C4	putative cytochrome c4 (bll4483) of *B. japonicum* USDA110	93.1
	S23_36990		putative alcohol dehydrogenase cytochrome C subunit precursor	putative alcohol dehydrogense cytochrome C subunit precursor (bll4482)	91.8
cbb$_3$-type heme-copper cytochrome oxidase (Preisig et al. 1993)	S23_52360	*fixN*	cytochrome-c oxidase	*fixN* (blr2763) of *B. japonicum* USDA110	90.0
	S23_52350	*fixO*	cytochrome-c oxidase	*fixO* (blr2764) of *B. japonicum* USDA110	91.0
	S23_52340	*fixQ*	cbb3 oxidase subunit IV	*fixQ* (blr2765) of *B. japonicum* USDA110	95.0
	S23_52330	*fixP*	cytochrome-c oxidase FixP chain	*fixP* (blr2766) of *B. japonicum* USDA110	91.4
bb$_3$-type heme-copper quinol oxidase (Surpin et al. 1996)	S23_52770	*coxZ*	cytochrome o ubiquinol oxidase subunit IV	*coxZ* (blr2717) of *B. japonicum* USDA110	77.5
	S23_52780	*coxY*	cytochrome o ubiquinol oxidase subunit III	*coxY* (blr2716) of *B. japonicum* USDA110	89.7
	S23_52790	*coxX*	putative cytochrome o ubiquinol oxidase chain I protein	*coxX* (blr2715) of *B. japonicum* USDA110	89.8
	S23_52800	*coxW*	cytochrome o ubiquinol oxidase subunit II	*coxW* (blr2714) of *B. japonicum* USDA110	90.6

Complex V	ID	Gene name	Product	Closest gene	AA Identity (%)
FoF1 ATP synthase	S23_04000	*atpH*	ATP synthase subunit delta	ATP synthase delta chain (bll0443) of *B. japonicum* USDA110	89.2
	S23_04010	*atpA*	ATP synthase subunit alpha	ATP synthase alpha chain (bll0442) of *B. japonicum* USDA110	91.7
	S23_04020	*atpG*	ATP synthase gamma chain	ATP synthase gamma chain (bll0441) of *B. japonicum* USDA110	85.6
	S23_04030		ATP synthase beta chain	ATP synthase beta chain (bll0440) of *B. japonicum* USDA110	93.3
	S23_04050	*atpC*	ATP synthase epsilon chain	ATP synthase epsilon chain (bll0439) of *B. japonicum* USDA110	88.1
	S23_08050		ATP synthase subunit b	FoF1 ATP synthase B chain (bll1185) of *B. japonicum* USDA110	89.4
	S23_08060		ATP synthase subunit b/b'	FoF1 ATP synthase B' chain (bll1186) of *B. japonicum* USDA110	88.3
	S23_08070	*atpC*	F0F1 ATP synthase subunit C	FoF1 ATP synthase C chain (bll1187) of *B. japonicum* USDA110	90.8
	S23_08080		ATP synthase subunit a	FoF1 ATP synthase A chain (bll1188) of *B. japonicum* USDA110	89.2
	S23_08090		ATP synthase subunit A'	FoF1 ATP synthase subunit I (bsl1189) of B. japonicum USDA110	95.4

Table S6. Genes for ABC (ATP-binding cassette) proteins and their interacting partners in the *Bradyrhizobium* sp. S23321 genome.

	Family *	Function	Component	Gene name	Missed compo nent	Predicted transport substrate	Orthologues** in			
							Bradyrhizobium japonicum USDA110	other Rhizobiaceae		other bacteria
1	MOS	importer	BP(S23_00190)		IM, ABC	ribose	-	mlr5740	*Mesorhizobium loti* MAFF303099	
2		excinuclease	subunit C(S23_00980)		subunit A, B	-	-	RL0110	*Rhizobium leguminosarum* bv. *visiae* 3841	
3	OSP	importer	BP(S23_01020)<br>IM(S23_01030)<br>IM(S23_01040)<br>ABC(S23_01050)			glycerol-3 phosphate	BP(bll0733)<br>IM(bll0732)<br>IM(bll0731)<br>ABC(bll0730)			
4	o228	exporter	OMR(S23_03790)<br>IM(S23_03800)<br>ABC(S23_03810)				IM(bll0462)<br>IM(bll0461)<br>ABC(bll0460)			
5	OTCN	importer	BP(S23_04540)<br>IM(S23_04560)<br>ABC(S23_04550)				BP(bll0381)<br>IM(bll0379)<br>ABC(bll0380)			
6	HAA	importer	BP(S23_06050)<br>IM(S23_06040)<br>IM(S23_06060)<br>ABC(S23_06070)<br>ABC(S23_06080)				BP(bll0196)<br>IM(bll0197)<br>IM(bll0195)<br>ABC(bll0194)<br>ABC(bll0193)			
7	OTCN	importer	BP(S23_06990)<br>IM(S23_07010)<br>ABC(S23_0700)				BP(bll0113)<br>IM(bll0111)<br>ABC(bll0112)			

8	OTCN	importer	BP(S23_07240)<br>IM(S23_07260)<br>ABC(S23_07250)				BP(bll0091)<br>IM(bll0089)<br>ABC(bll0090)				
9	MOI	importer	BP(S23_07340)<br>IM(S23_07350)<br>ABC(S23_07360)	*modABC*		molybdenum	BP(bll8162)<br>IM(bll8161)<br>ABC(bll8160)				
10	OPN	importer	BP(S23_08330)<br>IM(S23_08310)<br>IM(S23_08320)<br>ABC(S23_08300)				BP(bll1212)<br>IM(bll1210)<br>IM(bll1211)<br>ABC(bll1209)				
11	DRI	unknown	ABC(S23_08510)		IM		ABC(blr7933)				
12	HAA	importer	IM(S23_08520)<br>IM(S23_08530)<br>ABC(S23_08540)<br>ABC(S23_08550)		BP		IM(bll7932)<br>IM(bll7931)<br>ABC(bll7930)<br>ABC(bll7929)				
13	OPN	importer	BP(S23_08630)<br>BP(S23_08640)<br>IM(S23_08650)<br>IM(S23_08660)<br>ABC(S23_08670)<br>ABC(S23_08700)				BP(blr7922)<br>BP(bll7921)<br>IM(bll7920)<br>IM(bll7919)<br>ABC(bll7918)<br>ABC(bll7916)				
14	DPL	exporter	IM+ABC(S23_08720)				IM+ABC(bll7914)				
15	DRI	exporter	IM(S23_09110)<br>IM+ABC+ABC(S23_09120)				IM(blr7874)<br>IM+ABC+ABC(blr7873)				
16	MOS	importer	BP(S23_09170)<br>IM(S23_09160)<br>IM(S23_09180)<br>ABC(S23_09140)				BP(blr7868)<br>IM(blr7869)<br>IM(blr7867)<br>ABC(blr7871)				

17	HAA	importer	IM(S23_09550)<br>IM+ABC(S23_09540)<br>ABC(S23_09530)		BP		IM(blr7832)<br>IM+ABCblr7833)<br>ABC(blr7834)			
18	HAA	importer	BP(S23_09610)<br>IM(S23_09620)<br>IM(S23_09630)<br>ABC(S23_09640)<br>ABC(S23_09650)				BP(blr7827)<br>IM(blr7826)<br>IM(blr7825)<br>ABC(blr7824)<br>ABC(blr7823)			
19	OPN	importer	BP1(S23_09860)<br>BP2(S23_09870)<br>IM(S23_09880)<br>IM(S23_09890)<br>ABC(S23_09900)				BP1(blr1424)<br>BP2(-)<br>IM(blr1426)<br>IM(blr1427)<br>ABC(blr1428)			
20	HAA	importer	BP(S23_10120)<br>IM(S23_10130)<br>IM(S23_10140)<br>ABC(S23_10150)<br>ABC(S23_10160)				BP(blr1448)<br>IM(blr1449)<br>IM(blr1450)<br>ABC(blr1451)<br>ABC(blr1452)			
21	MOI	importer	BP(S23_10460)<br>IM(S23_10470)<br>IM(S23_10480)<br>ABC(S23_10490)			sulfate	BP(blr1482)<br>IM(blr1483)<br>IM(blr1484)<br>ABC(blr1485)			
22	HAA	importer	BP(S23_10540)<br>IM(S23_10560)<br>IM+ABC(S23_10550)<br>ABC(S23_10570)				-	-	-	*Rhodopseudomonas palustris* BisB18
23	DPL	exporter	IM+ABC(S23_10780)				IM+ABC(blr1508)			

24	PAO	importer	BP1(S23_11240)<br>BP2(S23_11270)<br>IM(S23_11250)<br>IM(S23_11260)		ABC		BP1(bll7206)<br>BP2(bll7203)<br>IM(bll7204)<br>IM(bll7205)			
25	MOI	importer	BP(S23_11330)<br>IM(S23_11350)<br>IM(S23_11360)<br>ABC(S23_11340)				BP(bll7197)<br>IM(bll7194)<br>IM(bll7195)<br>ABC(bll7196)			
26	MOI	importer	IM(S23_12230)<br>IM(S23_12240)<br>ABC(S23_12220)		BP		IM(bll7103)<br>IM(bll7104)<br>ABC(bll7105)			
27	OSP	importer	BP(S23_12270)<br>IM(S23_12250)<br>IM(S23_12260)		ABC		BP(bll7099)<br>IM(bll7101)<br>IM(bll7100)			
28	ISVH	importer	BP(S23_11430)<br>IM(S23_11420)<br>ABC(S23_11410)	*hmuT UV*		hemin	BP(blr7077)<br>IM(blr7078)<br>ABC(blr7079)			
29	OSP	importer	BP(S23_12570)<br>IM(S23_12550)<br>IM(S23_12560)		ABC		BP(blr7064)<br>IM(blr7066)<br>IM(blr7065)			
30	OTCN	importer	BP(S23_12920)<br>IM(S23_12950)<br>ABC(S23_12960)			aliphatic sulfonate	BP(bll7011)<br>IM(bll7009)<br>ABC(bll7008)			
31	HAA	importer	BP(S23_13730)<br>IM(S23_13740)<br>ABC(S23_13750)<br>ABC(S23_13760)				BP(bll6899)<br>IM(bll6898)<br>ABC(bll6897)<br>ABC(bll6896)			

32	MOI	importer	BP(S23_14470)<br>IM(S23_14480)<br>IM(S23_14490)<br>ABC(S23_14440)				BP(bll6825)<br>IM(bll6824)<br>IM(bll6823)<br>ABC(bll6828)			
33	PAO	importer	IM(S23_14580)<br>ABC(S23_14570)		BP		-	-	-	*Rhodopseudo monas palustris* BisB18
34	OSP	importer	BP(S23_14740)<br>IM(S23_14720)<br>IM(S23_14730)		ABC		-	-	-	IMs (*Oceanicola granulosus* HTCC2516)
35	HAA	importer	BP(S23_14800)		IM, ABC		BP(blr6804)			
36	MOI	importer	BP(S23_14820)<br>IM(S23_14830)<br>IM(S23_14840)<br>ABC(S23_14850)				-	BP(1905)<br>IM(1906)<br>IM(1907)<br>ABC(1908)	*Bradyrhizobium* sp. BTAi1	
37	OPN	importer	BP(S23_15960)<br>IM(S23_15970)<br>IM(S23_15980)		ABC		BP(bll6714)<br>IM(bll6713)<br>IM(bll6712)			
38	ART	cellular process	ABC+ABC(S23_16210)				ABC+ABC(bll6685)			
39	HAA	importer	BP(S23_16620)		IM, ABC		BP(blr6648)			
40	MOI	importer	BP(S23_16650)<br>IM(S23_16630)<br>IM(S23_16640)<br>ABC(S23_16660)				BP(blr6645)<br>IM(blr6647)<br>IM(blr6646)<br>ABC(blr6644)			
41	DPL	exporter	IM+ABC(S23_16980)				IM+ABC(bll6620)			

42	DRA	exporter	IM(S23_17420)<br>ABC(S23_17430)	*nodJ*			IM(blr6578)<br>ABC(blr6579)			
43	OTCN	importer	BP(S23_17880)<br>IM(S23_17860)<br>ABC(S23_17870)			sulfonate	-	-	-	-
44	HAA	importer	BP(S23_18040)<br>IM(S23_18050)<br>IM(S23_18060)<br>ABC(S23_18070)<br>ABC(S23_18080)				-	-	-	-
45	OTCN	importer	BP+IM(S23_18440<br>)<br>IM(S23_18470)<br>ABC(S23_18450)<br>ABC(S23_18460)				BP+IM(blr6490)<br>IM(bll6487)<br>ABC(blr6489)<br>ABC(bll6488)			
46	OTCN	importer	BP(S23_18530)<br>BP(S23_18550)<br>IM(S23_18580)<br>IM(S23_18590)<br>ABC(S23_18560)<br>ABC(S23_18570)				BP(bll6481)<br>BP(bll6479)<br>IM(bll6475)<br>IM(bll6476)<br>ABC(bll6477)<br>ABC(bll6478)			
47	HAA	importer	ABC(S23_18840)		BP, IM		ABC(blr6447)			
48	UVR	cellular process (excinuclease)	subunit A(S23_18850)	*uvrA*	subunit B, C		subunit A(blr8051)			
49	HAA	importer	BP(S23_18860)<br>IM(S23_18880)<br>IM(S23_18890)<br>ABC(S23_18870)				BP(blr6446)<br>IM(blr6443)<br>IM(blr6444)<br>ABC(blr6445)			

50	HAA	importer	BP(S23_19900)<br>BP(S23_19910)<br>IM(S23_19940)<br>IM+ABC(S23_19930)<br>ABC(S23_19920)				-	-	-	*Bordetella petrii* DSM 12804
51	OTCN	importer	BP(S23_20010)<br>IM(S23_20020)<br>IM(S23_20040)<br>ABC(S23_20030)				-	-	-	-
52	OTCN	importer	BP(S23_20430)<br>IM(S23_20420)<br>ABC(S23_20410)				BP(blr6246)<br>IM(blr6247)<br>ABC(blr6248)			
53	HAA	importer	BP(S23_20530)<br>IM(S23_20540)<br>IM(S23_20550)<br>ABC(S23_20560)<br>ABC(S23_20570)				BP(bll6236)<br>IM(bll6235)<br>IM(bll6234)<br>ABC(bll6233)<br>ABC(bll6232)			
54	MOI	importer	BP(S23_20630)<br>IM(S23_20640)		ABC	tungstate	BP(bll6227)<br>IM(bll6226)			
55	PAO	importer	BP(S23_20860)		IM, ABC		BP(blr6209)			
56	OTCN	importer	BP(S23_20950)<br>IM(S23_20940)<br>ABC(S23_20930)				BP(blr6199)<br>IM(blr6200)<br>ABC(blr6201)			
57	DRI	exporter	IM(S23_21030)<br>ABC(S23_21040)				IM(blr6191)<br>ABC(blr6190)			
58	MOS	importer	BP(S23_21360)<br>IM(S23_21370)<br>IM(S23_21380)<br>ABC(S23_21390)				BP(blr6158)<br>IM(blr6156)<br>IM(blr6157)<br>ABC(blr6155)			

59	MOS	importer	BP(S23_21460)<br>IM(S23_21440)<br>IM(S23_21450)<br>ABC(S23_21420)				BP(blr6148)<br>IM(blr6149)<br>IM(blr6150)<br>ABC(blr6152)			
60	OTCN	importer	BP(S23_21820)<br>IM(S23_21850)<br>IM(S23_21860)<br>ABC(S23_21840)				BP(bll6093)<br>IM(bll6089)<br>IM(bll6090)<br>ABC(bll6091)			
61	MKL	importer	BP(S23_22130)<br>IM(S23_22110)<br>ABC(S23_22120)				BP(bll6065)<br>IM(bll6064)<br>ABC(bll6063)			
62	HAA	importer	BP1(S23_22770)<br>BP2(S23_22780)<br>IM(S23_22790)<br>IM(S23_22800)<br>ABC(S23_22830)				BP1(blr5967)<br>BP2(blr5966)<br>IM(blr5964)<br>IM(blr5965)<br>ABC(blr5962)			
63	HAA	importer	BP(S23_22910)<br>IM(S23_22920)<br>IM(S23_22930)<br>ABC(S23_22940)<br>ABC(S23_22950)				BP(blr5953)<br>IM(blr5951)<br>IM(blr5952)<br>ABC(blr5949)<br>ABC(blr5950)			
64	DPL	exporter	IM+ABC(S23_23110)				IM+ABC(blr5932)			
65	MKL	importer	SS(S23_23650)<br>IM(S23_23630)<br>ABC(S23_23640)				SS(bll5892)<br>IM(bll5894)<br>ABC(bll5893)			
66	OTCN	importer	BP(S23_25160)<br>IM(S23_25170)<br>ABC(S23_25180)	*nrtABC*		nitrate	BP(bll5734)<br>IM(bll5733)<br>ABC(bll5732)			

67	HAA	importer	BP(S23_25540)<br>IM(S23_25520)<br>IM(S23_25530)<br>ABC(S23_25500)<br>ABC(S23_25510)				-	-	-	*Rhodopseudo monas palustris* BisB18
68	HAA	importer	BP(S23_25780)<br>IM(S23_25810)<br>IM(S23_25820)<br>ABC(S23_25790)<br>ABC(S23_25800)				BP(blr5675)<br>IM(blr5671)<br>IM(blr5672)<br>ABC(blr5673)<br>ABC(blr5674)			
69	DRI	exporter	ABC(S23_26050)<br>IM(S23_26060)			nitrate	ABC(bll5649)<br>IM(bll5648)			
70	OPN	importer	BP1(S23_26540)<br>BP2(S23_26550)<br>IM(S23_26500)<br>IM(S23_26510)<br>ABC(S23_26490)				BP1(blr5596)<br>BP1(blr5596)<br>IM(blr5598)<br>IM(blr5599)<br>ABC(blr5600)			
71	MOI	importer	BP(S23_26830)<br>IM(S23_26810)<br>IM(S23_26820)<br>ABC(S23_26840)				BP(blr5574)<br>IM(blr5575)<br>IM(blr5576)<br>ABC(blr5573)			
72	DPL	exporter	IM+ABC(S23_28730)				IM+ABC(bll5407)			
73	o228	exporter	IM(S23_29750)<br>ABC(S23_29760)				IM(blr5304)<br>ABC(blr5303)			
74	HAA	importer	BP(S23_29950)		IM, ABC		BP(blr5286)			
75	HAA	importer	BP(S23_29980)		IM, ABC		-	-	-	-
76	OPN	importer	BP(S23_31560)		IM,		BP(bll5056)			

					ABC					
77	OTCN	importer	BP(S23_32910)<br>IM(S23_32870)<br>IM(S23_32880)<br>ABC(S23_32890)				-	-	-	-
78	MOS	importer	BP(S23_32970)<br>IM(S23_32950)<br>ABC+ABC(S23_32960)				BP(blr4884)<br>IM(blr4886)<br>ABC+ABC(blr4885)			
79	MOI	importer	BP(S23_33750)		IM, ABC		BP(blr4797)			
80	UVR	cellular process	ABC+ABC subunitA (S23_34520)	*uvrA*			ABC+ABC(blr4702)			
81	OPN	importer	BP(S23_35210)		IM, ABC		BP(blr4590)			
82	OSP	importer	BP(S23_35580)<br>IM(S23_35560)<br>IM(S23_35570)<br>ABC(S23_35550)				BP(blr4553)<br>IM(blr4554)<br>IM(blr4556)<br>ABC(blr4557)			
83	OPN	importer	BP(S23_35670)<br>IM(S23_35700)<br>IM(S23_35710)<br>ABC(S23_35680)<br>ABC(S23_35690)				BP(bll4544)<br>IM(bll4540)<br>IM(bll4541)<br>ABC(bll4542)<br>ABC(bll4543)			
84	FAE	exporter	IM+ABC(S23_36480)				IM+ABC(blr4660)			
85	DLM	importer	BP(S23_36720)<br>IM(S23_36730)<br>ABC(S23_36740)			methionine	BP(-)<br>IM(blr4502)<br>ABC(blr4501)	BP(3285)<br>IM(3282)<br>ABC(3281)	*Bradyrhizobium* sp. BTAi1	

86	ISVH	importer	BP(S23_36830)<br>IM(S23_36840)<br>ABC(S23_36850)			iron siderophore	-	-	-	*Rhodopseudo monas palustris* TIE-1
87	PAO	importer	BP(S23_37210)<br>ABC(S23_37200)		IM		BP(blr4464)<br>ABC(blr4463)			
88	PAO	importer	BP(S23_37370)<br>IM(S23_37350)<br>IM(S23_37360)<br>ABC(S23_37340)	*aapP* (S23_3 7340)		amino acid	BP(blr4446)<br>IM(blr4447)<br>IM(blr4448)<br>ABC(blr4449)			
89	CLS	exporter	ABC(S23_39690)		IM		ABC(bll4315)			
90	HAA	importer	BP(S23_39720)		IM, ABC		BP(blr4312)			
91	OTCN	importer	IM(S23_40560)		BP, ABC		IM(bll4208)			
92	OTCN	importer	ABC(S23_40600)		BP, IM		ABC(bll4204)			
93	ART	cellular process	ABC+ABC(S23_4 0760)				ABC+ABC)blr4184			
94	HAA	importer	BP(S23_40810)		IM, ABC		-	-	-	*Methylobacter ium nodulans* ORS 2060
95	MOI	importer	BP(S23_40850)		IM, ABC		BP(blr4171)			
96	OSP	importer	BP(S23_41250)<br>IM(S23_41230)<br>IM(S23_41240)		ABC		BP(bll4139)<br>IM(bll4140)<br>IM(bll4138)			
97	PAO	importer	BP(S23_41980)<br>IM(S23_41950)<br>IM(S23_41960)<br>ABC(S23_41970)				-	-	-	*Methylobacter ium* sp. 4-46

98	HAA	importer	BP(S23_42210)<br>IM(S23_42190)<br>IM(S23_42200)<br>ABC(S23_42220)<br>ABC(S23_42230)				BP(blr4039)<br>IM(blr4040)<br>IM(blr4041)<br>ABC(blr4037)<br>ABC(blr4038)			
99	OSP	importer	BP(S23_43160)<br>IM1(S23_43140)<br>IM2(S23_43150)<br>ABC(S23_43170)				ABC(blr3917)	-	-	BP and IM1 (*Polaromonas* sp. JS666)
100	HAA	importer	BP(S23_43430)<br>IM(S23_43410)<br>IM(S23_43420)<br>ABC(S23_43390)<br>ABC(S23_43400)				-	-	-	-
101	OTCN	importer	BP(S23_43910)<br>IM(S23_43920)<br>ABC(S23_43930)				-	-	-	*Methylobacter ium* sp. 4-46
102	HAA	importer	BP(S23_44170)<br>IM(S23_44160)<br>ABC(S23_44140)<br>ABC(S23_44150)				BP(blr3825)<br>IM(blr3826)<br>ABC(blr3827)<br>ABC(blr3828)			
103	PHN	importer	ABC(S23_44270)		BP, IM		-	-	-	-
104	DPL	exporter	IM+ABC(S23_445 30)				IM+ABC(blr3795)			
105	HAA	importer	IM(S23_45180)<br>IM(S23_45190)<br>ABC(S23_45200)<br>ABC(S23_45210)		BP		IM(bll3384)<br>IM(bll3385)<br>ABC(bll3382)<br>ABC(bll3383)			
106	HAA	importer	IM+ABC(S23_453 70)		BP		-	-	-	*Bordetella avium* 197N

			ABC(S23_45360)							
107	OSP	importer	BP(S23_45460)<br>IM(S23_45440)<br>IM(S23_45450)<br>ABC(S23_45420)				BP(bll6356)<br>IM(bll6354)<br>IM(bll6355)<br>ABC(bll6352)			
108	HAA	importer	BP1(S23_45610)<br>BP2(S23_45620)<br>IM(S23_45630)<br>IM(S23_45640)<br>ABC(S23_45650)<br>ABC(S23_45660)				-	BP1(4448)<br>BP2(4449)<br>IM(4450)<br>IM(4451)<br>ABC(4452)<br>ABC(4453)	*Bradyrhizobium* sp. BTAi1	
109	OPN	importer	BP(S23_45830)<br>IM(S23_45810)<br>IM(S23_45820)<br>ABC+ABC(S23_4<br>5800)				-	-	-	-
110	MOI	importer	BP(S23_45870)<br>IM(S23_45880)<br>ABC(S23_45890)	*modA BC*		molybdenum	BP(blr6951)<br>IM(blr6952)<br>ABC(blr6953)			
111	DLM	importer	BP(S23_46600)		IM,<br>ABC	D-methionine	-	BP(3291)	*Bradyrhizobium* sp. BTAi1	
112	HAA	importer	BP(S23_46740)<br>IM(S23_46720)<br>IM(S23_46730)<br>ABC(S23_46700)<br>ABC(S23_46710)				-	BP(3206)<br>IM(3203)<br>IM(3204)<br>ABC(3205)<br>ABC(3206)	*Bradyrhizobium* sp. BTAi1	

113	MOS	importer	BP(S23_46870)<br>IM(S23_46850)<br>IM(S23_46860)<br>ABC+ABC(S23_4<br>6840)				BP(bll2674)<br>IM(bll2675)<br>IM(bll2676)<br>ABC+ABC(bll2677)			
114	OPN	importer	BP(S23_46930)<br>IM(S23_46910)<br>IM(S23_46920)<br>ABC(S23_46890)<br>ABC(S23_46900)				BP(blr3341)<br>IM(blr3342)<br>IM(blr3343)<br>ABC(blr3344)<br>ABC(blr3345)			
115	OPN	importer	BP(S23_46970)<br>IM(S23_46950)<br>IM(S23_46960)<br>ABC+ABC(S23_4<br>6940)				BP(blr3337)<br>IM(blr3338)<br>IM(blr3339)<br>ABC+ABC(blr3340)			
116	OTCN	importer	BP(S23_46140)<br>IM(S23_46130)<br>ABC(S23_46120)				BP(blr3291)<br>IM(blr3292)<br>ABC(blr3293)			
117	MOI	importer	BP(S23_46190)<br>IM(S23_46200)<br>IM(S23_46210)<br>ABC(S23_46180)				BP(bll3284)<br>IM(bll3285)<br>IM(bll3286)<br>ABC(bll3287)			
118	OPN	importer	BP(S23_47650)<br>IM(S23_47680)<br>IM(S23_47690)<br>ABC(S23_47660)<br>ABC(S23_47670)				BP(blr3234)<br>IM(blr3230)<br>IM(blr3231)<br>ABC(blr3232)<br>ABC(blr3233)			
119	OSP	importer	BP(S23_47770)<br>IM(S23_47750)<br>IM(S23_47760)<br>ABC(S23_47740)				BP(blr3221)<br>IM(blr3222)<br>IM(blr3223)<br>ABC(blr3224)			

120	MOS	importer	BP(S23_47890)<br>IM(S23_47870)<br>ABC(S23_47880)	*chvE* (S23_47890)		multiple sugars	BP(blr3208)<br>IM(blr3210)<br>ABC(blr3209)			
121	MOS	importer	BP(S23_47970)<br>IM(S23_47940)<br>IM(S23_47950)<br>ABC+ABC(S23_47960)				BP(blr3200)<br>IM(blr3202)<br>IM(blr3203)<br>ABC+ABC(blr3201)			
122	OTCN	importer	BP(S23_48050)<br>IM(S23_48060)<br>ABC(S23_48070)				BP(blr3192)<br>IM(blr3191)<br>ABC(blr3190)			
123	HAA	importer	BP(S23_48140)<br>IM(S23_48120)<br>IM(S23_48130)<br>ABC(S23_48100)<br>ABC(S23_48110)				BP(blr3183)<br>IM(blr3184)<br>IM(blr3185)<br>ABC(blr3186)<br>ABC(blr3187)			
124	OSP	importer	BP(S23_48270)<br>IM(S23_48250)<br>IM(S23_48260)<br>ABC(S23_48240)				-	-	-	*Rhodopseudomonas palustris*
125	HAA	importer	BP1(S23_50890)<br>BP2(S23_50930)<br>IM(S23_50910)<br>IM(S23_50920)<br>ABC(S23_50900)				BP2(blr2922)<br>BP1(blr2926)<br>IM(blr2923)<br>IM(blr2924)<br>ABC(blr2925)			
126	PAO	importer	IM(S23_51020)<br>IM(S23_51030)		BP, ABC		IM(blr2913)<br>IM(blr2912)			

127	OPN	importer	BP(S23_51390)<br>IM(S23_51400)<br>IM(S23_51410)<br>ABC(S23_51420)<br>ABC(S23_51430)				BP(bll2877)<br>IM(bll2875)<br>IM(bll2876)<br>ABC(bll2873)<br>ABC(bll2874)			
128	OPN	importer	BP(S23_51470)<br>IM(S23_51480)<br>IM(S23_51490)<br>ABC(S23_51450)<br>ABC(S23_51460)				BP(bll2868)<br>IM(bll2866)<br>IM(bll2867)<br>ABC(bll2869)<br>ABC(bll2870)			
129	OTCN	importer	BP(S23_51620)<br>IM(S23_51630)<br>ABC(S23_51610)				BP(blr2837)<br>IM(blr2836)<br>ABC(blr2838)			
130	OTCN	importer	BP(S23_51940)<br>IM(S23_51930)<br>ABC(S23_51920)	*nrtAB C*		nitrate	BP(blr2803)<br>IM(blr2804)<br>ABC(blr2805)			
131	OPN	importer	BP(S23_52190)<br>IM(S23_52170)<br>IM(S23_52180)<br>ABC(S23_52150)<br>ABC(S23_52160)				-	-	-	*Geobacter uraniireducens* Rf4
132	DPL	exporter	IM+ABC(S23_524 70)				IM+ABC(blr2753)			
133	DPL	exporter	IM+ABC(S23_530 70)<br>IM+ABC(S23_530 80)				-	-	-	*Methylobacterium* sp. 4-46<br>*Methylobacterium nodulans* ORS 2060

134	OSP	importer	BP(S23_55680)<br>IM(S23_55700)<br>IM(S23_55710)<br>ABC(S23_55720)<br>ABC(S23_55730)				BP(blr2442)<br>IM(blr2439)<br>IM(blr2440)<br>ABC(blr2437)<br>ABC(blr2438)			
135	HAA	importer	BP(S23_56290)		IM,<br>ABC		BP(blr7829)			
136	HAA	importer	BP(S23_57120)		IM,<br>ABC		-	-	-	*Rhodopseudo monas palustris* BisB5
137	HAA	importer	BP(S23_57300)		IM,<br>ABC		BP(bll2304)			
138	HAA	importer	BP(S23_57690)<br>IM(S23_57670)<br>IM(S23_57680)<br>ABC(S23_57650)<br>ABC(S23_57660)				-	-	-	*Rhodopseudo monas palustris*
139	PAO	importer	BP(S23_57970)<br>IM(S23_57960)<br>ABC(S23_57950)				BP(blr2224)<br>IM(blr2225)<br>ABC(blr2226)			
140	MOI	importer	BP(S23_59210)<br>IM(S23_59220)<br>IM(S23_59230)<br>ABC(S23_59200)				-	BP(3187)<br>IM(3185)<br>IM(3186)<br>ABC(3188)	*Bradyrhizobium* sp. BTAi1	
141		excinuclease	component B(S23_60490)	*uvrB*			component B(blr7431)			
142		excinuclease	component C(S23_61010)	*uvrC*			component C(blr7470)			
143	FAE	exporter	IM+ABC(S23_616 90)				IM+ABC(blr7537)			

144	PAO	importer	BP(S23_62180)<br>IM(S23_62160)<br>IM(S23_62170)<br>ABC(S23_62150)	*gltIJK L*		glutamate/ aspartate	BP(bll7600)<br>IM(bll7598)<br>IM(bll7599)<br>ABC(bll7597)			
145	OPN	importer	BP(S23_62310)		IM, ABC		BP(blr7616)			
146	DPL	exporter	IM+ABC(S23_625 70)				IM+ABC(bll6293)			
147	MET	importer	BP(S23_63330)<br>IM(S23_63340)<br>ABC(S23_63350)				BP(bll7769)<br>IM(bll7770)<br>ABC(bll7771)			
148	OPN	importer	BP(S23_64360)<br>IM(S23_64340)<br>IM(S23_64350)<br>ABC(S23_64320)<br>ABC(S23_64330)				BP(blr1353)<br>IM(blr1354)<br>IM(blr1355)<br>ABC(blr1356)<br>ABC(blr1357)			
149	OTCN	importer	BP(S23_65210)<br>IM(S23_65220)<br>ABC(S23_65230)				-	-	-	*Achromobacte r piechaudii* ATCC 43553
150	OTCN	importer	BP(S23_65630)		IM, ABC		BP(blr1233)			
151	NO	unknown	ABC(S23_65700)<br>ABC(S23_65710)	*phnKL*		phosphonate	ABC(blr1226)<br>ABC(blr1225)			
152	PHN	importer	BP(S23_65790)<br>IM(S23_65770)<br>IM(S23_65780)<br>ABC(S23_65800)	*phoCD ET*		phosphonate	BP(bll7946)<br>IM(bll7944)<br>IM(bll7945)<br>ABC(bll7947)			
153	OTCN	importer	BP(S23_66030)<br>IM(S23_66010)<br>ABC(S23_66020)				-	BP(5375)<br>IM(5373)<br>ABC(5374)	*Bradyrhizobium* sp. ORS278	

154	PAO	importer	BP(S23_66470)<br>IM(S23_66480)<br>ABC(S23_66490)				BP(blr8117)<br>IM(blr8118)<br>ABC(blr8119)			
155	HAA	importer	BP(S23_66760)		IM,<br>ABC		BP(blr1147)			
156	MOS	importer	BP(S23_66960)<br>IM(S23_66970)<br>ABC(S23_66980)				BP(blr1123)<br>IM(blr1122)<br>ABC(blr1121)			
157	MOI	importer	BP(S23_67280)<br>IM(S23_67260)<br>IM(S23_67270)<br>ABC(S23_67250)	*pstAB CS*		phosphate	BP(blr1091)<br>IM(blr1092)<br>IM(blr1093)<br>ABC(blr1094)			
158	HAA	importer	BP(S23_67550)<br>IM(S23_67480)<br>IM(S23_67490)<br>ABC(S23_67460)<br>ABC(S23_67470)				BP(blr1052)<br>IM(blr1064)<br>IM(blr1065)<br>ABC(blr1066)<br>ABC(blr1067)			
159	OPN	importer	BP(S23_67710)<br>IM(S23_67680)<br>IM(S23_67690)<br>ABC+ABC(S23_67670)				BP(blr1036)<br>IM(blr1037)<br>IM(blr1038)<br>ABC+ABC(blr1039)			
160	o228	exporter	IM+IM(S23_68050)<br>ABC(S23_68060)				IM+IM(bll1001)<br>ABC(bll1002)			
161	OPN	importer	BP(S23_68140)<br>IM(S23_68150)<br>IM(S23_68160)<br>ABC(S23_68170)<br>ABC(S23_68180)				BP(bll0993)<br>IM(bll0992)<br>IM(bll0991)<br>ABC(bll0990)<br>ABC(bll0989)			

162	HAA	importer	BP(S23_68450)<br>IM(S23_68430)<br>IM(S23_68440)<br>ABC(S23_68410)<br>ABC(S23_68420)				BP(blr0967)<br>IM(blr0968)<br>IM(blr0969)<br>ABC(blr0970)<br>ABC(blr0971)			
163	HAA	importer	BP(S23_69170)<br>IM(S23_69180)<br>IM(S23_69190)<br>ABC(S23_69200)<br>ABC(S23_69210)				BP(bll0887)<br>IM(bll0885)<br>IM(bll0886)<br>ABC(bll0883)<br>ABC(bll0884)			

BP, solute-binding protein component; IM, integral membrane protein component; ABC, ATP-binding cassette component; SS, protein with a signal sequence.

*To classify the systems, at least two components of each ABC system were used in a BLAST search of the ABCISSE database (http://www.pasteur.fr/recherche/unites/pmtg/abc/index.html), unless a system was composed of only one component.

**Orthologues were defined as genes whose encoded proteins show more than 70%, 60%, and 50% identities in amino acid sequence when compared with *B. japonicum* USDA110, other *Rhizobiaceae*, and other bacteria, respectively. Orthologues classified as “other *Rhizobiaceae*” were not found in *B. japonicum* USDA110, while those classified as “other bacteria” were not found in either *B. japonicum* USDA110 or *Rhizobiaceae*.

Table S7. Compositions of ABC systems in the genomes of *Bradyrhizobium* sp. S23321 and related species.

Family*	*Bradyrhizobium* sp. S23321	*Bradyrhizobium japonicum* USDA110**	*Mesorhizobium loti* MAFF303099**	*Pseudomonas putida* KT2440**	*Streptomyces coelicolor* A3(2)**	Family definition*	Functions*
ART	2	5	5	4	8	Antibiotic resistance and translation regulation	Cellular processes
CBY	0	0	0	0	3	Cobalt uptake, unknown	Import
CCM	0	1	1	1	0	Cytochrome C biogenesis	Export
CDI	0	1	1	1	1	Cell division	Unknown
CLS	1	2	1	1	2	Capsular polysaccharide, lipopolysaccharide and teichoic acids	Export
DLM	2	1	1	2	1	D- L-Methionine and derivatives	Import
DPL	9	14	15	13	18	Drugs, Peptides, Lipids	Export
DRA	1	2	2	1	16	Drug and Antibiotic resistance	Unknown
DRI	4	6	2	2	14	Drug resistance, bacteriocin and lantibiotic immunity	Unknown
EPD	0	0	0	0	1	Eye pigment precursors and drugs	Export
FAE	2	3	2	1	0	Very long chain fatty acids	Export
HAA	39	34	12	6	3	Hydrophobic amino acids and amides	Import
ISB	0	1	1	0	0	Iron-sulfur centre biogenesis	Cellular processes
ISVH	2	12	2	28	6	Iron-Siderophores, Vitamin B-12 and Hemin	Import
MET	1	1	1	2	2	Metals	Import
MKL	2	2	1	2	1		Import
MOI	15	16	28	13	7	Mineral and Organic	Import
MOS	9	11	37	2	8	Monosaccharides	Import
NO	1	2	7	0	2	Unclassified systems	Unknown
OPN	19	22	30	6	10	Oligopeptides and Nickel	Import
OSP	11	15	26	2	37	Oligosaccharides and polyols	Import

OTCN	23	22	16	10	7	Osmoprotectants Taurine Cyanate and Nitrate	Import
PAO	10	17	14	10	4	Polar amino acid and opines	Import
PHN	2	1	2	1	0	Phosphonates and phosphites	Import
Total	160	201	213	116	171		
UVR	2	2	1	2	4	DNA repair and drug resistance	Cellular processes
YHBG	0	1	1	1	0		
YLU	0	0		0	2		Unknown
o228	3	7	4	5	14		Unknown

*Names and definitions of the families are cited from the ABCISSE database.

**Data were obtained from the ABCISSE database (http://www.pasteur.fr/recherche/unites/pmtg/abc/index.html).

Table S8. Genes for major carbon metabolism pathways in the genome of Bradyrhizobium sp. S23321.

Pathways	Locus tag	KO number	Gene symbol	Function
Glycolysis/gluconeogenesis	S23_01500	K01834	*gpm*	phosphoglycerate mutase [EC:5.4.2.1]
Glycolysis/gluconeogenesis	S23_02590	K01895	*acs*	acetyl-CoA synthetase [EC:6.2.1.1]
Glycolysis/gluconeogenesis	S23_03930	K00382	*lpd, pdhD*	dihydrolipoamide dehydrogenase [EC:1.8.1.4]
Glycolysis/gluconeogenesis	S23_06870	K00128		aldehyde dehydrogenase (NAD+) [EC:1.2.1.3]
Glycolysis/gluconeogenesis	S23_07050	K01895		coenzyme A ligase [EC:6.2.1.1]
Glycolysis/gluconeogenesis	S23_07640	K01610	*pckA*	phosphoenolpyruvate carboxykinase (ATP) [EC:4.1.1.49]
Glycolysis/gluconeogenesis	S23_08870	K00121	*adhC*	S-(hydroxymethyl)glutathione dehydrogenase / alcohol dehydrogenase [EC:1.1.1.284 1.1.1.1]
Glycolysis/gluconeogenesis	S23_10960	K01623	*fbaB*	fructose-bisphosphate aldolase, class I [EC:4.1.2.13]
Glycolysis/gluconeogenesis	S23_10970	K01624	*fbaA*	fructose-bisphosphate aldolase, class II [EC:4.1.2.13]
Glycolysis/gluconeogenesis	S23_10980	K00927	*pgk*	phosphoglycerate kinase [EC:2.7.2.3]
Glycolysis/gluconeogenesis	S23_10990	K00134	*gapdh*	glyceraldehyde 3-phosphate dehydrogenase [EC:1.2.1.12]
Glycolysis/gluconeogenesis	S23_11900	K00873	*pyk*	pyruvate kinase [EC:2.7.1.40]
Glycolysis/gluconeogenesis	S23_15550	K13810	*tal-pgi*	transaldolase / glucose-6-phosphate isomerase [EC:2.2.1.2 5.3.1.9]
Glycolysis/gluconeogenesis	S23_20800	K00121	*adhC*	S-(hydroxymethyl)glutathione dehydrogenase / alcohol dehydrogenase [EC:1.1.1.284 1.1.1.1]
Glycolysis/gluconeogenesis	S23_20820	K00114		methanol dehydrogenase large subunit-like protein [EC:1.1.99.8]
Glycolysis/gluconeogenesis	S23_20880	K00114		quinoprotein ethanol dehydrogenase precursor [EC:1.1.99.8]
Glycolysis/gluconeogenesis	S23_22000	K01895	*acs*	acetyl-CoA synthetase [EC:6.2.1.1]
Glycolysis/gluconeogenesis	S23_22060	K00001	*adh*	alcohol dehydrogenase [EC:1.1.1.1]
Glycolysis/gluconeogenesis	S23_23760	K00128		aldehyde dehydrogenase (NAD+) [EC:1.2.1.3]
Glycolysis/gluconeogenesis	S23_25980	K00001	*adh*	alcohol dehydrogenase [EC:1.1.1.1]
Glycolysis/gluconeogenesis	S23_32850	K00001	*adh*	Iron-containing alcohol dehydrogenase [EC:1.1.1.1]
Glycolysis/gluconeogenesis	S23_33100	K00001	*adh*	alcohol dehydrogenase [EC:1.1.1.1]
Glycolysis/gluconeogenesis	S23_33640	K01803	*tpiA*	triosephosphate isomerase (TIM) [EC:5.3.1.1]
Glycolysis/gluconeogenesis	S23_33770	K01689	*eno*	enolase [EC:4.2.1.11]
Glycolysis/gluconeogenesis	S23_33840	K00161	*pdhA*	pyruvate dehydrogenase E1 component subunit alpha [EC:1.2.4.1]
Glycolysis/gluconeogenesis	S23_33850	K00162	*pdhB*	pyruvate dehydrogenase E1 component subunit beta [EC:1.2.4.1]
Glycolysis/gluconeogenesis	S23_33880	K00627	*aceF, pdhC*	pyruvate dehydrogenase E2 component (dihydrolipoamide acetyltransferase) [EC:2.3.1.12]
Glycolysis/gluconeogenesis	S23_33900	K00382	*lpd, pdhD*	dihydrolipoamide dehydrogenase [EC:1.8.1.4]
Glycolysis/gluconeogenesis	S23_35620	K00845	*glk*	glucokinase [EC:2.7.1.2]
Glycolysis/gluconeogenesis	S23_36470	K00850	*pfk*	6-phosphofructokinase [EC:2.7.1.11]
Glycolysis/gluconeogenesis	S23_39180	K02446	*glpX*	fructose-1,6-bisphosphatase II [EC:3.1.3.11]
Glycolysis/gluconeogenesis	S23_41610	K00001	*adh*	putative NAD-dependent alcohol dehydrogenase [EC:1.1.1.1]
Glycolysis/gluconeogenesis	S23_42810	K01895	*acs*	acetyl-CoA synthetase [EC:6.2.1.1]
Glycolysis/gluconeogenesis	S23_44870	K00001	*adh*	Alcohol dehydrogenase GroES domain protein [EC:1.1.1.1]
Glycolysis/gluconeogenesis	S23_47910	K01785	*galM*	aldose 1-epimerase [EC:5.1.3.3]
Glycolysis/gluconeogenesis	S23_51700	K01834	*gpm*	phosphoglycerate mutase [EC:5.4.2.1]
Glycolysis/gluconeogenesis	S23_51810	K00128		aldehyde dehydrogenase (NAD+) [EC:1.2.1.3]
Glycolysis/gluconeogenesis	S23_51820	K00163	*aceE*	pyruvate dehydrogenase E1 component [EC:1.2.4.1]
Glycolysis/gluconeogenesis	S23_52140	K00001	*adh*	NAD-dependent alcohol dehydrogenase [EC:1.1.1.1]
Glycolysis/gluconeogenesis	S23_53980	K01624	*fbaA*	fructose-bisphosphate aldolase, class II [EC:4.1.2.13]
Glycolysis/gluconeogenesis	S23_54010	K03841	*fbp*	fructose-1,6-bisphosphatase I [EC:3.1.3.11]
Glycolysis/gluconeogenesis	S23_56190	K01835	*pgm*	phosphoglucomutase [EC:5.4.2.2]
Glycolysis/gluconeogenesis	S23_59680	K01895	*acs*	acetyl-CoA synthetase [EC:6.2.1.1]
Glycolysis/gluconeogenesis	S23_61840	K00001	*adh*	alcohol dehydrogenase [EC:1.1.1.1]
Glycolysis/gluconeogenesis	S23_62250	K00128		aldehyde dehydrogenase (NAD+) [EC:1.2.1.3]
Glycolysis/gluconeogenesis	S23_66610	K01835	*pgm*	phosphoglucomutase [EC:5.4.2.2]
Glycolysis/gluconeogenesis	S23_67390	K01895	*acs*	acetyl-CoA synthetase [EC:6.2.1.1]
Glycolysis/gluconeogenesis	S23_68350	K01895	*acs*	acetyl-CoA synthetase [EC:6.2.1.1]
Citrate cycle	S23_03230	K00240	*sdhB*	succinate dehydrogenase iron-sulfur protein [EC:1.3.99.1]
Citrate cycle	S23_03240	K00239	*sdhA*	succinate dehydrogenase flavoprotein subunit [EC:1.3.99.1]
Citrate cycle	S23_03250	K00242	*sdhD*	succinate dehydrogenase hydrophobic membrane anchor protein [EC:1.3.99.1]
Citrate cycle	S23_03260	K00241	*sdhC*	succinate dehydrogenase cytochrome b-556 subunit [EC:1.3.99.1]
Citrate cycle	S23_03410	K01644	*citE*	citrate lyase subunit beta / citryl-CoA lyase [EC:4.1.3.6 4.1.3.34]
Citrate cycle	S23_03750	K01681	*acnA*	aconitate hydratase 1 [EC:4.2.1.3]
Citrate cycle	S23_03860	K00024	*mdh*	malate dehydrogenase [EC:1.1.1.37]
Citrate cycle	S23_03870	K01903	*sucC*	succinyl-CoA synthetase beta subunit [EC:6.2.1.5]
Citrate cycle	S23_03890	K01902	*sucD*	succinyl-CoA synthetase alpha subunit [EC:6.2.1.5]
Citrate cycle	S23_03900	K00164	*sucA*	2-oxoglutarate dehydrogenase E1 component [EC:1.2.4.2]
Citrate cycle	S23_03910	K00658	*sucB*	2-oxoglutarate dehydrogenase E2 component (dihydrolipoamide succinyltransferase) [EC:2.3.1.61]
Citrate cycle	S23_03930	K00382	*lpd, pdhD*	dihydrolipoamide dehydrogenase [EC:1.8.1.4]
Citrate cycle	S23_06190	K01647	*gltA*	citrate synthase [EC:2.3.3.1]
Citrate cycle	S23_06200	K01647	*gltA*	citrate synthase [EC:2.3.3.1]
Citrate cycle	S23_07640	K01610	*pckA*	phosphoenolpyruvate carboxykinase (ATP) [EC:4.1.1.49]
Citrate cycle	S23_14960	K01644	*citE*	citrate lyase subunit beta / citryl-CoA lyase [EC:4.1.3.6 4.1.3.34]
Citrate cycle	S23_15690	K00175	*korB*	2-oxoglutarate ferredoxin oxidoreductase subunit beta [EC:1.2.7.3]
Citrate cycle	S23_15700	K00174	*korA*	2-oxoglutarate ferredoxin oxidoreductase subunit alpha [EC:1.2.7.3]
Citrate cycle	S23_18170	K01679	*fumC*	fumarate hydratase, class II [EC:4.2.1.2]
Citrate cycle	S23_24400	K01676		fumarate hydratase, class I [EC:4.2.1.2]
Citrate cycle	S23_24990	K00031	*icd*	isocitrate dehydrogenase [EC:1.1.1.42]
Citrate cycle	S23_33420	K01647		type II citrate synthase
Citrate cycle	S23_33840	K00161	*pdhA*	pyruvate dehydrogenase E1 component subunit alpha [EC:1.2.4.1]
Citrate cycle	S23_33850	K00162	*pdhB*	pyruvate dehydrogenase E1 component subunit beta [EC:1.2.4.1]
Citrate cycle	S23_33880	K00627	*aceF, pdhC*	pyruvate dehydrogenase E2 component (dihydrolipoamide acetyltransferase) [EC:2.3.1.12]
Citrate cycle	S23_33900	K00382	*lpd, pdhD*	dihydrolipoamide dehydrogenase [EC:1.8.1.4]
Citrate cycle	S23_51820	K00163	*aceE*	pyruvate dehydrogenase E1 component [EC:1.2.4.1]
Pentose phosphate pathway	S23_04680	K07404	*pgl*	6-phosphogluconolactonase [EC:3.1.1.31]
Pentose phosphate pathway	S23_10960	K01623	*fbaB*	fructose-bisphosphate aldolase, class I [EC:4.1.2.13]
Pentose phosphate pathway	S23_10970	K01624	*fbaA*	fructose-1,6-bisphosphate aldolase, class II [EC:4.1.2.13]
Pentose phosphate pathway	S23_11000	K00615		transketolase [EC:2.2.1.1]

Pentose phosphate pathway	S23_11440	K00034	*gdh*	glucose 1-dehydrogenase [EC:1.1.1.47]
Pentose phosphate pathway	S23_12580	K00090		gluconate 2-dehydrogenase [EC:1.1.1.215]
Pentose phosphate pathway	S23_14520	K00115		glucose dehydrogenase (acceptor) [EC:1.1.99.10]
Pentose phosphate pathway	S23_15510	K00851	*gntK, idnK*	gluconokinase [EC:2.7.1.12]
Pentose phosphate pathway	S23_15520	K01057	*pgl, devB*	6-phosphogluconolactonase [EC:3.1.1.31]
Pentose phosphate pathway	S23_15530	K00036	*G6PD, zwf*	glucose-6-phosphate 1-dehydrogenase [EC:1.1.1.49]
Pentose phosphate pathway	S23_15540	K00033	*gnd*	6-phosphogluconate dehydrogenase [EC:1.1.1.44]
Pentose phosphate pathway	S23_15550	K13810	*tal-pgi*	transaldolase / glucose-6-phosphate isomerase [EC:2.2.1.2 5.3.1.9]
Pentose phosphate pathway	S23_18910	K01053		gluconolactonase [EC:3.1.1.17]
Pentose phosphate pathway	S23_22510	K00615	*tktA*	transketolase subunit A
Pentose phosphate pathway	S23_22520	K00615		transketolase domain-containing protein
Pentose phosphate pathway	S23_24770	K00117	*gcd*	quinoprotein glucose dehydrogenase [EC:1.1.5.2]
Pentose phosphate pathway	S23_25720	K01783	*rpe*	ribulose-phosphate 3-epimerase [EC:5.1.3.1]
Pentose phosphate pathway	S23_36470	K00850	*pfk*	6-phosphofructokinase [EC:2.7.1.11]
Pentose phosphate pathway	S23_39180	K02446	*glpX*	fructose-1,6-bisphosphatase II [EC:3.1.3.11]
Pentose phosphate pathway	S23_41990	K01808	*rpiB*	ribose 5-phosphate isomerase B [EC:5.3.1.6]
Pentose phosphate pathway	S23_43110	K01625	*eda*	2-dehydro-3-deoxyphosphogluconate aldolase / 4-hydroxy-2-oxoglutarate aldolase [EC:4.1.2.14 4.1.3.16]
Pentose phosphate pathway	S23_43120	K00874	*kdgK*	2-dehydro-3-deoxygluconokinase [EC:2.7.1.45]
Pentose phosphate pathway	S23_44940	K01807	*rpiA*	ribose 5-phosphate isomerase A [EC:5.3.1.6]
Pentose phosphate pathway	S23_48880	K00948		phosphoribosylpyrophosphate synthetase
Pentose phosphate pathway	S23_50560	K01053		gluconolactonase [EC:3.1.1.17]
Pentose phosphate pathway	S23_51670	K00852	*rbsK*	ribokinase [EC:2.7.1.15]
Pentose phosphate pathway	S23_51800	K01053		gluconolactonase [EC:3.1.1.17]
Pentose phosphate pathway	S23_53940	K01783	*rpe*	ribulose-phosphate 3-epimerase [EC:5.1.3.1]
Pentose phosphate pathway	S23_53980	K01624	*fbaA*	fructose-1,6-bisphosphate aldolase, class II [EC:4.1.2.13]
Pentose phosphate pathway	S23_53990	K00615		transketolase [EC:2.2.1.1]
Pentose phosphate pathway	S23_54010	K03841	*fbp*	fructose-1,6-bisphosphatase I [EC:3.1.3.11]
Pentose phosphate pathway	S23_56190	K01835	*pgm*	phosphoglucomutase [EC:5.4.2.2]
Pentose phosphate pathway	S23_60720	K00948	*prsA*	ribose-phosphate pyrophosphokinase [EC:2.7.6.1]
Pentose phosphate pathway	S23_65680	K05774	*phnN*	ribose 1,5-bisphosphokinase [EC:2.7.4.23]
Pentose phosphate pathway	S23_66610	K01835		phosphoglucomutase phosphomannomutase protein [EC:5.4.2.2]
Glyoxylate cycle	S23_03750	K01681	*acnA*	aconitate hydratase [EC:4.2.1.3]
Glyoxylate cycle	S23_03860	K00024	*mdh*	malate dehydrogenase [EC:1.1.1.37]
Glyoxylate cycle	S23_06190	K01647	*gltA*	citrate synthase [EC:2.3.3.1]
Glyoxylate cycle	S23_06200	K01647	*gltA*	citrate synthase [EC:2.3.3.1]
Glyoxylate cycle	S23_10380	K01638	*aceB*	malate synthase [EC:2.3.3.9]
Glyoxylate cycle	S23_33420	K01647		type II citrate synthase
Glyoxylate cycle	S23_55560	K01637	*aceA*	isocitrate lyase [EC:4.1.3.1]
Fatty acid biosynthesis	S23_00580	K00208	*fabI*	enoyl-[acyl-carrier protein] reductase I [EC:1.3.1.9]
Fatty acid biosynthesis	S23_00590	K00647	*fabB*	3-oxoacyl-[acyl-carrier-protein] synthase I [EC:2.3.1.41]
Fatty acid biosynthesis	S23_00600	K01716	*fabA*	3-hydroxydecanoyl-[acyl-carrier-protein] dehydratase [EC:4.2.1.60]
Fatty acid biosynthesis	S23_00860	K01963	*accD*	acetyl-CoA carboxylase carboxyl transferase subunit beta [EC:6.4.1.2]
Fatty acid biosynthesis	S23_06100	K01962	*accA*	acetyl-CoA carboxylase carboxyl transferase subunit alpha [EC:6.4.1.2]
Fatty acid biosynthesis	S23_08970	K00059		putative 3-oxoacyl-[acyl-carrier-protein] reductase
Fatty acid biosynthesis	S23_08990	K00059	*fabG*	3-oxoacyl-[acyl-carrier protein] reductase [EC:1.1.1.100]
Fatty acid biosynthesis	S23_09040	K00059		putative oxidoreductase protein
Fatty acid biosynthesis	S23_10650	K00059		oxidoreductase
Fatty acid biosynthesis	S23_13540	K00645	*fabD*	[acyl-carrier-protein] S-malonyltransferase [EC:2.3.1.39]
Fatty acid biosynthesis	S23_14920	K00059		short-chain dehydrogenase/reductase SDR
Fatty acid biosynthesis	S23_26180	K00059		putative 3-oxoacyl-[acyl-carrier-protein] reductase
Fatty acid biosynthesis	S23_28390	K00059		short chain dehydrogenase
Fatty acid biosynthesis	S23_31900	K00648	*fabH*	3-oxoacyl-[acyl-carrier-protein] synthase III [EC:2.3.1.180]
Fatty acid biosynthesis	S23_33370	K02372	*fabZ*	3R-hydroxymyristoyl ACP dehydrase [EC:4.2.1.-]
Fatty acid biosynthesis	S23_39410	K01716	*fabA*	3-hydroxydecanoyl-[acyl-carrier-protein] dehydratase [EC:4.2.1.60]
Fatty acid biosynthesis	S23_39920	K02160	*accB, bccP*	acetyl-CoA carboxylase biotin carboxyl carrier protein
Fatty acid biosynthesis	S23_39930	K01961	*accC*	acetyl-CoA carboxylase, biotin carboxylase subunit [EC:6.4.1.2 6.3.4.14]
Fatty acid biosynthesis	S23_41730	K09458	*fabF*	3-oxoacyl-[acyl-carrier-protein] synthase II [EC:2.3.1.179]
Fatty acid biosynthesis	S23_41750	K00059	*fabG*	3-oxoacyl-[acyl-carrier protein] reductase [EC:1.1.1.100]
Fatty acid biosynthesis	S23_41760	K00645	*fabD*	[acyl-carrier-protein] S-malonyltransferase [EC:2.3.1.39]
Fatty acid biosynthesis	S23_42340	K00059		short chain dehydrogenase
Fatty acid biosynthesis	S23_43210	K00059	*fabG*	3-oxoacyl-[acyl-carrier protein] reductase [EC:1.1.1.100]
Fatty acid biosynthesis	S23_44390	K02372		hydroxymyristoyl-acyl carrier protein dehydratase
Fatty acid biosynthesis	S23_44400	K09458	*fabF*	3-oxoacyl-[acyl-carrier-protein] synthase II [EC:2.3.1.179]
Fatty acid biosynthesis	S23_44410	K09458	*fabF*	3-oxoacyl-[acyl-carrier-protein] synthase II [EC:2.3.1.179]
Fatty acid biosynthesis	S23_49720	K00059		putative oxidoreductase
Fatty acid biosynthesis	S23_52530	K00059	*fabG*	3-oxoacyl-[acyl-carrier protein] reductase [EC:1.1.1.100]
Fatty acid biosynthesis	S23_53350	K00208	*fabI*	enoyl-[acyl-carrier protein] reductase I [EC:1.3.1.9]
Fatty acid biosynthesis	S23_54560	K09458	*fabF*	3-oxoacyl-[acyl-carrier-protein] synthase II [EC:2.3.1.179]
Fatty acid biosynthesis	S23_63450	K00059		putative 3-oxoacyl-acyl-carrier-protein] reductase
Fatty acid biosynthesis	S23_65650	K00059	*fabG*	3-oxoacyl-[acyl-carrier protein] reductase [EC:1.1.1.100]
Fatty acid metabolism	S23_02800	K01692	*paaG*	enoyl-CoA hydratase [EC:4.2.1.17]
Fatty acid metabolism	S23_05760	K00626	*atoB*	acetyl-CoA acetyltransferase [EC:2.3.1.9]
Fatty acid metabolism	S23_06590	K01692	*paaG*	enoyl-CoA hydratase [EC:4.2.1.17]
Fatty acid metabolism	S23_06610	K01692	*paaG*	enoyl-CoA hydratase [EC:4.2.1.17]
Fatty acid metabolism	S23_06630	K00249		putative acyl-CoA dehydrogenase
Fatty acid metabolism	S23_06870	K00128		aldehyde dehydrogenase (NAD+) [EC:1.2.1.3]
Fatty acid metabolism	S23_06880	K00249		putative acyl-CoA dehydrogenase
Fatty acid metabolism	S23_07750	K01782	*fadJ*	3-hydroxyacyl-CoA dehydrogenase / enoyl-CoA hydratase / 3-hydroxybutyryl-CoA epimerase [EC:1.1.1.35 4.2.1.17 5.1.2.3]
Fatty acid metabolism	S23_07760	K00626	*atoB*	acetyl-CoA acetyltransferase [EC:2.3.1.9]
Fatty acid metabolism	S23_08560	K01897		putative fatty-acid--CoA ligase
Fatty acid metabolism	S23_08840	K01692	*paaG*	enoyl-CoA hydratase [EC:4.2.1.17]
Fatty acid metabolism	S23_08870	K00121	*adhC*	S-(hydroxymethyl)glutathione dehydrogenase / alcohol dehydrogenase [EC:1.1.1.284 1.1.1.1]
Fatty acid metabolism	S23_09440	K01692	*paaG*	enoyl-CoA hydratase [EC:4.2.1.17]
Fatty acid metabolism	S23_09670	K07516	*fadN*	3-hydroxyacyl-CoA dehydrogenase [EC:1.1.1.35]
Fatty acid metabolism	S23_09680	K01897	*fadD*	long-chain acyl-CoA synthetase [EC:6.2.1.3]
Fatty acid metabolism	S23_15020	K00249		putative acyl-CoA dehydrogenase
Fatty acid metabolism	S23_15230	K01692	*paaG*	enoyl-CoA hydratase [EC:4.2.1.17]
Fatty acid metabolism	S23_20220	K01692	*paaG*	enoyl-CoA hydratase [EC:4.2.1.17]

Fatty acid metabolism	S23_20800	K00121	*adhC*	S-(hydroxymethyl)glutathione dehydrogenase / alcohol dehydrogenase [EC:1.1.1.284 1.1.1.1]
Fatty acid metabolism	S23_21190	K00626	*atoB*	acetyl-CoA acetyltransferase [EC:2.3.1.9]
Fatty acid metabolism	S23_21940	K01692	*paaG*	enoyl-CoA hydratase [EC:4.2.1.17]
Fatty acid metabolism	S23_22060	K00001	*adh*	alcohol dehydrogenase [EC:1.1.1.1]
Fatty acid metabolism	S23_22280	K01692	*paaG*	enoyl-CoA hydratase [EC:4.2.1.17]
Fatty acid metabolism	S23_22320	K01692	*paaG*	enoyl-CoA hydratase [EC:4.2.1.17]
Fatty acid metabolism	S23_22820	K01897	*fadD*	long-chain acyl-CoA synthetase [EC:6.2.1.3]
Fatty acid metabolism	S23_23760	K00128		aldehyde dehydrogenase (NAD+) [EC:1.2.1.3]
Fatty acid metabolism	S23_25980	K00001	*adh*	alcohol dehydrogenase [EC:1.1.1.1]
Fatty acid metabolism	S23_26450	K01692	*paaG*	enoyl-CoA hydratase [EC:4.2.1.17]
Fatty acid metabolism	S23_26590	K01692		3-hydroxybutyryl-CoA dehydratase
Fatty acid metabolism	S23_28520	K00248	*bcd*	butyryl-CoA dehydrogenase [EC:1.3.99.2]
Fatty acid metabolism	S23_29420	K00626	*atoB*	acetyl-CoA acetyltransferase [EC:2.3.1.9]
Fatty acid metabolism	S23_32210	K01692	*paaG*	enoyl-CoA hydratase [EC:4.2.1.17]
Fatty acid metabolism	S23_32850	K00001	*adh*	Iron-containing alcohol dehydrogenase [EC:1.1.1.1]
Fatty acid metabolism	S23_33100	K00001	*adh*	alcohol dehydrogenase [EC:1.1.1.1]
Fatty acid metabolism	S23_41610	K00001	*adh*	putative NAD-dependent alcohol dehydrogenase [EC:1.1.1.1]
Fatty acid metabolism	S23_42830	K01692	*paaG*	enoyl-CoA hydratase [EC:4.2.1.17]
Fatty acid metabolism	S23_42840	K00248	*bcd*	butyryl-CoA dehydrogenase [EC:1.3.99.2]
Fatty acid metabolism	S23_42890	K01692	*paaG*	enoyl-CoA hydratase [EC:4.2.1.17]
Fatty acid metabolism	S23_42900	K00626	*atoB*	acetyl-CoA acetyltransferase [EC:2.3.1.9]
Fatty acid metabolism	S23_43100	K00626	*atoB*	acetyl-CoA acetyltransferase [EC:2.3.1.9]
Fatty acid metabolism	S23_43840	K00248	*bcd*	butyryl-CoA dehydrogenase [EC:1.3.99.2]
Fatty acid metabolism	S23_44870	K00001	*adh*	Alcohol dehydrogenase GroES domain protein [EC:1.1.1.1]
Fatty acid metabolism	S23_49640	K01692	*paaG*	enoyl-CoA hydratase [EC:4.2.1.17]
Fatty acid metabolism	S23_50120	K00249		putative acyl-CoA dehydrogenase
Fatty acid metabolism	S23_50610	K01692	*paaG*	enoyl-CoA hydratase [EC:4.2.1.17]
Fatty acid metabolism	S23_50620	K01897		acyl-CoA synthetase
Fatty acid metabolism	S23_50630	K01692	*paaG*	enoyl-CoA hydratase [EC:4.2.1.17]
Fatty acid metabolism	S23_50660	K00249		acyl-CoA dehydrogenase [EC:1.3.99.3]
Fatty acid metabolism	S23_50700	K01692	*paaG*	enoyl-CoA hydratase [EC:4.2.1.17]
Fatty acid metabolism	S23_50730	K00249		acyl-CoA dehydrogenase [EC:1.3.99.3]
Fatty acid metabolism	S23_50770	K00626	*atoB*	acetyl-CoA acetyltransferase [EC:2.3.1.9]
Fatty acid metabolism	S23_51680	K00529	*hcaD*	ferredoxin--NAD+ reductase [EC:1.18.1.3]
Fatty acid metabolism	S23_51690	K01692	*paaG*	enoyl-CoA hydratase [EC:4.2.1.17]
Fatty acid metabolism	S23_51810	K00128		aldehyde dehydrogenase (NAD+) [EC:1.2.1.3]
Fatty acid metabolism	S23_52100	K01692	*paaG*	enoyl-CoA hydratase [EC:4.2.1.17]
Fatty acid metabolism	S23_52140	K00001	*adh*	NAD-dependent alcohol dehydrogenase [EC:1.1.1.1]
Fatty acid metabolism	S23_53170	K01692		putative enoyl-CoA hydratase protein
Fatty acid metabolism	S23_53480	K00252	*gcdH*	glutaryl-CoA dehydrogenase [EC:1.3.99.7]
Fatty acid metabolism	S23_55820	K01782		putative fatty acid oxidation complex alpha subunit
Fatty acid metabolism	S23_55830	K00626	*atoB*	acetyl-CoA acetyltransferase [EC:2.3.1.9]
Fatty acid metabolism	S23_55840	K06445	*fadE*	acyl-CoA dehydrogenase [EC:1.3.99.-]
Fatty acid metabolism	S23_57030	K05939	*aas*	acyl-[acyl-carrier-protein]-phospholipid O-acyltransferase / long-chain-fatty-acid--[acyl-carrier-protein] ligase [EC:2.3.1.40 6.2.1.20]
Fatty acid metabolism	S23_57920	K01692	*paaG*	enoyl-CoA hydratase [EC:4.2.1.17]
Fatty acid metabolism	S23_58960	K01692	*paaG*	enoyl-CoA hydratase [EC:4.2.1.17]
Fatty acid metabolism	S23_58980	K00249		acyl-CoA dehydrogenase [EC:1.3.99.3]
Fatty acid metabolism	S23_60100	K00626	*atoB*	acetyl-CoA acetyltransferase [EC:2.3.1.9]
Fatty acid metabolism	S23_61840	K00001	*adh*	alcohol dehydrogenase [EC:1.1.1.1]
Fatty acid metabolism	S23_62250	K00128		aldehyde dehydrogenase (NAD+) [EC:1.2.1.3]
Fatty acid metabolism	S23_63640	K01692		putative enoyl-CoA hydratase
Fatty acid metabolism	S23_63670	K00626	*atoB*	acetyl-CoA acetyltransferase [EC:2.3.1.9]
Fatty acid metabolism	S23_63680	K01897		acyl-CoA synthetase
Fatty acid metabolism	S23_64820	K01897		acyl-CoA synthetase
Fatty acid metabolism	S23_65090	K01897	*fadD*	long-chain acyl-CoA synthetase [EC:6.2.1.3]
Fatty acid metabolism	S23_65860	K01692		enoyl-CoA hydratase/isomerase
Fatty acid metabolism	S23_66190	K01692		Enoyl-CoA hydratase/isomerase
Fatty acid metabolism	S23_67360	K01692	*paaG*	enoyl-CoA hydratase [EC:4.2.1.17]
Fatty acid metabolism	S23_68130	K01897		acyl-CoA synthetase
Fatty acid metabolism	S23_68250	K00249		acyl-CoA dehydrogenase [EC:1.3.99.3]
Fatty acid metabolism	S23_68550	K00249		acyl-CoA dehydrogenase [EC:1.3.99.3]

Table S9. Comparison of the genes for nitrogen metabolism between *Bradyrhizobium* sp. S23321 and its close relatives.

Strains	Genes						
	nosZ	*aspA*	L-asparaginase	asparagine synthase	*gdh*	*nit*	*nadE*
Bradyrhizobium sp. S23321	-	S23_64740	-	-	-	-	S23_44610
Bradyrhizobium japonicum USDA110	blr0315	blr1326	bll4950	bll6010, blr6298	blr7995	blr3397	blr3780
Bradyrhizobium sp. BTAi1	BBta_6008	-	-	BBta_3705, BBta_5624, BBta_7206	BBta_2839	-	BBta_3495
Bradyrhizobium sp. ORS278	-	-	-	BRADO0860, BRADO4822, BRADO5156	BRADO2494	-	-

Note: *nosZ*, nitrous oxide (N_2O) reductase (EC:1.7.99.6); *aspA*, aspartate ammonia lyase (EC:4.3.1.1); L-asparaginase, EC:3.5.1.1; asparagine synthase, EC:6.3.5.4; *gdh*, glutamate dehydrogenase (EC:1.4.1.2) or (EC:1.4.1.3); *nit*, nitrilase (EC:3.5.5.1); *nadE*, NAD+ synthase (EC:6.3.1.5).

Table S10. Comparison of genes for L-Asn-tRNA biosynthesis of S23321 and its close relatives.

Strains	Genes			
	asnS	*gatA*	*gatB*	*gatC*
Bradyrhizobium sp. S23321	-	S23_31310	S23_31330	S23_31290
Bradyrhizobium japonicum USDA110	-	bll5089	bll5087	bsl5090
Bradyrhizobium sp. BTAi1	-	BBta_4711	BBta_4709	BBta_4713
Bradyrhizobium sp. ORS278	-	BRADO4489	BRADO4486	BRADO4491

Note: *asnS*, asparaginyl-tRNA synthetase (EC:6.1.1.22); *gatA*, aspartyl/glutamyl-tRNA amidotransferase subunit A (EC:6.3.5.6; EC:6.3.5.7); *gatB*, aspartyl/glutamyl-tRNA amidotransferase subunit B (EC:6.3.5.6; EC:6.3.5.7); *gatC,* aspartyl/glutamyl-tRNA amidotransferase subunit C (EC:6.3.5.6; EC:6.3.5.7).

Table S11. Comparison of genes for degradation of vanillin and related compounds between Bradyrhizobium sp. S23321 and B. japonicum USDA110.

Genes of *Bradyrhizobium japonicum* USDA 110		Gene ID of *Bradyrhizobium* sp. S23321	Start and end position (bp) in S23321 genome		Amino acid identity (%)*
hcaB1	; periplasmic nitrate reductase protein	S23_00210	27,435	28,928	33
hcaB2	; vanillin: NAD oxidoreductase	S23_00210	27,435	28,928	35
vanA1	; vanillate O-demethylase oxygenase subunit	S23_56180	5,788,215	5,789,264	94
vanB1	; putative dioxygenase subunit	S23_56160	5,786,474	5,787,454	87
vanA2	; probable dioxygenase	S23_67440	6,993,093	6,994,451	92
pobA1	; 4-hydroxybenzoate hydroxylase	S23_56910	5,887,496	5,888,668	87
pobA2	; 4-hydroxybenzoate hydroxylase	S23_09490	1,002,452	1,003,621	48
pcaG1	; protocatechuate 3,4-dioxygenase alpha chain	S23_56960	5,891,930	5,892,553	87
pcaH1	; protocatechuate 3,4-dioxygenase beta chain	S23_56970	5,892,559	5,893,347	94
pcaG2	; protocatechuate 3,4-dioxygenase alpha chain	S23_68850	7,155,946	7,156,548	86
pcaH2	; catechol 1,2-dioxygenase	S23_46820	4,757,823	4,758,707	37
pcaH3	; protocatechuate 3,4-dioxygenase beta chain	S23_68860	7,156,550	7,157,251	93
pcaB	; 3-carboxy-cis,cis-muconate cycloisomerase	S23_25870	2,716,341	2,717,696	87
pcaC	; gamma-carboxymuconolactone decarboxylase	S23_25850	2,715,036	2,715,428	89
pcaD	; B-ketoadipate enol-lactone hydrolase	S23_25860	2,715,530	2,716,312	91
pcaI1	; 3-oxoadipate CoA-transferase subunit A	S23_12290	1,303,808	1,304,545	94
pcaJ1	; 3-oxoadipate CoA-transferase subunit B	S23_12300	1,304,545	1,305,195	94
pcaI2	; 3-oxoadipate CoA-transferase subunit A	S23_12290	1,303,808	1,304,545	42
pcaJ2	; 3-oxoadipate CoA-transferase subunit B	S23_12300	1,304,545	1,305,195	52
pcaF	; beta-ketoadipyl CoA thiolase	S23_68870	7,157,386	7,158,594	95
mxaF	; methanol dehydrogenase large subunit-like protein	S23_20820	2,195,784	2,197,592	89
gfa	; glutathione-dependent formaldehyde-activating enzyme	S23_20790	2,193,415	2,193,978	94
flhA	; alcohol dehydrogenase class III	S23_20800	2,194,012	2,195,121	96
fgh	; esterase D (S-formylglutathione hydrolase)	S23_21090	2,218,425	2,219,270	91
fdhF	; formate dehydrogenase alpha subunit	S23_48660	4,972,045	4,974,918	93
fdhD	; formate dehydrogenase	S23_48670	4,974,923	4,975,753	84

* Amino acid sequence identity as compared with those of B. japonicum USDA110.

Table S12. Non-coding RNA in *Bradyrhizobium* sp. S23321

Feature	start	End	Strand	/db_xref=	/gene=	/locus_tag=	/product=	/note=	Sequence	Aanalysis of fRNAdb: Homologous strain (accession number)	Aanalysis of fRNAdb: Identity	Aanalysis of fRNAdb: fRNAdb ID (ver. 3.4)
ncRNA	1170982	1171142	+			CGA1_M002	6S RNA	6S RNA as predicted by Rfam v8.1 (RF00013), score 78.27	GCGAGGCTGCGACGCGCGTCCG<br>GAGCCATATATCCCCGGGGCCT<br>TATCGATCCTTTAGGGAACTGTC<br>CCTGTTCGGGCCCGTGGGCCCG<br>AGCATATGGTGCCCACCTACTTT<br>CGTAGGGAACTCCGGGATCGAG<br>TGCTTCAACGGCGTTCGCGGCT<br>TCGCA	*Bradyrhizobium japonicum* USDA 110 (BA00040)	156/161 (96%)	FR188766
misc_fea	1741550	1741694	−			CGA1_M003		TPP riboswitch (THI element) as predicted by Rfam v8.1 (RF00059), score 65.23	AGCCGTTCCGAGGGGTGCTCCG<br>AGGAGGAGCTGAGATACCGCTA<br>AATGGGCAAAGCTTGCAAATCG<br>GCAGGCCTTCCCAGGACCGCGG<br>TGACCCTTTGAACCTGATCCGG<br>GTCATGCCGGCGAAGGGACAGG<br>GATGTTGCAGACG			
ncRNA	1791001	1791481	−		*rnpB*	CGA1_M004	RNase P class A		TCAGTCGGCCGGACGGCCGCTC<br>CGGCAACGGTCGAAAGGCCGCC<br>GGGGAGGAAAGTCCGGGCTCCC<br>TTGACATACGGTGCCGGATAAC<br>GTCCGGCGGGGGCGACCCCAGG<br>GAAAGTGCCACAGAGAACGAAC<br>CGCCCGCCGTCGCCCCTTCGGG<br>GCTATGGCGCGACAAGCCCGCA<br>AGGCTCGCCGCGCCGAAGCTGC<br>GCAAGCAGCGTAGGCGGGTAAG<br>GGTGAAAAGGTGCGGTAAGAGC<br>GCACCGCGGCTCCGGCAACGGA<br>GCCGGCATGGTAAACCCCACCG<br>GGAGCAAAACCGAATAGGGACG<br>GCATAGCGGGCTGTTCGCGCAA<br>GCGATAACGCCGCGGGGCGATG<br>TCAGGCCCGCCGTCCGGGTAGG<br>TTGCTCGAGGCCATGTGCAAAC<br>ATGGTCCCAGAGGAATGGCCGT<br>CACGTATCGTGCGCGCAAGCGG<br>ACGGTGCCTTACAGAACCCGGC<br>TTACAGGCCGGCTGATATT			
misc_fea	1847699	1847796	−			CGA1_M005		repression of heat shock gene expression (ROSE) element as predicted by Rfam v8.1 (RF00435), score 63.34	CCCGGCGCCTGACGGGTCGGGA<br>AAGATTGAGACGGGCACCGGTC<br>GTGTCCGGTGCCGCTCGTATCC<br>ATTTTGCTCCTTGGAGGATCTGG<br>CTATGCGCA	Bradyrhizobium japonicum (AJ010144)	92/98 (93%)	FR283704
misc_fea	2622890	2622985	−			CGA1_M006		glycine riboswitch as predicted by Rfam v8.1 (RF00504), score 33.96	TTGACGACATCTGGAAAGAGGC<br>ACTGGCGGGTTTAAGACCCGGA<br>CAGCGTCCGCCGACGGGATAAT<br>ACTCTCAGGCACAGCGACAGAT<br>GGGGCTTC	Bradyrhizobium japonicum USDA 110 (BA00040)	81/89 (91%)	FR303517
misc_fea	2622983	2623075	−			CGA1_M007		glycine riboswitch as predicted by Rfam v8.1 (RF00504), score 62.24	TACGGCCACGTCGGGAGAGACC<br>GGCTTTGGGTAGCCGGCGCCGA<br>AGGAGCAACCGCCCCGGAAACT<br>CTCAGGCAAAAGGACCGCGTGG<br>CTTTG	Bradyrhizobium japonicum USDA 110 (BA00040)	91/93 (97%)	FR235387
misc_fea	2781058	2781162	−			CGA1_M008		glycine riboswitch as predicted by Rfam v8.1 (RF00504), score 58.79	CGCGACGATGCGGGAGAGATCG<br>CAACGAAGGCTTCCAGCCTTCC<br>TTGCGGCGCCGACGGAGCAACC<br>GCCCCGGAAACTCTCAGGCAAA<br>AGGACTGTATCGTCAGG			
misc_fea	3487676	3487892	+			CGA1_M009		cobalamin riboswitch as predicted by Rfam v8.1 (RF00174), score 138.74	CGTAGATTGCTACGTGACGGTT<br>CTCCTCACGGAGATCAAAAGGG<br>AACGTGGTGCGGGAATGTCCCA<br>ACTTTCGGGATCGTCTCAATGC<br>CATGGCTGCCCCCGCAACTGTA<br>AGCGGTGAATCTCTCGTCATAT<br>GCCACTGGGAATCTCGGTCCCG<br>GGAAGGCGACGCAGAGGTAACG<br>ACCCGCGAGCCAGGAGACCTGC<br>CGTCAGCCGTGGTCACACG	Bradyrhizobium japonicum USDA 110 (BA00040)	200/217 (92%), Gaps = 2/217	FR042467
misc_fea	3941182	3941276	−			CGA1_M010		suhB as predicted by Rfam v8.1 (RF00519), score 26.46	CACCGCAACCCTCCTTGGGCGT<br>TTCCTCCCTAGACTTGGGCCGC<br>TTGTGACGTGCCTCCCGACTGC<br>AAAGAGACACTACCACTTACTG<br>ATTGTTG			
misc_fea	4810365	4810567	−			CGA1_M011		cobalamin riboswitch as predicted by Rfam v8.1 (RF00174), score 139.18	ATCCTAGATGGCCGTGACGGTT<br>TCCCCGAGAGGGGATGAAAAGG<br>GAATGCGGTGCGGGGACGTTGT<br>CCCCAATGCCGTGGCTGCCCCC<br>GCAACTGTAAGCGGCGAATCTT<br>GCGTCACGAGCCACTGGGTATC<br>TCGGTCCCGGGAAGGCGACGCA<br>AGGTGACGACCCGCGAGCCAGG<br>AGACCTGCCGTCAGCCGTGGTC<br>ACACG	Bradyrhizobium japonicum USDA 110 (BA00040)	187/205 (91%)	FR398290
misc_fea	4813797	4814016	−			CGA1_M012		cobalamin riboswitch as predicted by Rfam v8.1 (RF00174), score 102.51	GGCACACAGGAGATGCATGGTG<br>CTCGACGCGGCGCAAAGCGCCG<br>GAGCATAATCGGGAATGGGGAT<br>GGGCGGACCCAGTTGCGGCGCC<br>CCAAACCCCAGCCGCCCCCGCG<br>ACTGTAAGCGGTAAGGGACTCC<br>GAACCGCCACTGGGCCGCAAGG<br>CTCGGGAAGGCCGGAGAACCCA<br>GTGAGCCGCGAGCCAGGAGACC<br>GGCCGTGCGTGTTTTGAGGCCA	Bradyrhizobium japonicum USDA 110 (BA00040)	208/221 (94%)	FR089286

misc_fea	4827149	4827371	−			CGA1_M013		cobalamin riboswitch as predicted by Rfam v8.1 (RF00174), score 93.25	AAATGGTGACCCGGCGTTGGTTCCTGTCCTATGACAGGCGAAGAGGGAATGCGATAGGGTCCGAATCTGCAAGATTCGGGTTCAAACTGCAGCCGCCCCCGCGACCGTGACCGGAGAGATGTCCGAAGCCACTGATCCCCCAGGGGATCGGGAAGGCGGGGATCGAAGGGCCAAAAGCCCTGCTCCGCAAGCCGGGAGACCTGCCAGCGCGGACGATTTTTGG	Bradyrhizobium japonicum USDA 110 (BA00040)	207/220 (94%), Gaps = 2/220 (0%)	FR226034
misc_fea	5435974	5436051	+			CGA1_M014		suhB as predicted by Rfam v8.1 (RF00519), score 50.5	TCCGATTGCTTTGCTCGGGCGTTTCCTCCCTAGACTTGGGCCGCTTGTCACAAACAAGCGGCTCTTCTTTTTTGTACA	Bradyrhizobium japonicum USDA 110 (BA00040)	72/77 (94%)	FR328619
misc_fea	6215884	6215936	−			CGA1_M015		serC leader as predicted by Rfam v8.1 (RF00517), score 65.51	CGGCGGGACACCTCCCCCCAACGGGAGGCTTACTATCTGGAAGGGTAGAATAT			
misc_fea	6279391	6279550	−			CGA1_M016		flavin mononucleotide (FMN) riboswich (FMN element) as predicted by Rfam v8.1 (RF00050), score 110.43	CGATGTTCTCAGGGCGGGGTGAAAGTCCCCACCGGCGGTAAGGGCCGAAAGGCCTAAGCCCGCGAGCGCCTTCCCCAAGGTTTCTTGCCAAGGGGAGGGGTCAGCAGATTCGGTGCGACTCCGAAGCCGACGGTTAAAGTCCGGATGAAAGAGAACGGTC			
misc_fea	6536167	6536244	−			CGA1_M017		suhB as predicted by Rfam v8.1 (RF00519), score 67.45	TACCGCTGCCCTCCTTGGGCGTTTCCTCCCTAGACTTGGGCCGCTTCTTCATCAGAAGCGGCCCTTTTTTCTTATTTG			
misc_fea	6569748	6570208	+			CGA1_M018		speF leader as predicted by Rfam v8.1 (RF00518), score 188.88	CTTGCCCAATTTCGCACGTGCCTGTGGTCGTGTGTCAGTCGGTAGGTATCCGGACAAGAGGCCGGACAGCCGCCAAGGGGTGAAGAAGCCGAGGGGCTCTTCGGAAGTGCGGAAGTCGAAAGACCCTGAAGTCTGAAGACTGAAAGCAAACCCGGAGCGTCCAGCATCTCTCTCAAAGAGATGCCTTGTCGAAGGTGACTTCACTTTTTGCAACCGTGACTGGCAGCCGGAGGCGAACCGGCGCACCCCGCTCTCAACGGGGGACGCGACTTAAAGCAACGACGGATCGGGCTTTTTTGGTCTCTACCGGCAGTCCAACGCCGGCGCGGGCTACTGAAAAGGCTTGTCCTTCATTGCCAGGTGTGCGGGCGGGAAATTCCCAACCAATCCACGGCAGCACAGTCTGATTTGAGTCTTTGGCTCGTCGCGCCTCCATCGCGTGATCTGGCCGGGGCGCTTAT	Bradyrhizobium japonicum USDA 110 (BA00040)	425/460 (92%)	FR081351
misc_fea	6638444	6638523	−			CGA1_M019		S-adenosylmethionine (SAM) riboswitch (alpha-proteobacteria) as predicted by Rfam v8.1 (RF00521), score 79.68	TGCCTGTTCCGTGGTCATTTGAGCCGGCCGGCTTGCAGCCACGTTAAAAAACTCGCTAAACAGGCCGGGGACGCTTCCGA	Bradyrhizobium japonicum USDA 110 (BA00040)	80 /80 (100%)	FR000750
ncRNA	6893107	6893204	−		*ffs*	CGA1_M020	RNA component of signal recognition particle (SRP)		GGAGATTGGCGGTGGACGAGCCACTCGCCAACCGGGTCAGGTCCGGAAGGAAGCAGCCCTAACGAGGTCCGGATCGGGTCGCTCGTCAGTCTCCTACT	Bradyrhizobium japonicum USDA 110 (BA00040)	97/97 (100%)	FR385819
misc_fea	6959302	6959381	+			CGA1_M021		S-adenosylmethionine (SAM) riboswitch (alpha-proteobacteria) as predicted by Rfam v8.1 (RF00521), score 84.97	GACTTATCCCGTGGTGATTTGAGCCGGCCGGCTTGCAGCCACGTTAAATAAGTCGCTAAACAGGCCGGGGACCTCTGTGA	Bradyrhizobium japonicum USDA 110 (BA00040)	79/80 (99%)	FR014451
